# Supplementary material for: On the Species Identification of Two Non-Native Tilapia Species, Including the First Record of a Feral Population of Oreochromis aureus (Steindachner, 1864) in South Korea
Source: Animals (Basel). 2023 Apr 14;13(8):1351. doi: 10.3390/ani13081351 (PMC10134965; doi:10.3390/ani13081351)
Supplement: Supplementary file 1 [file animals-13-01351-s001.zip › animals-2237905-supplementary.pdf]

Supplementary Materials

Table S1. NCBI GenBank accession numbers used in this study to trace the origin of the invasive populations of *Oreochromis* spp..

GenBank accession number	Scientific name	1-631 bp
DQ426665	*O. aureus*	TTGAGCCGGAATAGTAGGAACCGCGCTAAGCCTCCTAATTCGGGCAGAACTAAGCCAGCCCGG<br>CTCTCTCCTCGGAGACGACCAGATTTATAATGTAATTGTTACAGCACATGCTTTTGTAATAATTTT<br>CTTTGTAGTAATGCCAATTATGATTGGAGGCTTTGGAAACTGACTAGTACCACTCATGATTGGTG<br>CCCCAGATATGGCCTTCCCTCGAATGAACAACATGAGTTTCTGACTCCTCCCTCCCTCATTCCTC<br>CTCCTCCTCGCCTCATCTGGAGTCGAAGCAGGTGCCGGCACAGGGTGAACTGTTTACCCCCCG<br>CTCGCAGGCAATCTTGCCCATGCTGGGCCTTCTGTCGACTTAACCATCTTCTCCCTCCACTTGGC<br>CGGGGTGTCATCTATTCTAGGCGCAATTAATTTCATTACAACAATCATTAACATGAAACCCCCCG<br>CCATCTCTCAATATCAAACACCCCTATTTGTATGGTCCGTTCTAATTACCGCAGTATTACTTCTTC<br>TATCCCTACCCGTTCTTGCCGCCGGCATCACAATACTTCTCACAGACCGAAACCTAAACACAAC<br>CTTCTTTGATCCTGCCGGAGGAGGAGACCCCATCCTTTACCAACACTTA
DQ426666	*O. aureus* × *O. niloticus*	TTGAGCCGGAATAGTAGGAACTGCACTAAGCCTCCTAATTCGGGCAGAACTAAGCCAGCCCGG<br>CTCTCTTCTCGGAGACGACCAAATCTATAATGTAATTGTTACAGCACATGCTTTCGTAATAATTTT<br>CTTTATAGTAATACCAATTATGATTGGAGGCTTTGGAAACTGACTAGTACCCCTCATGATTGGTG<br>CACCAGACATGGCCTTCCCTCGAATAAATAACATGAGCTTTTGACTTCTCCCCCCCTCATTTCTT<br>CTTCTTCTCGCCTCATCTGGAGTCGAAGCAGGTGCCGGCACAGGATGGACTGTTTATCCCCCGC<br>TCGCAGGCAATCTTGCCCACGCTGGACCTTCTGTTGACTTAACCATCTTCTCCCTCCACTTGGC<br>CGGAGTGTCATCTATTTTAGGTGCAATTAATTTTATCACAACCATTATTAACATGAAACCCCCTGC<br>CATCTCCCAATATCAAACACCCCTATTTGTGTGATCCGTCCTAATTACCGCAGTACTACTCCTTCT<br>ATCCCTGCCCGTTCTTGCCGCCGGCATCACAATACTTCTAACAGACCGAAACCTAAACACAACC<br>TTCTTTGACCCTGCCGGAGGAGGAGACCCCATCCTATACCAACACTTA
EU751880	*O. niloticus*	TTGAGCCGGAATAGTAGGAACCGCGCTAAGCCTCCTAATTCGGGCAGAACTAAGCCAGCCCGG<br>CTCTCTCCTCGGAGACGACCAGATTTATAATGTAATTGTTACAGCACATGCTTTTGTAATAATTTT<br>CTTTATAGTAATGCCAATTATGATTGGAGGCTTTGGAAACTGACTAGTACCACTCATGATTGGTG<br>CCCCAGATATGGCCTTCCCTCGAATGAACAACATGAGTTTCTGACTCCTCCCTCCCTCATTCCTC<br>CTCCTCCTCGCCTCATCTGGAGTCGAAGCAGGTGCCGGCACAGGGTGAACTGTTTACCCCCCG<br>CTCGCAGGCAATCTTGCCCATGCTGGGCCTTCTGTCGACTTAACCATCTTCTCCCTCCACTTGGC<br>CGGGGTGTCATCTATTCTAGGCGCAATTAATTTCATTACAACAATCATTAACATGAAACCCCCCG<br>CCATCTCTCAATATCAAACACCCCTATTTGTATGGTCCGTTCTAATTACCGCAGTATTACTTCTTC<br>TATCCCTACCCGTTCTTGCCGCCGGCATCACAATACTTCTCACAGACCGAAACCTAAACACAAC<br>CTTCTTTGATCCTGCCGGAGGAGGAGACCCCATCCTTTACCAACACTTA
EU751881	*O. niloticus*	TTGAGCCGGAATAGTAGGAACCGCGCTAAGCCTCCTAATTCGGGCAGAACTAAGCCAACCCGG<br>CTCTCTCCTCGGAGACGACCAGATTTATAATGTAATTGTTACAGCACATGCTTTTGTAATAATTTT<br>CTTTATAGTAATGCCAATTATGATTGGAGGCTTTGGAAACTGACTAGTACCACTCATGATTGGTG<br>CCCCAGATATGGCCTTCCCTCGAATGAACAACATGAGTTTCTGACTCCTCCCTCCCTCATTCCTC<br>CTCCTCCTCGCCTCATCTGGAGTCGAAGCAGGTGCCGGCACAGGGTGAACTGTTTACCCCCCG<br>CTCGCAGGCAATCTTGCCCATGCTGGGCCTTCTGTCGACTTAACCATCTTCTCTCTCCACTTGGC<br>CGGGGTGTCATCTATTCTAGGCGCAATTAATTTCATTACAACAATCATTAACATGAAACCCCCCG<br>CCATCTCTCAATATCAAACACCCCTATTTGTATGGTCCGTTCTAATTACCGCAGTATTACTTCTTC<br>TATCCCTACCCGTTCTTGCCGCCGGCATCACAATACTTCTCACAGACCGAAACCTAAACACAAC<br>CTTCTTTGATCCTGCCGGAGGAGGAGACCCCATCCTTTACCAACACTTA
EU751882	*O. niloticus*	TTGAGCCGGAATAGTAGGAACCGCGCTAAGCCTCCTAATTCGGGCAGAACTAAGCCAGCCCGG<br>CTCTCTCCTCGGAGACGACCAGATTTATAATGTAATTGTTACAGCACATGCTTTTGTAATAATTTT<br>CTTTATAGTAATGCCAATTATGATTGGAGGCTTTGGAAACTGACTAGTACCACTCATGATTGGTG<br>CCCCAGATATGGCCTTCCCTCGAATGAACAACATGAGTTTCTGACTCCTCCCTCCCTCATTCCTC<br>CTCCTCCTCGCCTCATCTGGAGTCGAAGCAGGTGCCGGCACAGGGTGAACTGTTTACCCCCCG<br>CTCGCAGGCAATCTTGCCCATGCTGGGCCTTCTGTCGACTTAACCATCTTCTCCCTCCACTTGGC<br>CGGGGTGTCATCTATTCTAGGCGCAATTAATTTCATTACAACAATCATTAACATGAAACCCCCCG<br>CCATCTCTCAATATCAAACACCCCTATTTGTATGGTCCGTTCTAATTACCGCAGTATTACTTCTTCT<br>ATCCCTACCCGTTCTTGCCGCCGGCATCACAATACTTCTCACAGACCGAAACCTAAACACAACC<br>TTCTTTGATCCTGCCGGAGGAGGAGACCCCATCCTTTACCAACACTTA
EU751883	*O. niloticus*	TTGAGCCGGAATAGTAGGAACCGCGCTAAGCCTCCTAATTCGGGCAGAACTAAGCCAACCCGG<br>CTCTCTCCTCGGAGACGACCAGATTTATAATGTAATTGTTACAGCACATGCTTTTGTAATAATTTT<br>CTTTATAGTAATGCCAATTATGATTGGAGGCTTTGGAAACTGACTAGTACCACTCATGATTGGTG<br>CCCCAGATATGGCCTTCCCTCGAATGAACAACATGAGTTTCTGACTCCTCCCTCCCTCATTCCTC<br>CTCCTCCTCGCCTCATCTGGAGTCGAAGCAGGTGCCGGCACAGGGTGAACTGTTTACCCCCCG<br>CTCGCAGGCAATCTTGCCCATGCTGGGCCTTCTGTCGACTTAACCATCTTCTCTCTCCACTTGGC<br>CGGGGTGTCATCTATTCTAGGCGCAATTAATTTCATTACAACAATCATTAACATGAAACCCCCCG<br>CCATCTCTCAATATCAAACACCCCTATTTGTATGGTCCGTTCTAATTACCGCAGTATTACTTCTTC<br>TATCCCTACCCGTTCTTGCCGCCGGCATCACAATACTTCTCACAGACCGAAACCTAAACACAAC<br>CTTCTTTGATCCTGCCGGAGGAGGAGACCCCATCCTTTACCAACACTTA

GU702135	*O. niloticus*	TTGAGCCGGAATAGTAGGAACCGCGCTAAGCCTCCTAATTCGGGCAGAACTAAGCCAACCCGG<br>CTCTCTCCTCGGAGACGACCAGATTTATAATGTAATTGTTACAGCACATGCTTTTGTAATAATTTT<br>CTTTATAGTAATGCCAATTATGATTGGAGGCTTTGGAAACTGACTAGTACCACTCATGATTGGTG<br>CCCCAGATATGGCCTTCCCTCGAATGAACAACATGAGTTTCTGACTCCTCCCTCCCTCATTCCTC<br>CTCCTCCTCGCCTCATCTGGAGTCGAAGCAGGTGCCGGCACAGGGTGAACTGTTTACCCCCCG<br>CTCGCAGGCAATCTTGCCCATGCTGGGCCTTCTGTCGACTTAACCATCTTCTCTCTCCACTTGGC<br>CGGGGTGTCATCTATTCTAGGCGCAATTAATTTCATTACAACAATCATTAACATGAAACCCCCCG<br>CCATCTCTCAATATCAAACACCCCTATTTGTATGGTCCGTTCTAATTACCGCAGTATTACTTCTTC<br>TATCCCTACCCGTTCTTGCCGCCGGCATCACAATACTTCTCACAGACCGAAACCTAAACACAAC<br>CTTCTTTGATCCTGCCGGAGGAGGAGACCCCATCCTTTACCAACACTTA
GU702136	*O. niloticus*	TTGAGCCGGAATAGTAGGAACCGCGCTAAGCCTCCTAATTCGGGCAGAACTAAGCCAACCCGG<br>CTCTCTCCTCGGAGACGACCAGATTTATAATGTAATTGTTACAGCACATGCTTTTGTAATAATTTT<br>CTTTATAGTAATGCCAATTATGATTGGAGGCTTTGGAAACTGACTAGTACCACTCATGATTGGTG<br>CCCCAGATATGGCCTTCCCTCGAATGAACAACATGAGTTTCTGACTCCTCCCTCCCTCATTCCTC<br>CTCCTCCTCGCCTCATCTGGAGTCGAAGCAGGTGCCGGCACAGGGTGAACTGTTTACCCCCCG<br>CTCGCAGGCAATCTTGCCCATGCTGGGCCTTCTGTCGACTTAACCATCTTCTCTCTCCACTTGGC<br>CGGGGTGTCATCTATTCTAGGCGCAATTAATTTCATTACAACAATCATTAACATGAAACCCCCCG<br>CCATCTCTCAATATCAAACACCCCTATTTGTATGGTCCGTTCTAATTACCGCAGTATTACTTCTTC<br>TATCCCTACCCGTTCTTGCCGCCGGCATCACAATACTTCTCACAGACCGAAACCTAAACACAAC<br>CTTCTTTGATCCTGCCGGAGGAGGAGACCCCATCCTTTACCAACACTTA
GU702137	*O. niloticus*	TTGAGCCGGAATAGTAGGAACCGCGCTAAGCCTCCTAATTCGGGCAGAACTAAGCCAACCCGG<br>CTCTCTCCTCGGAGACGACCAGATTTATAATGTAATTGTTACAGCACATGCTTTTGTAATAATTTT<br>CTTTATAGTAATGCCAATTATGATTGGAGGCTTTGGAAACTGACTAGTACCACTCATGATTGGTG<br>CCCCAGATATGGCCTTCCCTCGAATGAACAACATGAGTTTCTGACTCCTCCCTCCCTCATTCCTC<br>CTCCTCCTCGCCTCATCTGGAGTCGAAGCAGGTGCCGGCACAGGGTGAACTGTTTACCCCCCG<br>CTCGCAGGCAATCTTGCCCATGCTGGGCCTTCTGTCGACTTAACCATCTTCTCTCTCCACTTGGC<br>CGGGGTGTCATCTATTCTAGGCGCAATTAATTTCATTACAACAATCATTAACATGAAACCCCCCG<br>CCATCTCTCAATATCAAACACCCCTATTTGTATGGTCCGTTCTAATTACCGCAGTATTACTTCTTC<br>TATCCCTACCCGTTCTTGCCGCCGGCATCACAATACTTCTCACAGACCGAAACCTAAACACAAC<br>CTTCTTTGATCCTGCCGGAGGAGGAGACCCCATCCTTTACCAACACTTA
HM882785	*O. niloticus*	TTGAGCCGGAATAGTAGGAACCGCGCTAAGCCTCCTAATTCGGGCAGAACTAAGCCAGCCCGG<br>CTCTCTCCTCGGAGACGACCAGATTTATAATGTAATTGTTACAGCACATGCTTTTGTAATAATTTT<br>CTTTATAGTAATGCCAATTATGATTGGAGGCTTTGGAAACTGACTAGTACCACTCATGATTGGTG<br>CCCCAGATATGGCCTTCCCTCGAATGAACAACATGAGTTTCTGACTCCTCCCTCCCTCATTCCTC<br>CTCCTCCTCGCCTCATCTGGAGTCGAAGCAGGTGCCGGCACAGGGTGAACTGTTTACCCCCCG<br>CTCGCAGGCAATCTTGCCCATGCTGGGCCTTCTGTCGACTTAACCATCTTCTCCCTCCACTTGGC<br>CGGGGTGTCATCTATTCTAGGCGCAATTAATTTCATTACAACAATCATTAACATGAAACCCCCCG<br>CCATCTCTCAATATCAAACACCCCTATTTGTATGGTCCGTTCTAATTACCGCAGTATTACTTCTTC<br>TATCCCTACCCGTTCTTGCCGCCGGCATCACAATACTTCTCACAGACCGAAACCTAAACACAAC<br>CTTCTTTGATCCTGCCGGAGGAGGAGACCCCATCCTTTACCAACACTTA
HM882787	*O. niloticus*	TTGAGCCGGAATAGTAGGAACCGCGCTAAGCCTCCTAATTCGGGCAGAACTAAGCCAGCCCGG<br>CTCTCTCCTCGGAGACGACCAGATTTATAATGTAATTGTTACAGCACATGCTTTTGTAATAATTTT<br>CTTTATAGTAATGCCAATTATGATTGGAGGCTTTGGAAACTGACTAGTACCACTCATGATTGGTG<br>CCCCAGATATGGCCTTCCCTCGAATGAACAACATGAGTTTCTGACTCCTCCCTCCCTCATTCCTC<br>CTCCTCCTCGCCTCATCTGGAGTCGAAGCAGGTGCCGGCACAGGGTGAACTGTTTACCCCCCG<br>CTCGCAGGCAATCTTGCCCATGCTGGGCCTTCTGTCGACTTAACCATCTTCTCCCTCCACTTGGC<br>CGGGGTGTCATCTATTCTAGGCGCAATTAATTTCATTACAACAATCATTAACATGAAACCCCCCG<br>CCATCTCTCAATATCAAACACCCCTATTTGTATGGTCCGTTCTAATTACCGCAGTATTACTTCTTC<br>TATCCCTACCCGTTCTTGCCGCCGGCATCACAATACTTCTCACAGACCGAAACCTAAACACAAC<br>CTTCTTTGATCCTGCCGGAGGAGGAGACCCCATCCTTTACCAACACTTA
HM882898	*O. niloticus*	TTGAGCCGGAATAGTAGGAACCGCGCTAAGCCTCCTAATTCGGGCAGAACTAAGCCAGCCCGG<br>CTCTCTCCTCGGAGACGACCAGATTTATAATGTAATTGTTACAGCACATGCTTTTGTAATAATTTT<br>CTTTATAGTAATGCCAATTATGATTGGAGGCTTTGGAAACTGACTAGTACCACTCATGATTGGTG<br>CCCCAGATATGGCCTTCCCTCGAATGAACAACATGAGTTTCTGACTCCTCCCTCCCTCATTCCTC<br>CTCCTCCTCGCCTCATCTGGAGTCGAAGCAGGTGCCGGCACAGGGTGAACTGTTTACCCCCCG<br>CTCGCAGGCAATCTTGCCCATGCTGGGCCTTCTGTCGACTTAACCATCTTCTCCCTCCACTTGGC<br>CGGGGTGTCATCTATTCTAGGCGCAATTAATTTCATTACAACAATCATTAACATGAAACCCCCCG<br>CCATCTCTCAATATCAAACACCCCTATTTGTATGGTCCGTTCTAATTACCGCAGTATTACTTCTTCT<br>ATCCCTACCCGTTCTTGCCGCCGGCATCACAATACTTCTCACAGACCGAAACCTAAACACAACC<br>TTCTTTGATCCTGCCGGAGGAGGAGACCCCATCCTTTACCAACACTTA
HM882899	*O. niloticus*	TTGAGCCGGAATAGTAGGAACCGCGCTAAGCCTCCTAATTCGGGCAGAACTAAGCCAGCCCGG<br>CTCTCTCCTCGGAGACGACCAGATTTATAATGTAATTGTTACAGCACATGCTTTTGTAATAATTTT<br>CTTTATAGTAATGCCAATTATGATTGGAGGCTTTGGAAACTGACTAGTACCACTCATGATTGGTG<br>CCCCAGATATGGCCTTCCCTCGAATGAACAACATGAGTTTCTGACTCCTCCCTCCCTCATTCCTC<br>CTCCTCCTCGCCTCATCTGGAGTCGAAGCAGGTGCCGGCACAGGGTGAACTGTTTACCCCCCG<br>CTCGCAGGCAATCTTGCCCATGCTGGGCCTTCTGTCGACTTAACCATCTTCTCCCTCCACTTGGC<br>CGGGGTGTCATCTATTCTAGGCGCAATTAATTTCATTACAACAATCATTAACATGAAACCCCCCG<br>CCATCTCTCAATATCAAACACCCCTATTTGTATGGTCCGTTCTAATTACCGCAGTATTACTTCTTC<br>TATCCCTACCCGTTCTTGCCGCCGGCATCACAATACTTCTCACAGACCGAAACCTAAACACAAC<br>CTTCTTTGATCCTGCCGGAGGAGGAGACCCCATCCTTTACCAACACTTA

HM882927	*O. niloticus*	TTGAGCCGGAATAGTAGGAACCGCGCTAAGCCTCCTAATTCGGGCAGAACTAAGCCAGCCCGG<br>CTCTCTCCTCGGAGACGACCAGATTTATAATGTAATTGTTACAGCACATGCTTTTGTAATAATTTT<br>CTTTATAGTAATGCCAATTATGATTGGAGGCTTTGGAAACTGACTAGTACCACTCATGATTGGTG<br>CCCCAGATATGGCCTTCCCTCGAATGAACAACATGAGTTTCTGACTCCTCCCTCCCTCATTCCTC<br>CTCCTCCTCGCCTCATCTGGAGTCGAAGCAGGTGCCGGCACAGGGTGAACTGTTTACCCCCCG<br>CTCGCAGGCAATCTTGCCCATGCTGGGCCTTCTGTCGACTTAACCATCTTCTCCCTCCACTTGGC<br>CGGGGTGTCATCTATTCTAGGCGCAATTAATTTCATTACAACAATCATTAACATGAAACCCCCCG<br>CCATCTCTCAATATCAAACACCCCTATTTGTATGGTCCGTTCTAATTACCGCAGTATTACTTCTTC<br>TATCCCTACCCGTTCTTGCCGCCGGCATCACAATACTTCTCACAGACCGAAACCTAAACACAAC<br>CTTCTTTGATCCTGCCGGAGGAGGAGACCCCATCCTTTACCAACACTTA
HQ024985	*O. niloticus*	TTGAGCCGGAATAGTAGGAACTGCACTAAGCCTCCTAATTCGGGCAGAACTAAGCCAGCCCGG<br>CTCTCTTCTCGGAGACGACCAAATCTATAATGTAATTGTTACAGCACATGCTTTCGTAATAATTTT<br>CTTTATAGTAATACCAATTATGATTGGAGGCTTTGGAAACTGACTAGTACCCCTCATGATTGGTG<br>CACCAGACATGGCCTTCCCTCGAATAAATAACATGAGCTTTTGACTTCTCCCCCCCTCATTTCTT<br>CTTCTTCTCGCCTCATCTGGAGTCGAAGCAGGTGCCGGCACAGGATGGACTGTTTATCCCCCGC<br>TCGCAGGCAATCTTGCCCACGCTGGACCTTCTGTTGACTTAACCATCTTCTCCCTCCACTTGGC<br>CGGAGTGTCATCTATTTTAGGTGCAATTAATTTTATCACAACCATTATTAACATGAAACCCCCTGC<br>CATCTCCCAATATCAAACACCCCTATTTGTGTGATCCGTCCTAATTACCGCAGTACTACTCCTTCT<br>ATCCCTGCCCGTTCTTGCCGCCGGCATCACAATACTTCTAACAGACCGAAACCTAAACACAACC<br>TTCTTTGACCCTGCCGGAGGAGGAGACCCCATCCTATACCAACACTTA
HQ024986	*O. niloticus*	TTGAGCCGGAATAGTAGGAACTGCACTAAGCCTCCTAATTCGGGCAGAACTAAGCCAGCCCGG<br>CTCTCTTCTCGGAGACGACCAAATCTATAATGTAATTGTTACAGCACATGCTTTCGTAATAATTTT<br>CTTTATAGTAATACCAATTATGATTGGAGGCTTTGGAAACTGACTAGTACCCCTCATGATTGGTG<br>CACCAGACATGGCCTTCCCTCGAATAAATAACATGAGCTTTTGACTTCTCCCCCCCTCATTTCTT<br>CTTCTTCTCGCCTCATCTGGAGTCGAAGCAGGTGCCGGCACAGGATGGACTGTTTATCCCCCGC<br>TCGCAGGCAATCTTGCCCACGCTGGACCTTCTGTTGACTTAACCATCTTCTCCCTCCACTTGGC<br>CGGAGTGTCATCTATTTTAGGTGCAATTAATTTTATCACAACCATTATTAACATGAAACCCCCTGC<br>CATCTCCCAATATCAAACACCCCTATTTGTGTGATCCGTCCTAATTACCGCAGTACTACTCCTTCT<br>ATCCCTGCCCGTTCTTGCCGCCGGCATCACAATACTTCTAACAGACCGAAACCTAAACACAACC<br>TTCTTTGACCCTGCCGGAGGAGGAGACCCCATCCTATACCAACACTTA
HQ024987	*O. niloticus*	TTGAGCCGGAATAGTAGGAACTGCACTAAGCCTCCTAATTCGGGCAGAACTAAGCCAGCCCGG<br>CTCTCTTCTCGGAGACGACCAAATCTATAATGTAATTGTTACAGCACATGCTTTCGTAATAATTTT<br>CTTTATAGTAATACCAATTATGATTGGAGGCTTTGGAAACTGACTAGTACCCCTCATGATTGGTG<br>CACCAGACATGGCCTTCCCTCGAATAAATAACATGAGCTTTTGACTTCTCCCCCCCTCATTTCTT<br>CTTCTTCTCGCCTCATCTGGAGTCGAAGCAGGTGCCGGCACAGGATGGACTGTTTATCCCCCGC<br>TCGCAGGCAATCTTGCCCACGCTGGACCTTCTGTTGACTTAACCATCTTCTCCCTCCACTTGGC<br>CGGAGTGTCATCTATTTTAGGTGCAATTAATTTTATCACAACCATTATTAACATGAAACCCCCTGC<br>CATCTCCCAATATCAAACACCCCTATTTGTGTGATCCGTCCTAATTACCGCAGTACTACTCCTTCT<br>ATCCCTGCCCGTTCTTGCCGCCGGCATCACAATACTTCTAACAGACCGAAACCTAAACACAACC<br>TTCTTTGACCCTGCCGGAGGAGGAGACCCCATCCTATACCAACACTTA
HQ024988	*O. niloticus*	TTGAGCCGGAATAGTAGGAACTGCACTAAGCCTCCTAATTCGGGCAGAACTAAGCCAGCCCGG<br>CTCTCTTCTCGGAGACGACCAAATCTATAATGTAATTGTTACAGCACATGCTTTCGTAATAATTTT<br>CTTTATAGTAATACCAATTATGATTGGAGGCTTTGGAAACTGACTAGTACCCCTCATGATTGGTG<br>CACCAGACATGGCCTTCCCTCGAATAAATAACATGAGCTTTTGACTTCTCCCCCCCTCATTTCTT<br>CTTCTTCTCGCCTCATCTGGAGTCGAAGCAGGTGCCGGCACAGGATGGACTGTTTATCCCCCGC<br>TCGCAGGCAATCTTGCCCACGCTGGACCTTCTGTTGACTTAACCATCTTCTCCCTCCACTTGGC<br>CGGAGTGTCATCTATTTTAGGTGCAATTAATTTTATCACAACCATTATTAACATGAAACCCCCTGC<br>CATCTCCCAATATCAAACACCCCTATTTGTGTGATCCGTCCTAATTACCGCAGTACTACTCCTTCT<br>ATCCCTGCCCGTTCTTGCCGCCGGCATCACAATACTTCTAACAGACCGAAACCTAAACACAACC<br>TTCTTTGACCCTGCCGGAGGAGGAGACCCCATCCTATACCAACACTTA
HQ654742	*O. niloticus*	TTGAGCCGGAATAGTAGGAACTGCACTAAGTCTCCTTATTCGGGCAGAACTAAGCCAGCCCGG<br>CTCTCTCCTCGGAGACGACCAGATTTATAATGTAATTGTTACAGCACATGCTTTCGTAATAATTTT<br>CTTTATAGTAATACCAATTATAATTGGAGGGTTTGGAAACTGACTGGTGCCACTTATGATTGGAG<br>CACCAGACATGGCCTTCCCTCGAATAAATAACATGAGTTTTTGACTCCTTCCCCCCTCATTTCTC<br>CTTCTCCTCGCCTCATCCGGAGTCGAAGCAGGAGCCGGTACAGGATGAACTGTTTATCCTCCCC<br>TCGCAGGCAATCTCGCCCACGCTGGACCTTCTGTTGACTTGACCATCTTTTCCCTCCACTTGGC<br>CGGGGTGTCATCTATTTTAGGCGCAATTAATTTTATCACAACCATTATTAACATAAAACCCCCTGC<br>CATCTCTCAATATCAAACACCCCTCTTTGTGTGATCCGTTCTAATTACCGCAGTATTACTCCTACT<br>ATCCCTGCCCGTTCTTGCCGCCGGCATCACAATACTTCTAACAGACCGAAACCTAAACACAACC<br>TTCTTTGACCCTGCCGGAGGAGGAGACCCCATCCTTTACCAACACTTA
HQ654746	*O. niloticus*	TTGAGCCGGAATAGTAGGAACCGCGCTAAGCCTCCTAATTCGGGCAGAACTAAGCCAGCCCGG<br>CTCTCTCCTCGGAGACGACCAGATTTATAATGTAATTGTTACAGCACATGCTTTTGTAATAATTTT<br>CTTTATAGTAATGCCAATTATGATTGGAGGCTTTGGAAACTGACTAGTACCACTCATGATTGGTG<br>CCCCAGATATGGCCTTCCCTCGAATGAACAACATGAGTTTCTGACTCCTCCCTCCCTCATTCCTC<br>CTCCTCCTCGCCTCATCTGGAGTCGAAGCAGGTGCCGGCACAGGGTGAACTGTTTACCCCCCG<br>CTCGCAGGCAATCTTGCCCATGCTGGGCCTTCTGTCGACTTAACCATCTTCTCCCTCCACTTGGC<br>CGGGGTGTCATCTATTCTAGGCGCAATTAATTTCATTACAACAATCATTAACATGAAACCCCCCG<br>CCATCTCTCAATATCAAACACCCCTATTTGTATGGTCCGTTCTAATTACCGCAGTATTACTTCTTC<br>TATCCCTACCCGTTCTTGCCGCCGGCATCACAATACTTCTCACAGACCGAAACCTAAACACAAC<br>CTTCTTTGATCCTGCCGGAGGAGGAGACCCCATCCTTTACCAACACTTA

HQ654747	*O. niloticus*	TTGAGCCGGAATAGTAGGAACTGCATTAAGCCTCCTAATTCGGGCAGAACTAAGCCAGCCCGG<br>CTCTCTCCTCGGAGACGACCAGATTTATAATGTAATTGTTACAGCACATGCTTTCGTAATAATTTT<br>CTTTATAGTAATGCCAATTATAATTGGAGGTTTTGGAAACTGACTAGTGCCACTAATGATTGGTG<br>CACCAGACATGGCCTTCCCTCGAATAAATAACATGAGTTTTTGACTCCTCCCCCCCTCATTTCTC<br>CTTCTCCTCGCCTCATCCGGGGTCGAAGCAGGGGCCGGTACAGGATGGACTGTTTATCCCCCAC<br>TCGCAGGCAATCTCGCCCATGCTGGGCCTTCCGTTGACTTAACCATCTTCTCCCTCCACTTGGCC<br>GGGGTGTCATCTATTTTAGGTGCAATTAATTTTATTACAACCATTATTAACATAAAACCCCCTGCC<br>ATCTCCCAATATCAAACACCCCTCTTTGTATGATCCGTTCTAATTACCGCAGTACTACTCCTACTA<br>TCCCTACCCGTTCTTGCCGCCGGCATCACAATACTTCTAACAGACCGAAACCTAAACACAACCT<br>TCTTTGACCCTGCCGGAGGAGGAGACCCCATCCTTTACCAACACTTA
KC789549	*O. niloticus*	TTGAGCCGGAATAGTAGGAACTGCACTAAGCCTCCTAATTCGGGCAGAACTAAGCCAGCCCGG<br>CTCTCTTCTCGGAGACGACCAAATCTATAATGTAATTGTTACAGCACATGCTTTCGTAATAATTTT<br>CTTTATAGTAATACCAATTATGATTGGAGGCTTTGGAAACTGACTAGTACCCCTCATGATTGGTG<br>CACCAGACATGGCCTTCCCTCGAATAAATAACATGAGCTTTTGACTTCTCCCCCCCTCATTTCTT<br>CTTCTTCTCGCCTCATCTGGAGTCGAAGCAGGTGCCGGCACAGGATGGACTGTTTATCCCCCGC<br>TCGCAGGCAATCTTGCCCACGCTGGACCTTCTGTTGACTTAACCATCTTCTCCCTCCACTTGGC<br>CGGAGTGTCATCTATTTTAGGTGCAATTAATTTTATCACAACCATTATTAACATGAAACCCCCTGC<br>CATCTCCCAATATCAAACACCCCTATTTGTGTGATCCGTCCTAATTACCGCAGTACTACTCCTTCT<br>ATCCCTGCCCGTTCTTGCCGCCGGCATCACAATACTTCTAACAGACCGAAACCTAAACACAACC<br>TTCTTTGACCCTGCCGGAGGAGGAGACCCCATCCTATACCAACACTTA
KC789549.1	*O. niloticus*	TTGAGCCGGAATAGTAGGAACTGCACTAAGCCTCCTAATTCGGGCAGAACTAAGCCAGCCCGG<br>CTCTCTTCTCGGAGACGACCAAATCTATAATGTAATTGTTACAGCACATGCTTTCGTAATAATTTT<br>CTTTATAGTAATACCAATTATGATTGGAGGCTTTGGAAACTGACTAGTACCCCTCATGATTGGTG<br>CACCAGACATGGCCTTCCCTCGAATAAATAACATGAGCTTTTGACTTCTCCCCCCCTCATTTCTT<br>CTTCTTCTCGCCTCATCTGGAGTCGAAGCAGGTGCCGGCACAGGATGGACTGTTTATCCCCCGC<br>TCGCAGGCAATCTTGCCCACGCTGGACCTTCTGTTGACTTAACCATCTTCTCCCTCCACTTGGC<br>CGGAGTGTCATCTATTTTAGGTGCAATTAATTTTATCACAACCATTATTAACATGAAACCCCCTGC<br>CATCTCCCAATATCAAACACCCCTATTTGTGTGATCCGTCCTAATTACCGCAGTACTACTCCTTCT<br>ATCCCTGCCCGTTCTTGCCGCCGGCATCACAATACTTCTAACAGACCGAAACCTAAACACAACC<br>TTCTTTGACCCTGCCGGAGGAGGAGACCCCATCCTATACCAACACTTA
KC789552	*O. niloticus*	TTGAGCCGGAATAGTAGGAACTGCACTAAGCCTCCTAATTCGGGCAGAACTAAGCCAGCCCGG<br>CTCTCTTCTCGGAGACGACCAAATCTATAATGTAATTGTTACAGCACATGCTTTCGTAATAATTTT<br>CTTTATAGTAATACCAATTATGATTGGAGGCTTTGGAAACTGACTAGTACCCCTCATGATTGGTG<br>CACCAGACATGGCCTTCCCTCGAATAAATAACATGAGCTTTTGACTTCTCCCCCCCTCATTTCTT<br>CTTCTTCTCGCCTCATCTGGAGTCGAAGCAGGTGCCGGCACAGGATGGACTGTTTATCCCCCGC<br>TCGCAGGCAATCTTGCCCACGCTGGACCTTCTGTTGACTTAACCATCTTCTCCCTCCACTTGGC<br>CGGAGTGTCATCTATTTTAGGTGCAATTAATTTTATCACAACCATTATTAACATGAAACCCCCTGC<br>CATCTCCCAATATCAAACACCCCTATTTGTGTGATCCGTCCTAATTACCGCAGTACTACTCCTTCT<br>ATCCCTGCCCGTTCTTGCCGCCGGCATCACAATACTTCTAACAGACCGAAACCTAAACACAACC<br>TTCTTTGACCCTGCCGGAGGAGGAGACCCCATCCTATACCAACACTTA
KJ553787	*O. aureus*	TTGAGCCGGAATAGTAGGAACCGCGCTAAGCCTCCTAATTCGGGCAGAACTAAGCCAACCCGG<br>CTCTCTCCTCGGAGACGACCAGATTTATAATGTAATTGTTACAGCACATGCTTTTGTAATAATTTT<br>CTTTATAGTAATGCCAATTATGATTGGAGGCTTTGGAAACTGACTAGTACCACTCATGATTGGTG<br>CCCCAGATATGGCCTTCCCTCGAATGAACAACATGAGTTTCTGACTCCTCCCTCCCTCATTCCTC<br>CTCCTCCTCGCCTCATCTGGAGTCGAAGCAGGTGCCGGCACAGGGTGAACTGTTTACCCCCCG<br>CTCGCAGGCAATCTTGCCCATGCTGGGCCTTCTGTCGACTTAACCATCTTCTCTCTCCACTTGGC<br>CGGGGTGTCATCTATTCTAGGCGCAATTAATTTCATTACAACAATCATTAACATGAAACCCCCCG<br>CCATCTCTCAATATCAAACACCCCTATTTGTATGGTCCGTTCTAATTACCGCAGTATTACTTCTTC<br>TATCCCTACCCGTTCTTGCCGCCGGCATCACAATACTTCTCACAGACCGAAACCTAAACACAAC<br>CTTCTTTGATCCTGCCGGAGGAGGAGACCCCATCCTTTACCAACACTTA
KJ553805	*O. aureus*	TTGAGCCGGAATAGTAGGAACCGCGCTAAGCCTCCTAATTCGGGCAGAACTAAGCCAACCCGG<br>CTCTCTCCTCGGAGACGACCAGATTTATAATGTAATTGTTACAGCACATGCTTTTGTAATAATTTT<br>CTTTATAGTAATGCCAATTATGATTGGAGGCTTTGGAAACTGACTAGTACCACTCATGATTGGTG<br>CCCCAGATATGGCCTTCCCTCGAATGAACAACATGAGTTTCTGACTCCTCCCTCCCTCATTCCTC<br>CTCCTCCTCGCCTCATCTGGAGTCGAAGCAGGTGCCGGCACAGGGTGAACTGTTTACCCCCCG<br>CTCGCAGGCAATCTTGCCCATGCTGGGCCTTCTGTCGACTTAACCATCTTCTCTCTCCACTTGGC<br>CGGGGTGTCATCTATTCTAGGCGCAATTAATTTCATTACAACAATCATTAACATGAAACCCCCCG<br>CCATCTCTCAATATCAAACACCCCTATTTGTATGGTCCGTTCTAATTACCGCAGTATTACTTCTTCT<br>ATCCCTACCCGTTCTTGCCGCCGGCATCACAATACTTCTCACAGACCGAAACCTAAACACAACC<br>TTCTTTGATCCTGCCGGAGGAGGAGACCCCATCCTTTACCAACACTTA
KM438537	*O. niloticus*	TTGAGCCGGAATAGTAGGAACTGCACTAAGCCTCCTAATTCGGGCAGAACTAAGCCAGCCCGG<br>CTCTCTTCTCGGAGACGACCAAATCTATAATGTAATTGTTACAGCACATGCTTTCGTAATAATTTT<br>CTTTATAGTAATACCAATTATGATTGGAGGCTTTGGAAACTGACTAGTACCCCTCATGATTGGTG<br>CACCGGACATGGCCTTCCCTCGAATAAATAACATGAGCTTTTGACTTCTCCCCCCCTCATTTCTT<br>CTTCTTCTCGCCTCATCTGGAGTCGAAGCAGGTGCCGGCACAGGATGGACTGTTTATCCCCCGC<br>TCGCAGGCAATCTTGCCCACGCTGGACCTTCTGTTGACTTAACCATCTTCTCCCTCCACTTGGC<br>CGGAGTGTCATCTATTTTAGGTGCAATTAATTTTATCACAACCATTATTAACATGAAACCCCCTGC<br>CATCTCCCAATATCAAACACCCCTATTTGTGTGATCCGTCCTAATTACCGCAGTACTACTCCTTCT<br>ATCCCTGCCCGTTCTTGCCGCCGGCATCACAATACTTCTAACAGACCGAAACCTAAACACAACC<br>TTCTTTGACCCTGCCGGAGGAGGAGACCCCATCCTATACCAACACTTA

KM438538	*O. niloticus*	TTGAGCCGGAATAGTAGGAACTGCACTAAGCCTCCTAATTCGGGCAGAACTAAGCCAGCCCGG<br>CTCTCTTCTCGGAGACGACCAAATCTATAATGTAATTGTTACAGCACATGCTTTCGTAATAATTTT<br>CTTTATAGTAATACCAATTATGATTGGAGGCTTTGGAAACTGACTAGTACCCCTCATGATTGGTG<br>CACCGGACATGGCCTTCCCTCGAATAAATAACATGAGCTTTTGACTTCTCCCCCCCTCATTTCTT<br>CTTCTTCTCGCCTCATCTGGAGTCGAAGCAGGTGCCGGCACAGGATGGACTGTTTATCCCCCGC<br>TCGCAGGCAATCTTGCCCACGCTGGACCTTCTGTTGACTTAACCATCTTCTCCCTCCACTTGGC<br>CGGAGTGTCATCTATTTTAGGTGCAATTAATTTTATCACAACCATTATTAACATGAAACCCCCTGC<br>CATCTCCCAATATCAAACACCCCTATTTGTGTGATCCGTCCTAATTACCGCAGTACTACTCCTTCT<br>ATCCCTGCCCGTTCTTGCCGCCGGCATCACAATACTTCTAACAGACCGAAACCTAAACACAACC<br>TTCTTTGACCCTGCCGGAGGAGGAGACCCCATCCTATACCAACACTTA
KM610786	*O. niloticus*	TTGAGCCGGAATAGTAGGAACTGCACTAAGCCTCCTAATTCGGGCAGAACTAAGCCAGCCCGG<br>CTCTCTTCTCGGAGACGACCAAATCTATAATGTAATTGTTACAGCACATGCTTTCGTAATAATTTT<br>CTTTATAGTAATACCAATTATGATTGGAGGCTTTGGAAACTGACTAGTACCCCTCATGATTGGTG<br>CACCAGACATGGCCTTCCCTCGAATAAATAACATGAGCTTTTGACTTCTCCCCCCCTCATTTCTT<br>CTTCTTCTCGCCTCATCTGGAGTCGAAGCAGGTGCCGGCACAGGATGGACTGTTTATCCCCCGC<br>TCGCAGGCAATCTTGCCCACGCTGGACCTTCTGTTGACTTAACCATCTTCTCCCTCCACTTGGC<br>CGGAGTGTCATCTATTTTAGGTGCAATTAATTTTATCACAACCATTATTAACATGAAACCCCCTGC<br>CATCTCCCAATATCAAACACCCCTATTTGTGTGATCCGTCCTAATTACCGCAGTACTACTCCTTCT<br>ATCCCTGCCCGTTCTTGCCGCCGGCATCACAATACTTCTAACAGACCGAAACCTAAACACAACC<br>TTCTTTGACCCTGCCGGAGGAGGAGACCCCATCCTATACCAACACTTA
KT307726	*O. niloticus*	TTGAGCCGGAATAGTAGGAACTGCATTAAGCCTCCTAATTCGGGCAGAACTAAGCCAGCCCGG<br>CTCTCTCCTCGGAGACGACCAGATTTATAATGTAATTGTTACAGCACATGCTTTCGTAATAATTTT<br>CTTTATAGTAATGCCAATTATAATTGGAGGTTTTGGAAACTGACTAGTGCCACTAATGATTGGTG<br>CACCAGACATGGCCTTCCCTCGAATAAATAACATGAGTTTTTGACTCCTCCCCCCCTCATTTCTC<br>CTTCTCCTCGCCTCATCCGGGGTCGAAGCAGGGGCCGGTACAGGATGGACTGTTTATCCCCCAC<br>TCGCAGGCAATCTCGCCCATGCTGGGCCTTCCGTTGACTTAACCATCTTCTCCCTCCACTTGGCC<br>GGGGTGTCATCTATTTTAGGTGCAATTAATTTTATTACAACCATTATTAACATAAAACCCCCTGCC<br>ATCTCCCAATATCAAACACCCCTCTTTGTATGATCCGTTCTAATTACCGCAGTACTACTCCTACTA<br>TCCCTACCCGTTCTTGCCGCCGGCATCACAATACTTCTAACAGACCGAAACCTAAACACAACCT<br>TCTTTGACCCTGCCGGAGGAGGAGACCCCATCCTTTACCAACACTTA
KT307727	*O. niloticus*	TTGAGCCGGAATAGTAGGAACTGCACTAAGCCTCCTAATTCGGGCAGAACTAAGCCAGCCCGG<br>CTCTCTTCTCGGAGACGACCAAATCTATAATGTAATTGTTACAGCACATGCTTTCGTAATAATTTT<br>CTTTATAGTAATACCAATTATGATTGGAGGCTTTGGAAACTGACTAGTACCCCTCATGATTGGTG<br>CACCAGACATGGCCTTCCCTCGAATAAATAACATGAGCTTTTGACTTCTCCCCCCCTCATTTCTT<br>CTTCTTCTCGCCTCATCTGGAGTCGAAGCAGGTGCCGGCACAGGATGGACTGTTTATCCCCCGC<br>TCGCAGGCAATCTTGCCCACGCTGGACCTTCTGTTGACTTAACCATCTTCTCCCTCCACTTGGC<br>CGGAGTGTCATCTATTTTAGGTGCAATTAATTTTATCACAACCATTATTAACATGAAACCCCCTGC<br>CATCTCCCAATATCAAACACCCCTATTTGTGTGATCCGTCCTAATTACCGCAGTACTACTCCTTCT<br>ATCCCTGCCCGTTCTTGCCGCCGGCATCACAATACTTCTAACAGACCGAAACCTAAACACAACC<br>TTCTTTGACCCTGCCGGAGGAGGAGACCCCATCCTATACCAACACTTA
KT307728	*O. niloticus*	TTGAGCCGGAATAGTAGGAACTGCACTAAGCCTCCTAATTCGGGCAGAACTAAGCCAGCCCGG<br>CTCTCTTCTCGGAGACGACCAAATCTATAATGTAATTGTTACAGCACATGCTTTCGTAATAATTTT<br>CTTTATAGTAATACCAATTATGATTGGAGGCTTTGGAAACTGACTAGTACCCCTCATGATTGGTG<br>CACCAGACATGGCCTTCCCTCGAATAAATAACATGAGCTTTTGACTTCTCCCCCCCTCATTTCTT<br>CTTCTTCTCGCCTCATCTGGAGTCGAAGCAGGTGCCGGCACAGGATGGACTGTTTATCCCCCGC<br>TCGCAGGCAATCTTGCCCACGCTGGACCTTCTGTTGACTTAACCATCTTCTCCCTCCACTTGGC<br>CGGAGTGTCATCTATTTTAGGTGCAATTAATTTTATCACAACCATTATTAACATGAAACCCCCTGC<br>CATCTCCCAATATCAAACACCCCTATTTGTGTGATCCGTCCTAATTACCGCAGTACTACTCCTTCT<br>ATCCCTGCCCGTTCTTGCCGCCGGCATCACAATACTTCTAACAGACCGAAACCTAAACACAACC<br>TTCTTTGACCCTGCCGGAGGAGGAGACCCCATCCTATACCAACACTTA
KT307729	*O. niloticus*	TTGAGCCGGAATAGTAGGAACTGCACTAAGCCTCCTAATTCGGGCAGAACTAAGCCAGCCCGG<br>CTCTCTTCTCGGAGACGACCAAATCTATAATGTAATTGTTACAGCACATGCTTTCGTAATAATTTT<br>CTTTATAGTAATACCAATTATGATTGGAGGCTTTGGAAACTGACTAGTACCCCTCATGATTGGTG<br>CACCAGACATGGCCTTCCCTCGAATAAATAACATGAGCTTTTGACTTCTCCCCCCCTCATTTCTT<br>CTTCTTCTCGCCTCATCTGGAGTCGAAGCAGGTGCCGGCACAGGATGGACTGTTTATCCCCCGC<br>TCGCAGGCAATCTTGCCCACGCTGGACCTTCTGTTGACTTAACCATCTTCTCCCTCCACTTGGC<br>CGGAGTGTCATCTATTTTAGGTGCAATTAATTTTATCACAACCATTATTAACATGAAACCCCCTGC<br>CATCTCCCAATATCAAACACCCCTATTTGTGTGATCCGTCCTAATTACCGCAGTACTACTCCTTCT<br>ATCCCTGCCCGTTCTTGCCGCCGGCATCACAATACTTCTAACAGACCGAAACCTAAACACAACC<br>TTCTTTGACCCTGCCGGAGGAGGAGACCCCATCCTATACCAACACTTA
KT307730	*O. niloticus*	TTGAGCCGGAATAGTAGGAACTGCACTAAGCCTCCTAATTCGGGCAGAACTAAGCCAGCCCGG<br>CTCTCTTCTCGGAGACGACCAAATCTATAATGTAATTGTTACAGCACATGCTTTCGTAATAATTTT<br>CTTTATAGTAATACCAATTATGATTGGAGGCTTTGGAAACTGACTAGTACCCCTCATGATTGGTG<br>CACCAGACATGGCCTTCCCTCGAATAAATAACATGAGCTTTTGACTTCTCCCCCCCTCATTTCTT<br>CTTCTTCTCGCCTCATCTGGAGTCGAAGCAGGTGCCGGCACAGGATGGACTGTTTATCCCCCGC<br>TCGCAGGCAATCTTGCCCACGCTGGACCTTCTGTTGACTTAACCATCTTCTCCCTCCACTTGGC<br>CGGAGTGTCATCTATTTTAGGTGCAATTAATTTTATCACAACCATTATTAACATGAAACCCCCTGC<br>CATCTCCCAATATCAAACACCCCTATTTGTGTGATCCGTCCTAATTACCGCAGTACTACTCCTTCT<br>ATCCCTGCCCGTTCTTGCCGCCGGCATCACAATACTTCTAACAGACCGAAACCTAAACACAACC<br>TTCTTTGACCCTGCCGGAGGAGGAGACCCCATCCTATACCAACACTTA

KT307731	*O. niloticus*	TTGAGCCGGAATAGTAGGAACTGCACTAAGCCTCCTAATTCGGGCAGAACTAAGCCAGCCCGG<br>CTCTCTTCTCGGAGACGACCAAATCTATAATGTAATTGTTACAGCACATGCTTTCGTAATAATTTT<br>CTTTATAGTAATACCAATTATGATTGGAGGCTTTGGAAACTGACTAGTACCCCTCATGATTGGTG<br>CACCAGACATGGCCTTCCCTCGAATAAATAACATGAGCTTTTGACTTCTCCCCCCCTCATTTCTT<br>CTTCTTCTCGCCTCATCTGGAGTCGAAGCAGGTGCCGGCACAGGATGGACTGTTTATCCCCCGC<br>TCGCAGGCAATCTTGCCCACGCTGGACCTTCTGTTGACTTAACCATCTTCTCCCTCCACTTGGC<br>CGGAGTGTCATCTATTTTAGGTGCAATTAATTTTATCACAACCATTATTAACATGAAACCCCCTGC<br>CATCTCCCAATATCAAACACCCCTATTTGTGTGATCCGTCCTAATTACCGCAGTACTACTCCTTCT<br>ATCCCTGCCCGTTCTTGCCGCCGGCATCACAATACTTCTAACAGACCGAAACCTAAACACAACC<br>TTCTTTGACCCTGCCGGAGGAGGAGACCCCATCCTATACCAACACTTA
KT307732	*O. niloticus*	TTGAGCCGGAATAGTAGGAACTGCAATAAGCCTCCTAATTCGGGCAGAACTAAGCCAGCCCGG<br>CTCTCTTCTCGGAGACGACCAAATCTATAATGTAATTGTTACAGCACATGCTTTCGTAATAATTTT<br>CTTTATAGTAATACCAATTATGATTGGAGGCTTTGGAAACTGACTAGTACCCCTCATGATTGGTG<br>CACCAGACATGGCCTTCCCTCGAATAAATAACATGAGCTTTTGACTTCTCCCCCCCTCATTTCTT<br>CTTCTTCTCGCCTCATCTGGAGTCGAAGCAGGTGCCGGCACAGGATGGACTGTTTATCCCCCGC<br>TCGCAGGCAATCTTGCCCACGCTGGACCTTCTGTTGACTTAACCATCTTCTCCCTCCACTTGGC<br>CGGAGTGTCATCTATTTTAGGTGCAATTAATTTTATCACAACCATTATTAACATGAAACCCCCTGC<br>CATCTCCCAATATCAAACACCCCTATTTGTGTGATCCGTCCTAATTACCGCAGTACTACTCCTTCT<br>ATCCCTGCCCGTTCTTGCCGCCGGCATCACAATACTTCTAACAGACCGAAACCTAAACACAACC<br>TTCTTTGACCCTGCCGGAGGAGGAGACCCCATCCTATACCAACACTTA
KT307733	*O. niloticus*	TTGAGCCGGAATAGTAGGAACTGCATTAAGCCTCCTAATTCGGGCAGAACTAAGCCAGCCCGG<br>CTCTCTCCTCGGAGACGACCAGATTTATAATGTAATTGTTACAGCACATGCTTTCGTAATAATTTT<br>CTTTATAGTAATGCCAATTATAATTGGAGGTTTTGGAAACTGACTAGTGCCACTAATGATTGGTG<br>CACCAGACATGGCCTTCCCTCGAATAAATAACATGAGTTTTTGACTCCTCCCCCCCTCATTTCTC<br>CTTCTCCTCGCCTCATCCGGGGTCGAAGCAGGGGCCGGTACAGGATGGACTGTTTATCCCCCAC<br>TCGCAGGCAATCTCGCCCATGCTGGGCCTTCCGTTGACTTAACCATCTTCTCCCTCCACTTGGCC<br>GGGGTGTCATCTATTTTAGGTGCAATTAATTTTATTACAACCATTATTAACATAAAACCCCCTGCC<br>ATCTCCCAATATCAAACACCCCTCTTTGTATGATCCGTTCTAATTACCGCAGTACTACTCCTACTA<br>TCCCTACCCGTTCTTGCCGCCGGCATCACAATACTTCTAACAGACCGAAACCTAAACACAACCT<br>TCTTTGACCCTGCCGGAGGAGGAGACCCCATCCTTTACCAACACTTA
KT307734	*O. niloticus*	TTGAGCCGGAATAGTAGGAACTGCACTAAGCCTCCTAATTCGGGCAGAACTAAGCCAGCCCGG<br>CTCTCTTCTCGGAGACGACCAAATCTATAATGTAATTGTTACAGCACATGCTTTCGTAATAATTTT<br>CTTTATAGTAATACCAATTATGATTGGAGGCTTTGGAAACTGACTAGTACCCCTCATGATTGGTG<br>CACCAGACATGGCCTTCCCTCGAATAAATAACATGAGCTTTTGACTTCTCCCCCCCTCATTTCTT<br>CTTCTTCTCGCCTCATCTGGAGTCGAAGCAGGTGCCGGCACAGGATGGACTGTTTATCCCCCGC<br>TCGCAGGCAATCTTGCCCACGCTGGACCTTCTGTTGACTTAACCATCTTCTCCCTCCACTTGGC<br>CGGAGTGTCATCTATTTTAGGTGCAATTAATTTTATCACAACCATTATTAACATGAAACCCCCTGC<br>CATCTCCCAATATCAAACACCCCTATTTGTGTGATCCGTCCTAATTACCGCAGTACTACTCCTTCT<br>ATCCCTGCCCGTTCTTGCCGCCGGCATCACAATACTTCTAACAGACCGAAACCTAAACACAACC<br>TTCTTTGACCCTGCCGGAGGAGGAGACCCCATCCTATACCAACACTTA
KT307735	*O. niloticus*	TTGAGCCGGAATAGTAGGAACTGCACTAAGCCTCCTAATTCGGGCAGAACTAAGCCAGCCCGG<br>CTCTCTTCTCGGAGACGACCAAATCTATAATGTAATTGTTACAGCACATGCTTTCGTAATAATTTT<br>CTTTATAGTAATACCAATTATGATTGGAGGCTTTGGAAACTGACTAGTACCCCTCATGATTGGTG<br>CACCAGACATGGCCTTCCCTCGAATAAATAACATGAGCTTTTGACTTCTCCCCCCCTCATTTCTT<br>CTTCTTCTCGCCTCATCTGGAGTCGAAGCAGGTGCCGGCACAGGATGGACTGTTTATCCCCCGC<br>TCGCAGGCAATCTTGCCCACGCTGGACCTTCTGTTGACTTAACCATCTTCTCCCTCCACTTGGC<br>CGGAGTGTCATCTATTTTAGGTGCAATTAATTTTATCACAACCATTATTAACATGAAACCCCCTGC<br>CATCTCCCAATATCAAACACCCCTATTTGTGTGATCCGTCCTAATTACCGCAGTACTACTCCTTCT<br>ATCCCTGCCCGTTCTTGCCGCCGGCATCACAATACTTCTAACAGACCGAAACCTAAACACAACC<br>TTCTTTGACCCTGCCGGAGGAGGAGACCCCATCCTATACCAACACTTA
KT307736	*O. niloticus*	TTGAGCCGGAATAGTAGGAACTGCACTAAGCCTCCTAATTCGGGCAGAACTAAGCCAGCCCGG<br>CTCTCTTCTCGGAGACGACCAAATCTATAATGTAATTGTTACAGCACATGCTTTCGTAATAATTTT<br>CTTTATAGTAATACCAATTATGATTGGAGGCTTTGGAAACTGACTAGTACCCCTCATGATTGGTG<br>CACCAGACATGGCCTTCCCTCGAATAAATAACATGAGCTTTTGACTTCTCCCCCCCTCATTTCTT<br>CTTCTTCTCGCCTCATCTGGAGTCGAAGCAGGTGCCGGCACAGGATGGACTGTTTATCCCCCGC<br>TCGCAGGCAATCTTGCCCACGCTGGACCTTCTGTTGACTTAACCATCTTCTCCCTCCACTTGGC<br>CGGAGTGTCATCTATTTTAGGTGCAATTAATTTTATCACAACCATTATTAACATGAAACCCCCTGC<br>CATCTCCCAATATCAAACACCCCTATTTGTGTGATCCGTCCTAATTACCGCAGTACTACTCCTTCT<br>ATCCCTGCCCGTTCTTGCCGCCGGCATCACAATACTTCTAACAGACCGAAACCTAAACACAACC<br>TTCTTTGACCCTGCCGGAGGAGGAGACCCCATCCTATACCAACACTTA
KT307737	*O. niloticus*	TTGAGCCGGAATAGTAGGAACTGCACTAAGCCTCCTAATTCGGGCAGAACTAAGCCAGCCCGG<br>CTCTCTTCTCGGAGACGACCAAATCTATAATGTAATTGTTACAGCACATGCTTTCGTAATAATTTT<br>CTTTATAGTAATACCAATTATGATTGGAGGCTTTGGAAACTGACTAGTACCCCTCATGATTGGTG<br>CACCAGACATGGCCTTCCCTCGAATAAATAACATGAGCTTTTGACTTCTCCCCCCCTCATTTCTT<br>CTTCTTCTCGCCTCATCTGGAGTCGAAGCAGGTGCCGGCACAGGATGGACTGTTTATCCCCCGC<br>TCGCAGGCAATCTTGCCCACGCTGGACCTTCTGTTGACTTAACCATCTTCTCCCTCCACTTGGC<br>CGGAGTGTCATCTATTTTAGGTGCAATTAATTTTATCACAACCATTATTAACATGAAACCCCCTGC<br>CATCTCCCAATATCAAACACCCCTATTTGTGTGATCCGTCCTAATTACCGCAGTACTACTCCTTCT<br>ATCCCTGCCCGTTCTTGCCGCCGGCATCACAATACTTCTAACAGACCGAAACCTAAACACAACC<br>TTCTTTGACCCTGCCGGAGGAGGAGACCCCATCCTATACCAACACTTA

KT307738	*O. niloticus*	TTGAGCCGGAATAGTAGGAACTGCACTAAGCCTCCTAATTCGGGCAGAACTAAGCCAGCCCGG<br>CTCTCTTCTCGGAGACGACCAAATCTATAATGTAATTGTTACAGCACATGCTTTCGTAATAATTTT<br>CTTTATAGTAATACCAATTATGATTGGAGGCTTTGGAAACTGACTAGTACCCCTCATGATTGGTG<br>CACCAGACATGGCCTTCCCTCGAATAAATAACATGAGCTTTTGACTTCTCCCCCCCTCATTTCTT<br>CTTCTTCTCGCCTCATCTGGAGTCGAAGCAGGTGCCGGCACAGGATGGACTGTTTATCCCCCGC<br>TCGCAGGCAATCTTGCCCACGCTGGACCTTCTGTTGACTTAACCATCTTCTCCCTCCACTTGGC<br>CGGAGTGTCATCTATTTTAGGTGCAATTAATTTTATCACAACCATTATTAACATGAAACCCCCTGC<br>CATCTCCCAATATCAAACACCCCTATTTGTGTGATCCGTCCTAATTACCGCAGTACTACTCCTTCT<br>ATCCCTGCCCGTTCTTGCCGCCGGCATCACAATACTTCTAACAGACCGAAACCTAAACACAACC<br>TTCTTTGACCCTGCCGGAGGAGGAGACCCCATCCTATACCAACACTTA
KT307739	*O. niloticus*	TGAAGCCGGAATAGTAGGAACTGCACTAAGCCTCCTAATTCGGGCAGAACTAAGCCAGCCCGG<br>CTCTCTTCTCGGAGACGACCAAATCTATAATGTAATTGTTACAGCACATGCTTTCGTAATAATTTT<br>CTTTATAGTAATACCAATTATGATTGGAGGCTTTGGAAACTGACTAGTACCCCTCATGATTGGTG<br>CACCAGACATGGCCTTCCCTCGAATAAATAACATGAGCTTTTGACTTCTCCCCCCCTCATTTCTT<br>CTTCTTCTCGCCTCATCTGGAGTCGAAGCAGGTGCCGGCACAGGATGGACTGTTTATCCCCCGC<br>TCGCAGGCAATCTTGCCCACGCTGGACCTTCTGTTGACTTAACCATCTTCTCCCTCCACTTGGC<br>CGGAGTGTCATCTATTTTAGGTGCAATTAATTTTATCACAACCATTATTAACATGAAACCCCCTGC<br>CATCTCCCAATATCAAACACCCCTATTTGTGTGATCCGTCCTAATTACCGCAGTACTACTCCTTCT<br>ATCCCTGCCCGTTCTTGCCGCCGGCATCACAATACTTCTAACAGACCGAAACCTAAACACAACC<br>TTCTTTGACCCTGCCGGAGGAGGAGACCCCATCCTATACCAACACTTA
KT307740	*O. niloticus*	TTGAGCCGGAATAGTAGGAACTGCACTAAGCCTCCTAATTCGGGCAGAACTAAGCCAGCCCGG<br>CTCTCTTCTCGGAGACGACCAAATCTATAATGTAATTGTTACAGCACATGCTTTCGTAATAATTTT<br>CTTTATAGTAATACCAATTATGATTGGAGGCTTTGGAAACTGACTAGTACCCCTCATGATTGGTG<br>CACCAGACATGGCCTTCCCTCGAATAAATAACATGAGCTTTTGACTTCTCCCCCCCTCATTTCTT<br>CTTCTTCTCGCCTCATCTGGAGTCGAAGCAGGTGCCGGCACAGGATGGACTGTTTATCCCCCGC<br>TCGCAGGCAATCTTGCCCACGCTGGACCTTCTGTTGACTTAACCATCTTCTCCCTCCACTTGGC<br>CGGAGTGTCATCTATTTTAGGTGCAATTAATTTTATCACAACCATTATTAACATGAAACCCCCTGC<br>CATCTCCCAATATCAAACACCCCTATTTGTGTGATCCGTCCTAATTACCGCAGTACTACTCCTTCT<br>ATCCCTGCCCGTTCTTGCCGCCGGCATCACAATACTTCTAACAGACCGAAACCTAAACACAACC<br>TTCTTTGACCCTGCCGGAGGAGGAGACCCCATCCTATACCAACACTTA
KT307741	*O. niloticus*	TTGAGCCGGAATAGTAGGAACCGCGCTAAGCCTCCTAATTCGGGCAGAACTAAGCCAGCCCGG<br>CTCTCTCCTCGGAGACGACCAGATTTATAATGTAATTGTTACAGCACATGCTTTTGTAATAATTTT<br>CTTTATAGTAATGCCAATTATGATTGGAGGCTTTGGAAACTGACTAGTACCACTCATGATTGGTG<br>CCCCAGATATGGCCTTCCCTCGAATGAACAACATGAGTTTCTGACTCCTCCCTCCCTCATTCCTC<br>CTCCTCCTCGCCTCATCTGGAGTCGAAGCAGGTGCCGGCACAGGGTGAACTGTTTACCCCCCG<br>CTCGCAGGCAATCTTGCCCATGCTGGGCCTTCTGTCGACTTAACCATCTTCTCCCTCCACTTGGC<br>CGGGGTGTCATCTATTCTAGGCGCAATTAATTTCATTACAACAATCATTAACATGAAACCCCCCG<br>CCATCTCTCAATATCAAACACCCCTATTTGTATGGTCCGTTCTAATTACCGCAGTATTACTTCTTC<br>TATCCCTACCCGTTCTTGCCGCCGGCATCACAATACTTCTCACAGACCGAAACCTAAACACAAC<br>CTTCTTTGATCCTGCCGGAGGAGGAGACCCCATCCTTTACCAACACTTA
KT307742	*O. niloticus*	TTGAGCCGGAATAGTAGGAACTGCATTAAGCCTCCTAATTCGGGCAGAACTAAGCCAGCCCGG<br>CTCTCTCCTCGGAGACGACCAGATTTATAATGTAATTGTTACAGCACATGCTTTCGTAATAATTTT<br>CTTTATAGTAATGCCAATTATAATTGGAGGTTTTGGAAACTGACTAGTGCCACTAATGATTGGTG<br>CACCAGACATGGCCTTCCCTCGAATAAATAACATGAGTTTTTGACTCCTCCCCCCCTCATTTCTC<br>CTTCTCCTCGCCTCATCCGGGGTCGAAGCAGGGGCCGGTACAGGATGGACTGTTTATCCCCCAC<br>TCGCAGGCAATCTCGCCCATGCTGGGCCTTCCGTTGACTTAACCATCTTCTCCCTCCACTTGGCC<br>GGGGTGTCATCTATTTTAGGTGCAATTAATTTTATTACAACCATTATTAACATAAAACCCCCTGCC<br>ATCTCCCAATATCAAACACCCCTCTTTGTATGATCCGTTCTAATTACCGCAGTACTACTCCTACTA<br>TCCCTACCCGTTCTTGCCGCCGGCATCACAATACTTCTAACAGACCGAAACCTAAACACAACCT<br>TCTTTGACCCTGCCGGAGGAGGAGACCCCATCCTTTACCAACACTTA
KT307743	*O. niloticus*	TTGAGCCGGAATAGTAGGAACTGCACTAAGCCTCCTAATTCGGGCAGAACTAAGCCAGCCCGG<br>CTCTCTTCTCGGAGACGACCAAATCTATAATGTAATTGTTACAGCACATGCTTTCGTAATAATTTT<br>CTTTATAGTAATACCAATTATGATTGGAGGCTTTGGAAACTGACTAGTACCCCTCATGATTGGTG<br>CACCAGACATGGCCTTCCCTCGAATAAATAACATGAGCTTTTGACTTCTCCCCCCCTCATTTCTT<br>CTTCTTCTCGCCTCATCTGGAGTCGAAGCAGGTGCCGGCACAGGATGGACTGTTTATCCCCCGC<br>TCGCAGGCAATCTTGCCCACGCTGGACCTTCTGTTGACTTAACCATCTTCTCCCTCCACTTGGC<br>CGGAGTGTCATCTATTTTAGGTGCAATTAATTTTATCACAACCATTATTAACATGAAACCCCCTGC<br>CATCTCCCAATATCAAACACCCCTATTTGTGTGATCCGTCCTAATTACCGCAGTACTACTCCTTCT<br>ATCCCTGCCCGTTCTTGCCGCCGGCATCACAATACTTCTAACAGACCGAAACCTAAACACAACC<br>TTCTTTGACCCTGCCGGAGGAGGAGACCCCATCCTATACCAACACTTA
KT307744	*O. niloticus*	TTGAGCCGGAATAGTAGGAACCGCGCTAAGCCTCCTAATTCGGGCAGAACTAAGCCAGCCCGG<br>CTCTCTCCTCGGAGACGACCAGATTTATAATGTAATTGTTACAGCACATGCTTTTGTAATAATTTT<br>CTTTATAGTAATGCCAATTATGATTGGAGGCTTTGGAAACTGACTAGTACCACTCATGATTGGTG<br>CCCCAGATATGGCCTTCCCTCGAATGAACAACATGAGTTTCTGACTCCTCCCTCCCTCATTCCTC<br>CTCCTCCTCGCCTCATCTGGAGTCGAAGCAGGTGCCGGCACAGGGTGAACTGTTTACCCCCCG<br>CTCGCAGGCAATCTTGCCCATGCTGGGCCTTCTGTCGACTTAACCATCTTCTCCCTCCACTTGGC<br>CGGGGTGTCATCTATTCTAGGCGCAATTAATTTCATTACAACAATCATTAACATGAAACCCCCCG<br>CCATCTCTCAATATCAAACACCCCTATTTGTATGGTCCGTTCTAATTACCGCAGTATTACTTCTTC<br>TATCCCTACCCGTTCTTGCCGCCGGCATCACAATACTTCTCACAGACCGAAACCTAAACACAAC<br>CTTCTTTGATCCTGCCGGAGGAGGAGACCCCATCCTTTACCAACACTTA

KT307745	*O. niloticus*	TTGAGCCGGAATAGTAGGAACTGCACTAAGCCTCCTAATTCGGGCAGAACTAAGCCAGCCCGG<br>CTCTCTTCTCGGAGACGACCAAATCTATAATGTAATTGTTACAGCACATGCTTTCGTAATAATTTT<br>CTTTATAGTAATACCAATTATGATTGGAGGCTTTGGAAACTGACTAGTACCCCTCATGATTGGTG<br>CACCAGACATGGCCTTCCCTCGAATAAATAACATGAGCTTTTGACTTCTCCCCCCCTCATTTCTT<br>CTTCTTCTCGCCTCATCTGGAGTCGAAGCAGGTGCCGGCACAGGATGGACTGTTTATCCCCCGC<br>TCGCAGGCAATCTTGCCCACGCTGGACCTTCTGTTGACTTAACCATCTTCTCCCTCCACTTGGC<br>CGGAGTGTCATCTATTTTAGGTGCAATTAATTTTATCACAACCATTATTAACATGAAACCCCCTGC<br>CATCTCCCAATATCAAACACCCCTATTTGTGTGATCCGTCCTAATTACCGCAGTACTACTCCTTCT<br>ATCCCTGCCCGTTCTTGCCGCCGGCATCACAATACTTCTAACAGACCGAAACCTAAACACAACC<br>TTCTTTGACCCTGCCGGAGGAGGAGACCCCATCCTATACCAACACTTA
KT307746	*O. niloticus*	TTGAGCCGGAATAGTAGGAACTGCACTAAGCCTCCTAATTCGGGCAGAACTAAGCCAGCCCGG<br>CTCTCTTCTCGGAGACGACCAAATCTATAATGTAATTGTTACAGCACATGCTTTCGTAATAATTTT<br>CTTTATAGTAATACCAATTATGATTGGAGGCTTTGGAAACTGACTAGTACCCCTCATGATTGGTG<br>CACCAGACATGGCCTTCCCTCGAATAAATAACATGAGCTTTTGACTTCTCCCCCCCTCATTTCTT<br>CTTCTTCTCGCCTCATCTGGAGTCGAAGCAGGTGCCGGCACAGGATGGACTGTTTATCCCCCGC<br>TCGCAGGCAATCTTGCCCACGCTGGACCTTCTGTTGACTTAACCATCTTCTCCCTCCACTTGGC<br>CGGAGTGTCATCTATTTTAGGTGCAATTAATTTTATCACAACCATTATTAACATGAAACCCCCTGC<br>CATCTCCCAATATCAAACACCCCTATTTGTGTGATCCGTCCTAATTACCGCAGTACTACTCCTTCT<br>ATCCCTGCCCGTTCTTGCCGCCGGCATCACAATACTTCTAACAGACCGAAACCTAAACACAACC<br>TTCTTTGACCCTGCCGGAGGAGGAGACCCCATCCTATACCAACACTTA
KT307747	*O. niloticus*	TTGAGCCGGAATAGTAGGAACTGCACTAAGCCTCCTAATTCGGGCAGAACTAAGCCAGCCCGG<br>CTCTCTTCTCGGAGACGACCAAATCTATAATGTAATTGTTACAGCACATGCTTTCGTAATAATTTT<br>CTTTATAGTAATACCAATTATGATTGGAGGCTTTGGAAACTGACTAGTACCCCTCATGATTGGTG<br>CACCAGACATGGCCTTCCCTCGAATAAATAACATGAGCTTTTGACTTCTCCCCCCCTCATTTCTT<br>CTTCTTCTCGCCTCATCTGGAGTCGAAGCAGGTGCCGGCACAGGATGGACTGTTTATCCCCCGC<br>TCGCAGGCAATCTTGCCCACGCTGGACCTTCTGTTGACTTAACCATCTTCTCCCTCCACTTGGC<br>CGGAGTGTCATCTATTTTAGGTGCAATTAATTTTATCACAACCATTATTAACATGAAACCCCCTGC<br>CATCTCCCAATATCAAACACCCCTATTTGTGTGATCCGTCCTAATTACCGCAGTACTACTCCTTCT<br>ATCCCTGCCCGTTCTTGCCGCCGGCATCACAATACTTCTAACAGACCGAAACCTAAACACAACC<br>TTCTTTGACCCTGCCGGAGGAGGAGACCCCATCCTATACCAACACTTA
KT307748	*O. niloticus*	TTGAGCCGGAATAGTAGGAACTGCATTAAGCCTCCTAATTCGGGCAGAACTAAGCCAGCCCGG<br>CTCTCTCCTCGGAGACGACCAGATTTATAATGTAATTGTTACAGCACATGCTTTCGTAATAATTTT<br>CTTTATAGTAATGCCAATTATAATTGGAGGTTTTGGAAACTGACTAGTGCCACTAATGATTGGTG<br>CACCAGACATGGCCTTCCCTCGAATAAATAACATGAGTTTTTGACTCCTCCCCCCCTCATTTCTC<br>CTTCTCCTCGCCTCATCCGGGGTCGAAGCAGGGGCCGGTACAGGATGGACTGTTTATCCCCCAC<br>TCGCAGGCAATCTCGCCCATGCTGGGCCTTCCGTTGACTTAACCATCTTCTCCCTCCACTTGGCC<br>GGGGTGTCATCTATTTTAGGTGCAATTAATTTTATTACAACCATTATTAACATAAAACCCCCTGCC<br>ATCTCCCAATATCAAACACCCCTCTTTGTATGATCCGTTCTAATTACCGCAGTACTACTCCTACTA<br>TCCCTACCCGTTCTTGCCGCCGGCATCACAATACTTCTAACAGACCGAAACCTAAACACAACCT<br>TCTTTGACCCTGCCGGAGGAGGAGACCCCATCCTTTACCAACACTTA
KT307749	*O. niloticus*	TTGAGCCGGAATAGTAGGAACCGCGCTAAGCCTCCTAATTCGGGCAGAACTAAGCCAGCCCGG<br>CTCTCTCCTCGGAGACGACCAGATTTATAATGTAATTGTTACAGCACATGCTTTTGTAATAATTTT<br>CTTTATAGTAATGCCAATTATGATTGGAGGCTTTGGAAACTGACTAGTACCACTCATGATTGGTG<br>CCCCAGATATGGCCTTCCCTCGAATGAACAACATGAGTTTCTGACTCCTCCCTCCCTCATTCCTC<br>CTCCTCCTCGCCTCATCTGGAGTCGAAGCAGGTGCCGGCACAGGGTGAACTGTTTACCCCCCG<br>CTCGCAGGCAATCTTGCCCATGCTGGGCCTTCTGTCGACTTAACCATCTTCTCCCTCCACTTGGC<br>CGGGGTGTCATCTATTCTAGGCGCAATTAATTTCATTACAACAATCATTAACATGAAACCCCCCG<br>CCATCTCTCAATATCAAACACCCCTATTTGTATGGTCCGTTCTAATTACCGCAGTATTACTTCTTC<br>TATCCCTACCCGTTCTTGCCGCCGGCATCACAATACTTCTCACAGACCGAAACCTAAACACAAC<br>CTTCTTTGATCCTGCCGGAGGAGGAGACCCCATCCTTTACCAACACTTA
KT307750	*O. niloticus*	TTGAGCCGGAATAGTAGGAACTGCACTAAGCCTCCTAATTCGGGCAGAACTAAGCCAGCCCGG<br>CTCTCTTCTCGGAGACGACCAAATCTATAATGTAATTGTTACAGCACATGCTTTCGTAATAATTTT<br>CTTTATAGTAATACCAATTATGATTGGAGGCTTTGGAAACTGACTAGTACCCCTCATGATTGGTG<br>CACCAGACATGGCCTTCCCTCGAATAAATAACATGAGCTTTTGACTTCTCCCCCCCTCATTTCTT<br>CTTCTTCTCGCCTCATCTGGAGTCGAAGCAGGTGCCGGCACAGGATGGACTGTTTATCCCCCGC<br>TCGCAGGCAATCTTGCCCACGCTGGACCTTCTGTTGACTTAACCATCTTCTCCCTCCACTTGGC<br>CGGAGTGTCATCTATTTTAGGTGCAATTAATTTTATCACAACCATTATTAACATGAAACCCCCTGC<br>CATCTCCCAATATCAAACACCCCTATTTGTGTGATCCGTCCTAATTACCGCAGTACTACTCCTTCT<br>ATCCCTGCCCGTTCTTGCCGCCGGCATCACAATACTTCTAACAGACCGAAACCTAAACACAACC<br>TTCTTTGACCCTGCCGGAGGAGGAGACCCCATCCTATACCAACACTTA
KT307751	*O. niloticus*	TTGAGCCGGAATAGTAGGAACTGCATTAAGCCTCCTAATTCGGGCAGAACTAAGCCAGCCCGG<br>CTCTCTCCTCGGAGACGACCAGATTTATAATGTAATTGTTACAGCACATGCTTTCGTAATAATTTT<br>CTTTATAGTAATGCCAATTATAATTGGAGGTTTTGGAAACTGACTAGTGCCACTAATGATTGGTG<br>CACCAGACATGGCCTTCCCTCGAATAAATAACATGAGTTTTTGACTCCTCCCCCCCTCATTTCTC<br>CTTCTCCTCGCCTCATCCGGGGTCGAAGCAGGGGCCGGTACAGGATGGACTGTTTATCCCCCAC<br>TCGCAGGCAATCTCGCCCATGCTGGGCCTTCCGTTGACTTAACCATCTTCTCCCTCCACTTGGCC<br>GGGGTGTCATCTATTTTAGGTGCAATTAATTTTATTACAACCATTATTAACATAAAACCCCCTGCC<br>ATCTCCCAATATCAAACACCCCTCTTTGTATGATCCGTTCTAATTACCGCAGTACTACTCCTACTA<br>TCCCTACCCGTTCTTGCCGCCGGCATCACAATACTTCTAACAGACCGAAACCTAAACACAACCT<br>TCTTTGACCCTGCCGGAGGAGGAGACCCCATCCTTTACCAACACTTA

KT307752	*O. niloticus*	TTGAGCCGGAATAGTAGGAACTGCATTAAGCCTCCTAATTCGGGCAGAACTAAGCCAGCCCGG<br>CTCTCTCCTCGGAGACGACCAGATTTATAATGTAATTGTTACAGCACATGCTTTCGTAATAATTTT<br>CTTTATAGTAATGCCAATTATAATTGGAGGTTTTGGAAACTGACTAGTGCCACTAATGATTGGTG<br>CACCAGACATGGCCTTCCCTCGAATAAATAACATGAGTTTTTGACTCCTCCCCCCCTCATTTCTC<br>CTTCTCCTCGCCTCATCCGGGGTCGAAGCAGGGGCCGGTACAGGATGGACTGTTTATCCCCCAC<br>TCGCAGGCAATCTCGCCCATGCTGGGCCTTCCGTTGACTTAACCATCTTCTCCCTCCACTTGGCC<br>GGGGTGTCATCTATTTTAGGTGCAATTAATTTTATTACAACCATTATTAACATAAAACCCCCTGCC<br>ATCTCCCAATATCAAACACCCCTCTTTGTATGATCCGTTCTAATTACCGCAGTACTACTCCTACTA<br>TCCCTACCCGTTCTTGCCGCCGGCATCACAATACTTCTAACAGACCGAAACCTAAACACAACCT<br>TCTTTGACCCTGCCGGAGGAGGAGACCCCATCCTTTACCAACACTTA
KT307753	*O. niloticus*	TTGAGCCGGAATAGTAGGAACTGCATTAAGCCTCCTAATTCGGGCAGAACTAAGCCAGCCCGG<br>CTCTCTCCTCGGAGACGACCAGATTTATAATGTAATTGTTACAGCACATGCTTTCGTAATAATTTT<br>CTTTATAGTAATGCCAATTATAATTGGAGGTTTTGGAAACTGACTAGTGCCACTAATGATTGGTG<br>CACCAGACATGGCCTTCCCTCGAATAAATAACATGAGTTTTTGACTCCTCCCCCCCTCATTTCTC<br>CTTCTCCTCGCCTCATCCGGGGTCGAAGCAGGGGCCGGTACAGGATGGACTGTTTATCCCCCAC<br>TCGCAGGCAATCTCGCCCATGCTGGGCCTTCCGTTGACTTAACCATCTTCTCCCTCCACTTGGCC<br>GGGGTGTCATCTATTTTAGGTGCAATTAATTTTATTACAACCATTATTAACATAAAACCCCCTGCC<br>ATCTCCCAATATCAAACACCCCTCTTTGTATGATCCGTTCTAATTACCGCAGTACTACTCCTACTA<br>TCCCTACCCGTTCTTGCCGCCGGCATCACAATACTTCTAACAGACCGAAACCTAAACACAACCT<br>TCTTTGACCCTGCCGGAGGAGGAGACCCCATCCTTTACCAACACTTA
KT307754	*O. niloticus*	TTGAGCCGGAATAGTAGGAACTGCACTAAGCCTCCTAATTCGGGCAGAACTAAGCCAGCCCGG<br>CTCTCTTCTCGGAGACGACCAAATCTATAATGTAATTGTTACAGCACATGCTTTCGTAATAATTTT<br>CTTTATAGTAATACCAATTATGATTGGAGGCTTTGGAAACTGACTAGTACCCCTCATGATTGGTG<br>CACCAGACATGGCCTTCCCTCGAATAAATAACATGAGCTTTTGACTTCTCCCCCCCTCATTTCTT<br>CTTCTTCTCGCCTCATCTGGAGTCGAAGCAGGTGCCGGCACAGGATGGACTGTTTATCCCCCGC<br>TCGCAGGCAATCTTGCCCACGCTGGACCTTCTGTTGACTTAACCATCTTCTCCCTCCACTTGGC<br>CGGAGTGTCATCTATTTTAGGTGCAATTAATTTTATCACAACCATTATTAACATGAAACCCCCTGC<br>CATCTCCCAATATCAAACACCCCTATTTGTGTGATCCGTCCTAATTACCGCAGTACTACTCCTTCT<br>ATCCCTGCCCGTTCTTGCCGCCGGCATCACAATACTTCTAACAGACCGAAACCTAAACACAACC<br>TTCTTTGACCCTGCCGGAGGAGGAGACCCCATCCTATACCAACACTTA
KT307755	*O. niloticus*	TTGAGCCGGAATAGTAGGAACCGCGCTAAGCCTCCTAATTCGGGCAGAACTAAGCCAGCCCGG<br>CTCTCTCCTCGGAGACGACCAGATTTATAATGTAATTGTTACAGCACATGCTTTTGTAATAATTTT<br>CTTTATAGTAATGCCAATTATGATTGGAGGCTTTGGAAACTGACTAGTACCACTCATGATTGGTG<br>CCCCAGATATGGCCTTCCCTCGAATGAACAACATGAGTTTCTGACTCCTCCCTCCCTCATTCCTC<br>CTCCTCCTCGCCTCATCTGGAGTCGAAGCAGGTGCCGGCACAGGGTGAACTGTTTACCCCCCG<br>CTCGCAGGCAATCTTGCCCATGCTGGGCCTTCTGTCGACTTAACCATCTTCTCCCTCCACTTGGC<br>CGGGGTGTCATCTATTCTAGGCGCAATTAATTTCATTACAACAATCATTAACATGAAACCCCCCG<br>CCATCTCTCAATATCAAACACCCCTATTTGTATGGTCCGTTCTAATTACCGCAGTATTACTTCTTC<br>TATCCCTACCCGTTCTTGCCGCCGGCATCACAATACTTCTCACAGACCGAAACCTAAACACAAC<br>CTTCTTTGATCCTGCCGGAGGAGGAGACCCCATCCTTTACCAACACTTA
KT307756	*O. niloticus*	TTGAGCCGGAATAGTAGGAACTGCACTAAGCCTCCTAATTCGGGCAGAACTAAGCCAGCCCGG<br>CTCTCTTCTCGGAGACGACCAAATCTATAATGTAATTGTTACAGCACATGCTTTCGTAATAATTTT<br>CTTTATAGTAATACCAATTATGATTGGAGGCTTTGGAAACTGACTAGTACCCCTCATGATTGGTG<br>CACCAGACATGGCCTTCCCTCGAATAAATAACATGAGCTTTTGACTTCTCCCCCCCTCATTTCTT<br>CTTCTTCTCGCCTCATCTGGAGTCGAAGCAGGTGCCGGCACAGGATGGACTGTTTATCCCCCGC<br>TCGCAGGCAATCTTGCCCACGCTGGACCTTCTGTTGACTTAACCATCTTCTCCCTCCACTTGGC<br>CGGAGTGTCATCTATTTTAGGTGCAATTAATTTTATCACAACCATTATTAACATGAAACCCCCTGC<br>CATCTCCCAATATCAAACACCCCTATTTGTGTGATCCGTCCTAATTACCGCAGTACTACTCCTTCT<br>ATCCCTGCCCGTTCTTGCCGCCGGCATCACAATACTTCTAACAGACCGAAACCTAAACACAACC<br>TTCTTTGACCCTGCCGGAGGAGGAGACCCCATCCTATACCAACACTTA
KT307757	*O. niloticus*	TTGAGCCGGAATAGTAGGAACTGCACTAAGCCTCCTAATTCGGGCAGAACTAAGCCAGCCCGG<br>CTCTCTTCTCGGAGACGACCAAATCTATAATGTAATTGTTACAGCACATGCTTTCGTAATAATTTT<br>CTTTATAGTAATACCAATTATGATTGGAGGCTTTGGAAACTGACTAGTACCCCTCATGATTGGTG<br>CACCAGACATGGCCTTCCCTCGAATAAATAACATGAGCTTTTGACTTCTCCCCCCCTCATTTCTT<br>CTTCTTCTCGCCTCATCTGGAGTCGAAGCAGGTGCCGGCACAGGATGGACTGTTTATCCCCCGC<br>TCGCAGGCAATCTTGCCCACGCTGGACCTTCTGTTGACTTAACCATCTTCTCCCTCCACTTGGC<br>CGGAGTGTCATCTATTTTAGGTGCAATTAATTTTATCACAACCATTATTAACATGAAACCCCCTGC<br>CATCTCCCAATATCAAACACCCCTATTTGTGTGATCCGTCCTAATTACCGCAGTACTACTCCTTCT<br>ATCCCTGCCCGTTCTTGCCGCCGGCATCACAATACTTCTAACAGACCGAAACCTAAACACAACC<br>TTCTTTGACCCTGCCGGAGGAGGAGACCCCATCCTATACCAACACTTA
KT307758	*O. niloticus*	TTGAGCCGGAATAGTAGGAACTGCACTAAGCCTCCTAATTCGGGCAGAACTAAGCCAGCCCGG<br>CTCTCTTCTCGGAGACGACCAAATCTATAATGTAATTGTTACAGCACATGCTTTCGTAATAATTTT<br>CTTTATAGTAATACCAATTATGATTGGAGGCTTTGGAAACTGACTAGTACCCCTCATGATTGGTG<br>CACCAGACATGGCCTTCCCTCGAATAAATAACATGAGCTTTTGACTTCTCCCCCCCTCATTTCTT<br>CTTCTTCTCGCCTCATCTGGAGTCGAAGCAGGTGCCGGCACAGGATGGACTGTTTATCCCCCGC<br>TCGCAGGCAATCTTGCCCACGCTGGACCTTCTGTTGACTTAACCATCTTCTCCCTCCACTTGGC<br>CGGAGTGTCATCTATTTTAGGTGCAATTAATTTTATCACAACCATTATTAACATGAAACCCCCTGC<br>CATCTCCCAATATCAAACACCCCTATTTGTGTGATCCGTCCTAATTACCGCAGTACTACTCCTTCT<br>ATCCCTGCCCGTTCTTGCCGCCGGCATCACAATACTTCTAACAGACCGAAACCTAAACACAACC<br>TTCTTTGACCCTGCCGGAGGAGGAGACCCCATCCTATACCAACACTTA

KT307759	*O. niloticus*	TTGAGCCGGAATAGTAGGAACCGCGCTAAGCCTCCTAATTCGGGCAGAACTAAGCCAGCCCGG<br>CTCTCTCCTCGGAGACGACCAGATTTATAATGTAATTGTTACAGCACATGCTTTTGTAATAATTTT<br>CTTTATAGTAATGCCAATTATGATTGGAGGCTTTGGAAACTGACTAGTACCACTCATGATTGGTG<br>CCCCAGATATGGCCTTCCCTCGAATGAACAACATGAGTTTCTGACTCCTCCCTCCCTCATTCCTC<br>CTCCTCCTCGCCTCATCTGGAGTCGAAGCAGGTGCCGGCACAGGGTGAACTGTTTACCCCCCG<br>CTCGCAGGCAATCTTGCCCATGCTGGGCCTTCTGTCGACTTAACCATCTTCTCCCTCCACTTGGC<br>CGGGGTGTCATCTATTCTAGGCGCAATTAATTTCATTACAACAATCATTAACATGAAACCCCCCG<br>CCATCTCTCAATATCAAACACCCCTATTTGTATGGTCCGTTCTAATTACCGCAGTATTACTTCTTC<br>TATCCCTACCCGTTCTTGCCGCCGGCATCACAATACTTCTCACAGACCGAAACCTAAACACAAC<br>CTTCTTTGATCCTGCCGGAGGAGGAGACCCCATCCTTTACCAACACTTA
KT307760	*O. niloticus*	TTGAGCCGGAATAGTAGGAACTGCACTAAGCCTCCTAATTCGGGCAGAACTAAGCCAGCCCGG<br>CTCTCTTCTCGGAGACGACCAAATCTATAATGTAATTGTTACAGCACATGCTTTCGTAATAATTTT<br>CTTTATAGTAATACCAATTATGATTGGAGGCTTTGGAAACTGACTAGTACCCCTCATGATTGGTG<br>CACCAGACATGGCCTTCCCTCGAATAAATAACATGAGCTTTTGACTTCTCCCCCCCTCATTTCTT<br>CTTCTTCTCGCCTCATCTGGAGTCGAAGCAGGTGCCGGCACAGGATGGACTGTTTATCCCCCGC<br>TCGCAGGCAATCTTGCCCACGCTGGACCTTCTGTTGACTTAACCATCTTCTCCCTCCACTTGGC<br>CGGAGTGTCATCTATTTTAGGTGCAATTAATTTTATCACAACCATTATTAACATGAAACCCCCTGC<br>CATCTCCCAATATCAAACACCCCTATTTGTGTGATCCGTCCTAATTACCGCAGTACTACTCCTTCT<br>ATCCCTGCCCGTTCTTGCCGCCGGCATCACAATACTTCTAACAGACCGAAACCTAAACACAACC<br>TTCTTTGACCCTGCCGGAGGAGGAGACCCCATCCTATACCAACACTTA
KT307761	*O. niloticus*	TTGAGCCGGAATAGTAGGAACCGCGCTAAGCCTCCTAATTCGGGCAGAACTAAGCCAGCCCGG<br>CTCTCTCCTCGGAGACGACCAGATTTATAATGTAATTGTTACAGCACATGCTTTTGTAATAATTTT<br>CTTTATAGTAATGCCAATTATGATTGGAGGCTTTGGAAACTGACTAGTACCACTCATGATTGGTG<br>CCCCAGATATGGCCTTCCCTCGAATGAACAACATGAGTTTCTGACTCCTCCCTCCCTCATTCCTC<br>CTCCTCCTCGCCTCATCTGGAGTCGAAGCAGGTGCCGGCACAGGGTGAACTGTTTACCCCCCG<br>CTCGCAGGCAATCTTGCCCATGCTGGGCCTTCTGTCGACTTAACCATCTTCTCCCTCCACTTGGC<br>CGGGGTGTCATCTATTCTAGGCGCAATTAATTTCATTACAACAATCATTAACATGAAACCCCCCG<br>CCATCTCTCAATATCAAACACCCCTATTTGTATGGTCCGTTCTAATTACCGCAGTATTACTTCTTC<br>TATCCCTACCCGTTCTTGCCGCCGGCATCACAATACTTCTCACAGACCGAAACCTAAACACAAC<br>CTTCTTTGATCCTGCCGGAGGAGGAGACCCCATCCTTTACCAACACTTA
KT307762	*O. niloticus*	TTGAGCCGGAATAGTAGGAACTGCATTAAGCCTCCTAATTCGGGCAGAACTAAGCCAGCCCGG<br>CTCTCTCCTCGGAGACGACCAGATTTATAATGTAATTGTTACAGCACATGCTTTCGTAATAATTTT<br>CTTTATAGTAATGCCAATTATAATTGGAGGTTTTGGAAACTGACTAGTGCCACTAATGATTGGTG<br>CACCAGACATGGCCTTCCCTCGAATAAATAACATGAGTTTTTGACTCCTCCCCCCCTCATTTCTC<br>CTTCTCCTCGCCTCATCCGGGGTCGAAGCAGGGGCCGGTACAGGATGGACTGTTTATCCCCCAC<br>TCGCAGGCAATCTCGCCCATGCTGGGCCTTCCGTTGACTTAACCATCTTCTCCCTCCACTTGGCC<br>GGGGTGTCATCTATTTTAGGTGCAATTAATTTTATTACAACCATTATTAACATAAAACCCCCTGCC<br>ATCTCCCAATATCAAACACCCCTCTTTGTATGATCCGTTCTAATTACCGCAGTACTACTCCTACTA<br>TCCCTACCCGTTCTTGCCGCCGGCATCACAATACTTCTAACAGACCGAAACCTAAACACAACCT<br>TCTTTGACCCTGCCGGAGGAGGAGACCCCATCCTTTACCAACACTTA
KT307763	*O. niloticus*	TTGAGCCGGAATAGTAGGAACTGCACTAAGCCTCCTAATTCGGGCAGAACTAAGCCAGCCCGG<br>CTCTCTTCTCGGAGACGACCAAATCTATAATGTAATTGTTACAGCACATGCTTTCGTAATAATTTT<br>CTTTATAGTAATACCAATTATGATTGGAGGCTTTGGAAACTGACTAGTACCCCTCATGATTGGTG<br>CACCAGACATGGCCTTCCCTCGAATAAATAACATGAGCTTTTGACTTCTCCCCCCCTCATTTCTT<br>CTTCTTCTCGCCTCATCTGGAGTCGAAGCAGGTGCCGGCACAGGATGGACTGTTTATCCCCCGC<br>TCGCAGGCAATCTTGCCCACGCTGGACCTTCTGTTGACTTAACCATCTTCTCCCTCCACTTGGC<br>CGGAGTGTCATCTATTTTAGGTGCAATTAATTTTATCACAACCATTATTAACATGAAACCCCCTGC<br>CATCTCCCAATATCAAACACCCCTATTTGTGTGATCCGTCCTAATTACCGCAGTACTACTCCTTCT<br>ATCCCTGCCCGTTCTTGCCGCCGGCATCACAATACTTCTAACAGACCGAAACCTAAACACAACC<br>TTCTTTGACCCTGCCGGAGGAGGAGACCCCATCCTATACCAACACTTA
KT307764	*O. niloticus*	TTGAGCCGGAATAGTAGGAACTGCACTAAGCCTCCTAATTCGGGCAGAACTAAGCCAGCCCGG<br>CTCTCTTCTCGGAGACGACCAAATCTATAATGTAATTGTTACAGCACATGCTTTCGTAATAATTTT<br>CTTTATAGTAATACCAATTATGATTGGAGGCTTTGGAAACTGACTAGTACCCCTCATGATTGGTG<br>CACCAGACATGGCCTTCCCTCGAATAAATAACATGAGCTTTTGACTTCTCCCCCCCTCATTTCTT<br>CTTCTTCTCGCCTCATCTGGAGTCGAAGCAGGTGCCGGCACAGGATGGACTGTTTATCCCCCGC<br>TCGCAGGCAATCTTGCCCACGCTGGACCTTCTGTTGACTTAACCATCTTCTCCCTCCACTTGGC<br>CGGAGTGTCATCTATTTTAGGTGCAATTAATTTTATCACAACCATTATTAACATGAAACCCCCTGC<br>CATCTCCCAATATCAAACACCCCTATTTGTGTGATCCGTCCTAATTACCGCAGTACTACTCCTTCT<br>ATCCCTGCCCGTTCTTGCCGCCGGCATCACAATACTTCTAACAGACCGAAACCTAAACACAACC<br>TTCTTTGACCCTGCCGGAGGAGGAGACCCCATCCTATACCAACACTTA
KT307765	*O. niloticus*	TTGAGCCGGAATAGTAGGAACTGCACTAAGTCTCCTTATTCGGGCAGAACTAAGCCAGCCCGG<br>CTCTCTCCTCGGAGACGACCAGATTTATAATGTAATTGTTACAGCACATGCTTTCGTAATAATTTT<br>CTTTATAGTAATACCAATTATAATTGGAGGGTTTGGAAACTGACTGGTGCCACTTATGATTGGAG<br>CACCAGACATGGCCTTCCCTCGAATAAATAACATGAGTTTTTGACTCCTTCCCCCCTCATTTCTC<br>CTTCTCCTCGCCTCATCCGGAGTCGAAGCAGGAGCCGGTACAGGATGAACTGTTTATCCTCCCC<br>TCGCAGGCAATCTCGCCCACGCTGGACCTTCTGTTGACTTGACCATCTTTTCCCTCCACTTGGC<br>CGGGGTGTCATCTATTTTAGGCGCAATTAATTTTATCACAACCATTATTAACATAAAACCCCCTGC<br>CATCTCTCAATATCAAACACCCCTCTTTGTGTGATCCGTTCTAATTACCGCAGTATTACTCCTACT<br>ATCCCTGCCCGTTCTTGCCGCCGGCATCACAATACTTCTAACAGACCGAAACCTAAACACAACC<br>TTCTTTGACCCTGCCGGAGGAGGAGACCCCATCCTTTACCAACACTTA

KT307766	*O. niloticus*	TTGAGCCGGAATAGTAGGAACTGCACTAAGTCTCCTTATTCGGGCAGAACTAAGCCAGCCCGG<br>CTCTCTCCTCGGAGACGACCAGATTTATAATGTAATTGTTACAGCACATGCTTTCGTAATAATTTT<br>CTTTATAGTAATACCAATTATAATTGGAGGGTTTGGAAACTGACTGGTGCCACTTATGATTGGAG<br>CACCAGACATGGCCTTCCCTCGAATAAATAACATGAGTTTTTGACTCCTTCCCCCCTCATTTCTC<br>CTTCTCCTCGCCTCATCCGGAGTCGAAGCAGGAGCCGGTACAGGATGAACTGTTTATCCTCCCC<br>TCGCAGGCAATCTCGCCCACGCTGGACCTTCTGTTGACTTGACCATCTTTTCCCTCCACTTGGC<br>CGGGGTGTCATCTATTTTAGGCGCAATTAATTTTATCACAACCATTATTAACATAAAACCCCCTGC<br>CATCTCTCAATATCAAACACCCCTCTTTGTGTGATCCGTTCTAATTACCGCAGTATTACTCCTACT<br>ATCCCTGCCCGTTCTTGCCGCCGGCATCACAATACTTCTAACAGACCGAAACCTAAACACAACC<br>TTCTTTGACCCTGCCGGAGGAGGAGACCCCATCCTTTACCAACACTTA
KT307767	*O. niloticus*	TTGAGCCGGAATAGTAGGAACTGCACTAAGCCTCCTAATTCGGGCAGAACTAAGCCAGCCCGG<br>CTCTCTTCTCGGAGACGACCAAATCTATAATGTAATTGTTACAGCACATGCTTTCGTAATAATTTT<br>CTTTATAGTAATACCAATTATGATTGGAGGCTTTGGAAACTGACTAGTACCCCTCATGATTGGTG<br>CACCAGACATGGCCTTCCCTCGAATAAATAACATGAGCTTTTGACTTCTCCCCCCCTCATTTCTT<br>CTTCTTCTCGCCTCATCTGGAGTCGAAGCAGGTGCCGGCACAGGATGGACTGTTTATCCCCCGC<br>TCGCAGGCAATCTTGCCCACGCTGGACCTTCTGTTGACTTAACCATCTTCTCCCTCCACTTGGC<br>CGGAGTGTCATCTATTTTAGGTGCAATTAATTTTATCACAACCATTATTAACATGAAACCCCCTGC<br>CATCTCCCAATATCAAACACCCCTATTTGTGTGATCCGTCCTAATTACCGCAGTACTACTCCTTCT<br>ATCCCTGCCCGTTCTTGCCGCCGGCATCACAATACTTCTAACAGACCGAAACCTAAACACAACC<br>TTCTTTGACCCTGCCGGAGGAGGAGACCCCATCCTATACCAACACTTA
KT307768	*O. niloticus*	TTGAGCCGGAATAGTAGGAACTGCACTAAGCCTCCTAATTCGGGCAGAACTAAGCCAGCCCGG<br>CTCTCTTCTCGGAGACGACCAAATCTATAATGTAATTGTTACAGCACATGCTTTCGTAATAATTTT<br>CTTTATAGTAATACCAATTATGATTGGAGGCTTTGGAAACTGACTAGTACCCCTCATGATTGGTG<br>CACCAGACATGGCCTTCCCTCGAATAAATAACATGAGCTTTTGACTTCTCCCCCCCTCATTTCTT<br>CTTCTTCTCGCCTCATCTGGAGTCGAAGCAGGTGCCGGCACAGGATGGACTGTTTATCCCCCGC<br>TCGCAGGCAATCTTGCCCACGCTGGACCTTCTGTTGACTTAACCATCTTCTCCCTCCACTTGGC<br>CGGAGTGTCATCTATTTTAGGTGCAATTAATTTTATCACAACCATTATTAACATGAAACCCCCTGC<br>CATCTCCCAATATCAAACACCCCTATTTGTGTGATCCGTCCTAATTACCGCAGTACTACTCCTTCT<br>ATCCCTGCCCGTTCTTGCCGCCGGCATCACAATACTTCTAACAGACCGAAACCTAAACACAACC<br>TTCTTTGACCCTGCCGGAGGAGGAGACCCCATCCTATACCAACACTTA
KT307769	*O. niloticus*	TTGAGCCGGAATAGTAGGAACCGCGCTAAGCCTCCTAATTCGGGCAGAACTAAGCCAGCCCGG<br>CTCTCTCCTCGGAGACGACCAGATTTATAATGTAATTGTTACAGCACATGCTTTTGTAATAATTTT<br>CTTTATAGTAATGCCAATTATGATTGGAGGCTTTGGAAACTGACTAGTACCACTCATGATTGGTG<br>CCCCAGATATGGCCTTCCCTCGAATGAACAACATGAGTTTCTGACTCCTCCCTCCCTCATTCCTC<br>CTCCTCCTCGCCTCATCTGGAGTCGAAGCAGGTGCCGGCACAGGGTGAACTGTTTACCCCCCG<br>CTCGCAGGCAATCTTGCCCATGCTGGGCCTTCTGTCGACTTAACCATCTTCTCCCTCCACTTGGC<br>CGGGGTGTCATCTATTCTAGGCGCAATTAATTTCATTACAACAATCATTAACATGAAACCCCCCG<br>CCATCTCTCAATATCAAACACCCCTATTTGTATGGTCCGTTCTAATTACCGCAGTATTACTTCTTC<br>TATCCCTACCCGTTCTTGCCGCCGGCATCACAATACTTCTCACAGACCGAAACCTAAACACAAC<br>CTTCTTTGATCCTGCCGGAGGAGGAGACCCCATCCTTTACCAACACTTA
KT307770	*O. niloticus*	TTGAGCCGGAATAGTAGGAACTGCACTAAGCCTCCTAATTCGGGCAGAACTAAGCCAGCCCGG<br>CTCTCTTCTCGGAGACGACCAAATCTATAATGTAATTGTTACAGCACATGCTTTCGTAATAATTTT<br>CTTTATAGTAATACCAATTATGATTGGAGGCTTTGGAAACTGACTAGTACCCCTCATGATTGGTG<br>CACCAGACATGGCCTTCCCTCGAATAAATAACATGAGCTTTTGACTTCTCCCCCCCTCATTTCTT<br>CTTCTTCTCGCCTCATCTGGAGTCGAAGCAGGTGCCGGCACAGGATGGACTGTTTATCCCCCGC<br>TCGCAGGCAATCTTGCCCACGCTGGACCTTCTGTTGACTTAACCATCTTCTCCCTCCACTTGGC<br>CGGAGTGTCATCTATTTTAGGTGCAATTAATTTTATCACAACCATTATTAACATGAAACCCCCTGC<br>CATCTCCCAATATCAAACACCCCTATTTGTGTGATCCGTCCTAATTACCGCAGTACTACTCCTTCT<br>ATCCCTGCCCGTTCTTGCCGCCGGCATCACAATACTTCTAACAGACCGAAACCTAAACACAACC<br>TTCTTTGACCCTGCCGGAGGAGGAGACCCCATCCTATACCAACACTTA
KT307771	*O. niloticus*	TTGAGCCGGAATAGTAGGAACTGCACTAAGCCTCCTAATTCGGGCAGAACTAAGCCAGCCCGG<br>CTCTCTTCTCGGAGACGACCAAATCTATAATGTAATTGTTACAGCACATGCTTTCGTAATAATTTT<br>CTTTATAGTAATACCAATTATGATTGGAGGCTTTGGAAACTGACTAGTACCCCTCATGATTGGTG<br>CACCAGACATGGCCTTCCCTCGAATAAATAACATGAGCTTTTGACTTCTCCCCCCCTCATTTCTT<br>CTTCTTCTCGCCTCATCTGGAGTCGAAGCAGGTGCCGGCACAGGATGGACTGTTTATCCCCCGC<br>TCGCAGGCAATCTTGCCCACGCTGGACCTTCTGTTGACTTAACCATCTTCTCCCTCCACTTGGC<br>CGGAGTGTCATCTATTTTAGGTGCAATTAATTTTATCACAACCATTATTAACATGAAACCCCCTGC<br>CATCTCCCAATATCAAACACCCCTATTTGTGTGATCCGTCCTAATTACCGCAGTACTACTCCTTCT<br>ATCCCTGCCCGTTCTTGCCGCCGGCATCACAATACTTCTAACAGACCGAAACCTAAACACAACC<br>TTCTTTGACCCTGCCGGAGGAGGAGACCCCATCCTATACCAACACTTA
KT307772	*O. niloticus*	TTGAGCCGGAATAGTAGGAACTGCATTAAGCCTCCTAATTCGGGCAGAACTAAGCCAGCCCGG<br>CTCTCTCCTCGGAGACGACCAGATTTATAATGTAATTGTTACAGCACATGCTTTCGTAATAATTTT<br>CTTTATAGTAATGCCAATTATAATTGGAGGTTTTGGAAACTGACTAGTGCCACTAATGATTGGTG<br>CACCAGACATGGCCTTCCCTCGAATAAATAACATGAGTTTTTGACTCCTCCCCCCCTCATTTCTC<br>CTTCTCCTCGCCTCATCCGGGGTCGAAGCAGGGGCCGGTACAGGATGGACTGTTTATCCCCCAC<br>TCGCAGGCAATCTCGCCCATGCTGGGCCTTCCGTTGACTTAACCATCTTCTCCCTCCACTTGGCC<br>GGGGTGTCATCTATTTTAGGTGCAATTAATTTTATTACAACCATTATTAACATAAAACCCCCTGCC<br>ATCTCCCAATATCAAACACCCCTCTTTGTATGATCCGTTCTAATTACCGCAGTACTACTCCTACTA<br>TCCCTACCCGTTCTTGCCGCCGGCATCACAATACTTCTAACAGACCGAAACCTAAACACAACCT<br>TCTTTG\ACCCTGCCGGAGGAGGAGACCCCATCCTTTACCAACACTTA

KT307773	*O. niloticus*	TTGAGCCGGAATAGTAGGAACTGCACTAAGCCTCCTAATTCGGGCAGAACTAAGCCAGCCCGG<br>CTCTCTTCTCGGAGACGACCAAATCTATAATGTAATTGTTACAGCACATGCTTTCGTAATAATTTT<br>CTTTATAGTAATACCAATTATGATTGGAGGCTTTGGAAACTGACTAGTACCCCTCATGATTGGTG<br>CACCAGACATGGCCTTCCCTCGAATAAATAACATGAGCTTTTGACTTCTCCCCCCCTCATTTCTT<br>CTTCTTCTCGCCTCATCTGGAGTCGAAGCAGGTGCCGGCACAGGATGGACTGTTTATCCCCCGC<br>TCGCAGGCAATCTTGCCCACGCTGGACCTTCTGTTGACTTAACCATCTTCTCCCTCCACTTGGC<br>CGGAGTGTCATCTATTTTAGGTGCAATTAATTTTATCACAACCATTATTAACATGAAACCCCCTGC<br>CATCTCCCAATATCAAACACCCCTATTTGTGTGATCCGTCCTAATTACCGCAGTACTACTCCTTCT<br>ATCCCTGCCCGTTCTTGCCGCCGGCATCACAATACTTCTAACAGACCGAAACCTAAACACAACC<br>TTCTTTGACCCTGCCGGAGGAGGAGACCCCATCCTATACCAACACTTA
KT307774	*O. niloticus*	TTGAGCCGGAATAGTAGGAACTGCATTAAGCCTCCTAATTCGGGCAGAACTAAGCCAGCCCGG<br>CTCTCTCCTCGGAGACGACCAGATTTATAATGTAATTGTTACAGCACATGCTTTCGTAATAATTTT<br>CTTTATAGTAATGCCAATTATAATTGGAGGTTTTGGAAACTGACTAGTGCCACTAATGATTGGTG<br>CACCAGACATGGCCTTCCCTCGAATAAATAACATGAGTTTTTGACTCCTCCCCCCCTCATTTCTC<br>CTTCTCCTCGCCTCATCCGGGGTCGAAGCAGGGGCCGGTACAGGATGGACTGTTTATCCCCCAC<br>TCGCAGGCAATCTCGCCCATGCTGGGCCTTCCGTTGACTTAACCATCTTCTCCCTCCACTTGGCC<br>GGGGTGTCATCTATTTTAGGTGCAATTAATTTTATTACAACCATTATTAACATAAAACCCCCTGCC<br>ATCTCCCAATATCAAACACCCCTCTTTGTATGATCCGTTCTAATTACCGCAGTACTACTCCTACTA<br>TCCCTACCCGTTCTTGCCGCCGGCATCACAATACTTCTAACAGACCGAAACCTAAACACAACCT<br>TCTTTGACCCTGCCGGAGGAGGAGACCCCATCCTTTACCAACACTTA
KT307775	*O. niloticus*	TTGAGCCGGAATAGTAGGAACCGCGCTAAGCCTCCTAATTCGGGCAGAACTAAGCCAGCCCGG<br>CTCTCTCCTCGGAGACGACCAGATTTATAATGTAATTGTTACAGCACATGCTTTTGTAATAATTTT<br>CTTTATAGTAATGCCAATTATGATTGGAGGCTTTGGAAACTGACTAGTACCACTCATGATTGGTG<br>CCCCAGATATGGCCTTCCCTCGAATGAACAACATGAGTTTCTGACTCCTCCCTCCCTCATTCCTC<br>CTCCTCCTCGCCTCATCTGGAGTCGAAGCAGGTGCCGGCACAGGGTGAACTGTTTACCCCCCG<br>CTCGCAGGCAATCTTGCCCATGCTGGGCCTTCTGTCGACTTAACCATCTTCTCCCTCCACTTGGC<br>CGGGGTGTCATCTATTCTAGGCGCAATTAATTTCATTACAACAATCATTAACATGAAACCCCCCG<br>CCATCTCTCAATATCAAACACCCCTATTTGTATGGTCCGTTCTAATTACCGCAGTATTACTTCTTC<br>TATCCCTACCCGTTCTTGCCGCCGGCATCACAATACTTCTCACAGACCGAAACCTAAACACAAC<br>CTTCTTTGATCCTGCCGGAGGAGGAGACCCCATCCTTTACCAACACTTA
KT307776	*O. niloticus*	TTGAGCCGGAATAGTAGGAACCGCGCTAAGCCTCCTAATTCGGGCAGAACTAAGCCAGCCCGG<br>CTCTCTCCTCGGAGACGACCAGATTTATAATGTAATTGTTACAGCACATGCTTTTGTAATAATTTT<br>CTTTATAGTAATGCCAATTATGATTGGAGGCTTTGGAAACTGACTAGTACCACTCATGATTGGTG<br>CCCCAGATATGGCCTTCCCTCGAATGAACAACATGAGTTTCTGACTCCTCCCTCCCTCATTCCTC<br>CTCCTCCTCGCCTCATCTGGAGTCGAAGCAGGTGCCGGCACAGGGTGAACTGTTTACCCCCCG<br>CTCGCAGGCAATCTTGCCCATGCTGGGCCTTCTGTCGACTTAACCATCTTCTCCCTCCACTTGGC<br>CGGGGTGTCATCTATTCTAGGCGCAATTAATTTCATTACAACAATCATTAACATGAAACCCCCCG<br>CCATCTCTCAATATCAAACACCCCTATTTGTATGGTCCGTTCTAATTACCGCAGTATTACTTCTTC<br>TATCCCTACCCGTTCTTGCCGCCGGCATCACAATACTTCTCACAGACCGAAACCTAAACACAAC<br>CTTCTTTGATCCTGCCGGAGGAGGAGACCCCATCCTTTACCAACACTTA
KT307777	*O. niloticus*	TTGAGCCGGAATAGTAGGAACTGCACTAAGCCTCCTAATTCGGGCAGAACTAAGCCAGCCCGG<br>CTCTCTTCTCGGAGACGACCAAATCTATAATGTAATTGTTACAGCACATGCTTTCGTAATAATTTT<br>CTTTATAGTAATACCAATTATGATTGGAGGCTTTGGAAACTGACTAGTACCCCTCATGATTGGTG<br>CACCAGACATGGCCTTCCCTCGAATAAATAACATGAGCTTTTGACTTCTCCCCCCCTCATTTCTT<br>CTTCTTCTCGCCTCATCTGGAGTCGAAGCAGGTGCCGGCACAGGATGGACTGTTTATCCCCCGC<br>TCGCAGGCAATCTTGCCCACGCTGGACCTTCTGTTGACTTAACCATCTTCTCCCTCCACTTGGC<br>CGGAGTGTCATCTATTTTAGGTGCAATTAATTTTATCACAACCATTATTAACATGAAACCCCCTGC<br>CATCTCCCAATATCAAACACCCCTATTTGTGTGATCCGTCCTAATTACCGCAGTACTACTCCTTCT<br>ATCCCTGCCCGTTCTTGCCGCCGGCATCACAATACTTCTAACAGACCGAAACCTAAACACAACC<br>TTCTTTGACCCTGCCGGAGGAGGAGACCCCATCCTATACCAACACTTA
KT307778	*O. niloticus*	TTGAGCCGGAATAGTAGGAACTGCACTAAGCCTCCTAATTCGGGCAGAACTAAGCCAGCCCGG<br>CTCTCTTCTCGGAGACGACCAAATCTATAATGTAATTGTTACAGCACATGCTTTCGTAATAATTTT<br>CTTTATAGTAATACCAATTATGATTGGAGGCTTTGGAAACTGACTAGTACCCCTCATGATTGGTG<br>CACCAGACATGGCCTTCCCTCGAATAAATAACATGAGCTTTTGACTTCTCCCCCCCTCATTTCTT<br>CTTCTTCTCGCCTCATCTGGAGTCGAAGCAGGTGCCGGCACAGGATGGACTGTTTATCCCCCGC<br>TCGCAGGCAATCTTGCCCACGCTGGACCTTCTGTTGACTTAACCATCTTCTCCCTCCACTTGGC<br>CGGAGTGTCATCTATTTTAGGTGCAATTAATTTTATCACAACCATTATTAACATGAAACCCCCTGC<br>CATCTCCCAATATCAAACACCCCTATTTGTGTGATCCGTCCTAATTACCGCAGTACTACTCCTTCT<br>ATCCCTGCCCGTTCTTGCCGCCGGCATCACAATACTTCTAACAGACCGAAACCTAAACACAACC<br>TTCTTTGACCCTGCCGGAGGAGGAGACCCCATCCTATACCAACACTTA
KT307779	*O. niloticus*	TTGAGCCGGAATAGTAGGAACTGCACTAAGCCTCCTAATTCGGGCAGAACTAAGCCAGCCCGG<br>CTCTCTTCTCGGAGACGACCAAATCTATAATGTAATTGTTACAGCACATGCTTTCGTAATAATTTT<br>CTTTATAGTAATACCAATTATGATTGGAGGCTTTGGAAACTGACTAGTACCCCTCATGATTGGTG<br>CACCAGACATGGCCTTCCCTCGAATAAATAACATGAGCTTTTGACTTCTCCCCCCCTCATTTCTT<br>CTTCTTCTCGCCTCATCTGGAGTCGAAGCAGGTGCCGGCACAGGATGGACTGTTTATCCCCCGC<br>TCGCAGGCAATCTTGCCCACGCTGGACCTTCTGTTGACTTAACCATCTTCTCCCTCCACTTGGC<br>CGGAGTGTCATCTATTTTAGGTGCAATTAATTTTATCACAACCATTATTAACATGAAACCCCCTGC<br>CATCTCCCAATATCAAACACCCCTATTTGTGTGATCCGTCCTAATTACCGCAGTACTACTCCTTCT<br>ATCCCTGCCCGTTCTTGCCGCCGGCATCACAATACTTCTAACAGACCGAAACCTAAACACAACC<br>TTCTTTGACCCTGCCGGAGGAGGAGACCCCATCCTATACCAACACTTA

KT307780	*O. niloticus*	TTGAGCCGGAATAGTAGGAACTGCACTAAGCCTCCTAATTCGGGCAGAACTAAGCCAGCCCGG<br>CTCTCTTCTCGGAGACGACCAAATCTATAATGTAATTGTTACAGCACATGCTTTCGTAATAATTTT<br>CTTTATAGTAATACCAATTATGATTGGAGGCTTTGGAAACTGACTAGTACCCCTCATGATTGGTG<br>CACCAGACATGGCCTTCCCTCGAATAAATAACATGAGCTTTTGACTTCTCCCCCCCTCATTTCTT<br>CTTCTTCTCGCCTCATCTGGAGTCGAAGCAGGTGCCGGCACAGGATGGACTGTTTATCCCCCGC<br>TCGCAGGCAATCTTGCCCACGCTGGACCTTCTGTTGACTTAACCATCTTCTCCCTCCACTTGGC<br>CGGAGTGTCATCTATTTTAGGTGCAATTAATTTTATCACAACCATTATTAACATGAAACCCCCTGC<br>CATCTCCCAATATCAAACACCCCTATTTGTGTGATCCGTCCTAATTACCGCAGTACTACTCCTTCT<br>ATCCCTGCCCGTTCTTGCCGCCGGCATCACAATACTTCTAACAGACCGAAACCTAAACACAACC<br>TTCTTTGACCCTGCCGGAGGAGGAGACCCCATCCTATACCAACACTTA
KT307781	*O. niloticus*	TTGAGCCGGAATAGTAGGAACTGCACTAAGCCTCCTAATTCGGGCAGAACTAAGCCAGCCCGG<br>CTCTCTTCTCGGAGACGACCAAATCTATAATGTAATTGTTACAGCACATGCTTTCGTAATAATTTT<br>CTTTATAGTAATACCAATTATGATTGGAGGCTTTGGAAACTGACTAGTACCCCTCATGATTGGTG<br>CACCAGACATGGCCTTCCCTCGAATAAATAACATGAGCTTTTGACTTCTCCCCCCCTCATTTCTT<br>CTTCTTCTCGCCTCATCTGGAGTCGAAGCAGGTGCCGGCACAGGATGGACTGTTTATCCCCCGC<br>TCGCAGGCAATCTTGCCCACGCTGGACCTTCTGTTGACTTAACCATCTTCTCCCTCCACTTGGC<br>CGGAGTGTCATCTATTTTAGGTGCAATTAATTTTATCACAACCATTATTAACATGAAACCCCCTGC<br>CATCTCCCAATATCAAACACCCCTATTTGTGTGATCCGTCCTAATTACCGCAGTACTACTCCTTCT<br>ATCCCTGCCCGTTCTTGCCGCCGGCATCACAATACTTCTAACAGACCGAAACCTAAACACAACC<br>TTCTTTGACCCTGCCGGAGGAGGAGACCCCATCCTATACCAACACTTA
KT307782	*O. niloticus*	TTGAGCCGGAATAGTAGGAACTGCACTAAGCCTCCTAATTCGGGCAGAACTAAGCCAGCCCGG<br>CTCTCTTCTCGGAGACGACCAAATCTATAATGTAATTGTTACAGCACATGCTTTCGTAATAATTTT<br>CTTTATAGTAATACCAATTATGATTGGAGGCTTTGGAAACTGACTAGTACCCCTCATGATTGGTG<br>CACCAGACATGGCCTTCCCTCGAATAAATAACATGAGCTTTTGACTTCTCCCCCCCTCATTTCTT<br>CTTCTTCTCGCCTCATCTGGAGTCGAAGCAGGTGCCGGCACAGGATGGACTGTTTATCCCCCGC<br>TCGCAGGCAATCTTGCCCACGCTGGACCTTCTGTTGACTTAACCATCTTCTCCCTCCACTTGGC<br>CGGAGTGTCATCTATTTTAGGTGCAATTAATTTTATCACAACCATTATTAACATGAAACCCCCTGC<br>CATCTCCCAATATCAAACACCCCTATTTGTGTGATCCGTCCTAATTACCGCAGTACTACTCCTTCT<br>ATCCCTGCCCGTTCTTGCCGCCGGCATCACAATACTTCTAACAGACCGAAACCTAAACACAACC<br>TTCTTTGACCCTGCCGGAGGAGGAGACCCCATCCTATACCAACACTTA
KT307783	*O. niloticus*	TTGAGCCGGAATAGTAGGAACTGCATTAAGCCTCCTAATTCGGGCAGAACTAAGCCAGCCCGG<br>CTCTCTCCTCGGAGACGACCAGATTTATAATGTAATTGTTACAGCACATGCTTTCGTAATAATTTT<br>CTTTATAGTAATGCCAATTATAATTGGAGGTTTTGGAAACTGACTAGTGCCACTAATGATTGGTG<br>CACCAGACATGGCCTTCCCTCGAATAAATAACATGAGTTTTTGACTCCTCCCCCCCTCATTTCTC<br>CTTCTCCTCGCCTCATCCGGGGTCGAAGCAGGGGCCGGTACAGGATGGACTGTTTATCCCCCAC<br>TCGCAGGCAATCTCGCCCATGCTGGGCCTTCCGTTGACTTAACCATCTTCTCCCTCCACTTGGCC<br>GGGGTGTCATCTATTTTAGGTGCAATTAATTTTATTACAACCATTATTAACATAAAACCCCCTGCC<br>ATCTCCCAATATCAAACACCCCTCTTTGTATGATCCGTTCTAATTACCGCAGTACTACTCCTACTA<br>TCCCTACCCGTTCTTGCCGCCGGCATCACAATACTTCTAACAGACCGAAACCTAAACACAACCT<br>TCTTTGACCCTGCCGGAGGAGGAGACCCCATCCTTTACCAACACTTA
KT307784	*O. niloticus*	TTGAGCCGGAATAGTAGGAACTGCACTAAGCCTCCTAATTCGGGCAGAACTAAGCCAGCCCGG<br>CTCTCTTCTCGGAGACGACCAAATCTATAATGTAATTGTTACAGCACATGCTTTCGTAATAATTTT<br>CTTTATAGTAATACCAATTATGATTGGAGGCTTTGGAAACTGACTAGTACCCCTCATGATTGGTG<br>CACCAGACATGGCCTTCCCTCGAATAAATAACATGAGCTTTTGACTTCTCCCCCCCTCATTTCTT<br>CTTCTTCTCGCCTCATCTGGAGTCGAAGCAGGTGCCGGCACAGGATGGACTGTTTATCCCCCGC<br>TCGCAGGCAATCTTGCCCACGCTGGACCTTCTGTTGACTTAACCATCTTCTCCCTCCACTTGGC<br>CGGAGTGTCATCTATTTTAGGTGCAATTAATTTTATCACAACCATTATTAACATGAAACCCCCTGC<br>CATCTCCCAATATCAAACACCCCTATTTGTGTGATCCGTCCTAATTACCGCAGTACTACTCCTTCT<br>ATCCCTGCCCGTTCTTGCCGCCGGCATCACAATACTTCTAACAGACCGAAACCTAAACACAACC<br>TTCTTTGACCCTGCCGGAGGAGGAGACCCCATCCTATACCAACACTTA
KT307785	*O. niloticus*	TTGAGCCGGAATAGTAGGAACTGCACTAAGCCTCCTAATTCGGGCAGAACTAAGCCAGCCCGG<br>CTCTCTTCTCGGAGACGACCAAATCTATAATGTAATTGTTACAGCACATGCTTTCGTAATAATTTT<br>CTTTATAGTAATACCAATTATGATTGGAGGCTTTGGAAACTGACTAGTACCCCTCATGATTGGTG<br>CACCAGACATGGCCTTCCCTCGAATAAATAACATGAGCTTTTGACTTCTCCCCCCCTCATTTCTT<br>CTTCTTCTCGCCTCATCTGGAGTCGAAGCAGGTGCCGGCACAGGATGGACTGTTTATCCCCCGC<br>TCGCAGGCAATCTTGCCCACGCTGGACCTTCTGTTGACTTAACCATCTTCTCCCTCCACTTGGC<br>CGGAGTGTCATCTATTTTAGGTGCAATTAATTTTATCACAACCATTATTAACATGAAACCCCCTGC<br>CATCTCCCAATATCAAACACCCCTATTTGTGTGATCCGTCCTAATTACCGCAGTACTACTCCTTCT<br>ATCCCTGCCCGTTCTTGCCGCCGGCATCACAATACTTCTAACAGACCGAAACCTAAACACAACC<br>TTCTTTGACCCTGCCGGAGGAGGAGACCCCATCCTATACCAACACTTA
KU565808	*O. niloticus*	TTGAGCCGGAATAGTAGGAACTGCACTAAGCCTCCTAATTCGGGCAGAACTAAGCCAGCCCGG<br>CTCTCTTCTCGGAGACGACCAAATCTATAATGTAATTGTTACAGCACATGCTTTCGTAATAATTTT<br>CTTTATAGTAATACCAATTATGATTGGAGGCTTTGGAAACTGACTAGTACCCCTCATGATTGGTG<br>CACCAGACATGGCCTTCCCTCGAATAAATAACATGAGCTTTTGACTTCTCCCCCCCTCATTTCTT<br>CTTCTTCTCGCCTCATCTGGAGTCGAAGCAGGTGCCGGCACAGGATGGACTGTTTATCCCCCGC<br>TCGCAGGCAATCTTGCCCACGCTGGACCTTCTGTTGACTTAACCATCTTCTCCCTCCACTTGGC<br>CGGAGTGTCATCTATTTTAGGTGCAATTAATTTTATCACAACCATTATTAACATGAAACCCCCTGC<br>CATCTCCCAATATCAAACACCCCTATTTGTGTGATCCGTCCTAATTACCGCAGTACTACTCCTTCT<br>ATCCCTGCCCGTTCTTGCCGCCGGCATCACAATACTTCTAACAGACCGAAACCTAAACACAACC<br>TTCTTTGACCCTGCCGGAGGAGGAGACCCCATCCTATACCAACACTTA

KU565810	*O. niloticus*	TTGAGCTGGAATAGTAGGAACTGCACTAAGCCTCCTAATTCGGGCAGAACTAAGCCAGCCCGG<br>CTCTCTTCTCGGAGACGACCAAATCTATAATGTAATTGTTACAGCACATGCTTTCGTAATAATTTT<br>CTTTATAGTAATACCAATTATGATTGGAGGCTTTGGAAACTGACTAGTACCCCTCATGATTGGTG<br>CACCAGACATGGCCTTCCCTCGAATAAATAACATGAGCTTTTGACTTCTCCCCCCCTCATTTCTT<br>CTTCTTCTCGCCTCATCTGGAGTCGAAGCAGGTGCCGGCACAGGATGGACTGTTTATCCCCCGC<br>TCGCAGGCAATCTTGCCCACGCTGGACCTTCTGTTGACTTAACCATCTTCTCCCTCCACTTGGC<br>CGGAGTGTCATCTATTTTAGGTGCAATTAATTTTATCACAACCATTATTAACATGAAACCCCCTGC<br>CATCTCCCAATATCAAACACCCCTATTTGTGTGATCCGTCCTAATTACCGCAGTACTACTCCTTCT<br>ATCCCTGCCCGTTCTTGCCGCCGGCATCACAATACTTCTAACAGACCGAAACCTAAACACAACC<br>TTCTTTGACCCTGCCGGAGGAGGAGACCCCATCCTATACCAACACTTA
KU565811	*O. niloticus*	TCGAGCTGGAATAGTAGGAACTGCACTAAGCCTCATAATTCGGGCAGAAATAAGCCAGCCCGG<br>CTCTCTTCTCGGAGACGACCAAATCTATAATGTAATTGTTACAGCACATGCTTTCGTAATAATTTT<br>CTTTATAGTAATACCAATTATGATTGGAGGCTTTGGAAACTGACTAGTACCCCTCATGATTGGTG<br>CACCAGACATGGCCTTCCCTCGAATAAATAACATGAGCTTTTGACTTCTCCCCCCCTCATTTCTT<br>CTTCTTCTCGCCTCATCTGGAGTCGAAGCAGGTGCCGGCACAGGATGGACTGTTTATCCCCCGC<br>TCGCAGGCAATCTTGCCCACGCTGGACCTTCTGTTGACTTAACCATCTTCTCCCTCCACTTGGC<br>CGGAGTGTCATCTATTTTAGGTGCAATTAATTTTATCACAACCATTATTAACATGAAACCCCCTGC<br>CATCTCCCAATATCAAACACCCCTATTTGTGTGATCCGTCCTAATTACCGCAGTACTACTCCTTCT<br>ATCCCTGCCCGTTCTTGCCGCCGGCATCACAATACTTCTAACAGACCGAAACCTAAACACAACC<br>TTCTTTGACCCTGCCGGAGGAGGAGACCCCATCCTATACCAACACTTA
KU565812	*O. niloticus*	TTGAGCCGGAATAGTAGGAACTGCACTAAGCCTCCTAATTCGGGCAGAACTAAGCCAGCCCGG<br>CTCTCTTCTCGGAGACGACCAAATCTATAATGTAATTGTTACAGCACATGCTTTCGTAATAATTTT<br>CTTTATAGTAATACCAATTATGATTGGAGGCTTTGGAAACTGACTAGTACCCCTCATGATTGGTG<br>CACCAGACATGGCCTTCCCTCGAATAAATAACATGAGCTTTTGACTTCTCCCCCCCTCATTTCTT<br>CTTCTTCTCGCCTCATCTGGAGTCGAAGCAGGTGCCGGCACAGGATGGACTGTTTATCCCCCGC<br>TCGCAGGCAATCTTGCCCACGCTGGACCTTCTGTTGACTTAACCATCTTCTCCCTCCACTTGGC<br>CGGAGTGTCATCTATTTTAGGTGCAATTAATTTTATCACAACCATTATTAACATGAAACCCCCTGC<br>CATCTCCCAATATCAAACACCCCTATTTGTGTGATCCGTCCTAATTACCGCAGTACTACTCCTTCT<br>ATCCCTGCCCGTTCTTGCCGCCGGCATCACAATACTTCTAACAGACCGAAACCTAAACACAACC<br>TTCTTTGACCCTGCCGGAGGAGGAGACCCCATCCTATACCAACACTTA
KU565813	*O. niloticus*	TTGAGCCGGAATAGTAGGAACTGCACTAAGCCTCCTAATTCGGGCAGAACTAAGCCAGCCCGG<br>CTCTCTTCTCGGAGACGACCAAATCTATAATGTAATTGTTACAGCACATGCTTTCGTAATAATTTT<br>CTTTATAGTAATACCAATTATGATTGGAGGCTTTGGAAACTGACTAGTACCCCTCATGATTGGTG<br>CACCAGACATGGCCTTCCCTCGAATAAATAACATGAGCTTTTGACTTCTCCCCCCCTCATTTCTT<br>CTTCTTCTCGCCTCATCTGGAGTCGAAGCAGGTGCCGGCACAGGATGGACTGTTTATCCCCCGC<br>TCGCAGGCAATCTTGCCCACGCTGGACCTTCTGTTGACTTAACCATCTTCTCCCTCCACTTGGC<br>CGGAGTGTCATCTATTTTAGGTGCAATTAATTTTATCACAACCATTATTAACATGAAACCCCCTGC<br>CATCTCCCAATATCAAACACCCCTATTTGTGTGATCCGTCCTAATTACCGCAGTACTACTCCTTCT<br>ATCCCTGCCCGTTCTTGCCGCCGGCATCACAATACTTCTAACAGACCGAAACCTAAACACAACC<br>TTCTTTGACCCTGCCGGAGGAGGAGACCCCATCCTATACCAACACTTA
KU565814	*O. niloticus*	TTGAGCCGGAATAGTAGGAACTGCACTAAGCCTCCTAATTCGGGCAGAACTAAGCCAGCCCGG<br>CTCTCTTCTCGGAGACGACCAAATCTATAATGTAATTGTTACAGCACATGCTTTCGTAATAATTTT<br>CTTTATAGTAATACCAATTATGATTGGAGGCTTTGGAAACTGACTAGTACCCCTCATGATTGGTG<br>CACCAGACATGGCCTTCCCTCGAATAAATAACATGAGCTTTTGACTTCTCCCCCCCTCATTTCTT<br>CTTCTTCTCGCCTCATCTGGAGTCGAAGCAGGTGCCGGCACAGGATGGACTGTTTATCCCCCGC<br>TCGCAGGCAATCTTGCCCACGCTGGACCTTCTGTTGACTTAACCATCTTCTCCCTCCACTTGGC<br>CGGAGTGTCATCTATTTTAGGTGCAATTAATTTTATCACAACCATTATTAACATGAAACCCCCTGC<br>CATCTCCCAATATCAAACACCCCTATTTGTGTGATCCGTCCTAATTACCGCAGTACTACTCCTTCT<br>ATCCCTGCCCGTTCTTGCCGCCGGCATCACAATACTTCTAACAGACCGAAACCTAAACACAACC<br>TTCTTTGACCCTGCCGGAGGAGGAGACCCCATCCTATACCAACACTTA
KU565814.1	*O. niloticus*	TTGAGCCGGAATAGTAGGAACTGCACTAAGCCTCCTAATTCGGGCAGAACTAAGCCAGCCCGG<br>CTCTCTTCTCGGAGACGACCAAATCTATAATGTAATTGTTACAGCACATGCTTTCGTAATAATTTT<br>CTTTATAGTAATACCAATTATGATTGGAGGCTTTGGAAACTGACTAGTACCCCTCATGATTGGTG<br>CACCAGACATGGCCTTCCCTCGAATAAATAACATGAGCTTTTGACTTCTCCCCCCCTCATTTCTT<br>CTTCTTCTCGCCTCATCTGGAGTCGAAGCAGGTGCCGGCACAGGATGGACTGTTTATCCCCCGC<br>TCGCAGGCAATCTTGCCCACGCTGGACCTTCTGTTGACTTAACCATCTTCTCCCTCCACTTGGC<br>CGGAGTGTCATCTATTTTAGGTGCAATTAATTTTATCACAACCATTATTAACATGAAACCCCCTGC<br>CATCTCCCAATATCAAACACCCCTATTTGTGTGATCCGTCCTAATTACCGCAGTACTACTCCTTCT<br>ATCCCTGCCCGTTCTTGCCGCCGGCATCACAATACTTCTAACAGACCGAAACCTAAACACAACC<br>TTCTTTGACCCTGCCGGAGGAGGAGACCCCATCCTATACCAACACTTA
KU565815	*O. niloticus*	TTGAGCCGGAATAGTAGGAACTGCAGTAAGCCTCCTAATTCGGGCAGAACTAAGCCAGCCCGG<br>CTCTCTTCTCGGAGACGACCAAATCTATAATGTAATTGTTACAGCACATGCTTTCGTAATAATTTT<br>CTTTATAGTAATACCAATTATGATTGGAGGCTTTGGAAACTGACTAGTACCCCTCATGATTGGTG<br>CACCAGACATGGCCTTCCCTCGAATAAATAACATGAGCTTTTGACTTCTCCCCCCCTCATTTCTT<br>CTTCTTCTCGCCTCATCTGGAGTCGAAGCAGGTGCCGGCACAGGATGGACTGTTTATCCCCCGC<br>TCGCAGGCAATCTTGCCCACGCTGGACCTTCTGTTGACTTAACCATCTTCTCCCTCCACTTGGC<br>CGGAGTGTCATCTATTTTAGGTGCAATTAATTTTATCACAACCATTATTAACATGAAACCCCCTGC<br>CATCTCCCAATATCAAACACCCCTATTTGTGTGATCCGTCCTAATTACCGCAGTACTACTCCTTCT<br>ATCCCTGCCCGTTCTTGCCGCCGGCATCACAATACTTCTAACAGACCGAAACCTAAACACAACC<br>TTCTTTGACCCTGCCGGAGGAGGAGACCCCATCCTATACCAACACTTA

KU565816	*O. niloticus*	TTGAGCCGGAATAGTAGGAACTGCACTAAGCCTCCTAATTCGGGCAGAACTAAGCCAGCCCGG<br>CTCTCTTCTCGGAGACGACCAAATCTATAATGTAATTGTTACAGCACATGCTTTCGTAATAATTTT<br>CTTTATAGTAATACCAATTATGATTGGAGGCTTTGGAAACTGACTAGTACCCCTCATGATTGGTG<br>CACCAGACATGGCCTTCCCTCGAATAAATAACATGAGCTTTTGACTTCTCCCCCCCTCATTTCTT<br>CTTCTTCTCGCCTCATCTGGAGTCGAAGCAGGTGCCGGCACAGGATGGACTGTTTATCCCCCGC<br>TCGCAGGCAATCTTGCCCACGCTGGACCTTCTGTTGACTTAACCATCTTCTCCCTCCACTTGGC<br>CGGAGTGTCATCTATTTTAGGTGCAATTAATTTTATCACAACCATTATTAACATGAAACCCCCTGC<br>CATCTCCCAATATCAAACACCCCTATTTGTGTGATCCGTCCTAATTACCGCAGTACTACTCCTTCT<br>ATCCCTGCCCGTTCTTGCCGCCGGCATCACAATACTTCTAACAGACCGAAACCTAAACACAACC<br>TTCTTTGACCCTGCCGGAGGAGGAGACCCCATCCTATACCAACACTTA
KU565819	*O. niloticus*	TTGAGCCGGAATAGTAGGAACTGCACTAAGCCTCCTAATTCGGGCAGAACTAAGCCAGCCCGG<br>CTCTCTTCTCGGAGACGACCAAATCTATAATGTAATTGTTACAGCACATGCTTTCGTAATAATTTT<br>CTTTATAGTAATACCAATTATGATTGGAGGCTTTGGAAACTGACTAGTACCCCTCATGATTGGTG<br>CACCAGACATGGCCTTCCCTCGAATAAATAACATGAGCTTTTGACTTCTCCCCCCCTCATTTCTT<br>CTTCTTCTCGCCTCATCTGGAGTCGAAGCAGGTGCCGGCACAGGATGGACTGTTTATCCCCCGC<br>TCGCAGGCAATCTTGCCCACGCTGGACCTTCTGTTGACTTAACCATCTTCTCCCTCCACTTGGC<br>CGGAGTGTCATCTATTTTAGGTGCAATTAATTTTATCACAACCATTATTAACATGAAACCCCCTGC<br>CATCTCCCAATATCAAACACCCCTATTTGTGTGATCCGTCCTAATTACCGCAGTACTACTCCTTCT<br>ATCCCTGCCCGTTCTTGCCGCCGGCATCACAATACTTCTAACAGACCGAAACCTAAACACAACC<br>TTCTTTGACCCTGCCGGAGGAGGAGACCCCATCCTATACCAACACTTA
KU565821	*O. niloticus*	TTGAGCTGGAATAGTAGGAACTGCACTAAGCCTCCTAATTCGGGCAGAAATAAGCCAGCCCGG<br>CTCTCTTCTCGGAGACGACCAAATCTATAATGTAATTGTTACAGCACATGCTTTCGTAATAATTTT<br>CTTTATAGTAATACCAATTATGATTGGAGGCTTTGGAAACTGACTAGTACCCCTCATGATTGGTG<br>CACCAGACATGGCCTTCCCTCGAATAAATAACATGAGCTTTTGACTTCTCCCCCCCTCATTTCTT<br>CTTCTTCTCGCCTCATCTGGAGTCGAAGCAGGTGCCGGCACAGGATGGACTGTTTATCCCCCGC<br>TCGCAGGCAATCTTGCCCACGCTGGACCTTCTGTTGACTTAACCATCTTCTCCCTCCACTTGGC<br>CGGAGTGTCATCTATTTTAGGTGCAATTAATTTTATCACAACCATTATTAACATGAAACCCCCTGC<br>CATCTCCCAATATCAAACACCCCTATTTGTGTGATCCGTCCTAATTACCGCAGTACTACTCCTTCT<br>ATCCCTGCCCGTTCTTGCCGCCGGCATCACAATACTTCTAACAGACCGAAACCTAAACACAACC<br>TTCTTTGACCCTGCCGGAGGAGGAGACCCCATCCTATACCAACACTTA
KU565822	*O. niloticus*	TTGAGCCGGAATAGTAGGAACTGCACTAAGCCTCCTAATTCGGGCAGAACTAAGCCAGCCCGG<br>CTCTCTTCTCGGAGACGACCAAATCTATAATGTAATTGTTACAGCACATGCTTTCGTAATAATTTT<br>CTTTATAGTAATACCAATTATGATTGGAGGCTTTGGAAACTGACTAGTACCCCTCATGATTGGTG<br>CACCAGACATGGCCTTCCCTCGAATAAATAACATGAGCTTTTGACTTCTCCCCCCCTCATTTCTT<br>CTTCTTCTCGCCTCATCTGGAGTCGAAGCAGGTGCCGGCACAGGATGGACTGTTTATCCCCCGC<br>TCGCAGGCAATCTTGCCCACGCTGGACCTTCTGTTGACTTAACCATCTTCTCCCTCCACTTGGC<br>CGGAGTGTCATCTATTTTAGGTGCAATTAATTTTATCACAACCATTATTAACATGAAACCCCCTGC<br>CATCTCCCAATATCAAACACCCCTATTTGTGTGATCCGTCCTAATTACCGCAGTACTACTCCTTCT<br>ATCCCTGCCCGTTCTTGCCGCCGGCATCACAATACTTCTAACAGACCGAAACCTAAACACAACC<br>TTCTTTGACCCTGCCGGAGGAGGAGACCCCATCCTATACCAACACTTA
KU565824	*O. niloticus*	TCGAGCCGGAATAGTAGGAACTGCAGTAAGCCTCCTAATTCGGGCAGAACTAAGCCAGCCCGG<br>CTCTCTTCTCGGAGACGACCAAATCTATAATGTAATTGTTACAGCACATGCTTTCGTAATAATTTT<br>CTTTATAGTAATACCAATTATGATTGGAGGCTTTGGAAACTGACTAGTACCCCTCATGATTGGTG<br>CACCAGACATGGCCTTCCCTCGAATAAATAACATGAGCTTTTGACTTCTCCCCCCCTCATTTCTT<br>CTTCTTCTCGCCTCATCTGGAGTCGAAGCAGGTGCCGGCACAGGATGGACTGTTTATCCCCCGC<br>TCGCAGGCAATCTTGCCCACGCTGGACCTTCTGTTGACTTAACCATCTTCTCCCTCCACTTGGC<br>CGGAGTGTCATCTATTTTAGGTGCAATTAATTTTATCACAACCATTATTAACATGAAACCCCCTGC<br>CATCTCCCAATATCAAACACCCCTATTTGTGTGATCCGTCCTAATTACCGCAGTACTACTCCTTCT<br>ATCCCTGCCCGTTCTTGCCGCCGGCATCACAATACTTCTAACAGACCGAAACCTAAACACAACC<br>TTCTTTGACCCTGCCGGAGGAGGAGACCCCATCCTATACCAACACTTA
KU565825	*O. niloticus*	TTGAGCTGGAATAGTAGGAACTGCACTAAGCCTCCTAATTCGGGCAGAACTAAGCCAGCCCGG<br>CTCTCTTCTCGGAGACGACCAAATCTATAATGTAATTGTTACAGCACATGCTTTCGTAATAATTTT<br>CTTTATAGTAATACCAATTATGATTGGAGGCTTTGGAAACTGACTAGTACCCCTCATGATTGGTG<br>CACCAGACATGGCCTTCCCTCGAATAAATAACATGAGCTTTTGACTTCTCCCCCCCTCATTTCTT<br>CTTCTTCTCGCCTCATCTGGAGTCGAAGCAGGTGCCGGCACAGGATGGACTGTTTATCCCCCGC<br>TCGCAGGCAATCTTGCCCACGCTGGACCTTCTGTTGACTTAACCATCTTCTCCCTCCACTTGGC<br>CGGAGTGTCATCTATTTTAGGTGCAATTAATTTTATCACAACCATTATTAACATGAAACCCCCTGC<br>CATCTCCCAATATCAAACACCCCTATTTGTGTGATCCGTCCTAATTACCGCAGTACTACTCCTTCT<br>ATCCCTGCCCGTTCTTGCCGCCGGCATCACAATACTTCTAACAGACCGAAACCTAAACACAACC<br>TTCTTTGACCCTGCCGGAGGAGGAGACCCCATCCTATACCAACACTTA
KU565827	*O. niloticus*	TTGAGCCGGAATAGTAGGAACTGCACTAAGCCTCCTAATTCGGGCAAAACTAAGCCAGCCCGG<br>CTCTCTTCTCGGAGACGACCAAATCTATAATGTAATTGTTACAGCACATGCTTTCGTAATAATTTT<br>CTTTATAGTAATACCAATTATGATTGGAGGCTTTGGAAACTGACTAGTACCCCTCATGATTGGTG<br>CACCAGACATGGCCTTCCCTCGAATAAATAACATGAGCTTTTGACTTCTCCCCCCCTCATTTCTT<br>CTTCTTCTCGCCTCATCTGGAGTCGAAGCAGGTGCCGGCACAGGATGGACTGTTTATCCCCCGC<br>TCGCAGGCAATCTTGCCCACGCTGGACCTTCTGTTGACTTAACCATCTTCTCCCTCCACTTGGC<br>CGGAGTGTCATCTATTTTAGGTGCAATTAATTTTATCACAACCATTATTAACATGAAACCCCCTGC<br>CATCTCCCAATATCAAACACCCCTATTTGTGTGATCCGTCCTAATTACCGCAGTACTACTCCTTCT<br>ATCCCTGCCCGTTCTTGCCGCCGGCATCACAATACTTCTAACAGACCGAAACCTAAACACAACC<br>TTCTTTGACCCTGCCGGAGGAGGAGACCCCATCCTATACCAACACTTA

KU565827.1	*O. niloticus*	TTGAGCCGGAATAGTAGGAACTGCACTAAGCCTCCTAATTCGGGCAAAACTAAGCCAGCCCGG<br>CTCTCTTCTCGGAGACGACCAAATCTATAATGTAATTGTTACAGCACATGCTTTCGTAATAATTTT<br>CTTTATAGTAATACCAATTATGATTGGAGGCTTTGGAAACTGACTAGTACCCCTCATGATTGGTG<br>CACCAGACATGGCCTTCCCTCGAATAAATAACATGAGCTTTTGACTTCTCCCCCCCTCATTTCTT<br>CTTCTTCTCGCCTCATCTGGAGTCGAAGCAGGTGCCGGCACAGGATGGACTGTTTATCCCCCGC<br>TCGCAGGCAATCTTGCCCACGCTGGACCTTCTGTTGACTTAACCATCTTCTCCCTCCACTTGGC<br>CGGAGTGTCATCTATTTTAGGTGCAATTAATTTTATCACAACCATTATTAACATGAAACCCCCTGC<br>CATCTCCCAATATCAAACACCCCTATTTGTGTGATCCGTCCTAATTACCGCAGTACTACTCCTTCT<br>ATCCCTGCCCGTTCTTGCCGCCGGCATCACAATACTTCTAACAGACCGAAACCTAAACACAACC<br>TTCTTTGACCCTGCCGGAGGAGGAGACCCCATCCTATACCAACACTTA
KU565828	*O. niloticus*	TTGAGCCGGAATAGTAGGAACTGCACTAAGCCTCCTAATTCGGGCAGAACTAAGCCAGCCCGG<br>CTCTCTTCTCGGAGACGACCAAATCTATAATGTAATTGTTACAGCACATGCTTTCGTAATAATTTT<br>CTTTATAGTAATACCAATTATGATTGGAGGCTTTGGAAACTGACTAGTACCCCTCATGATTGGTG<br>CACCAGACATGGCCTTCCCTCGAATAAATAACATGAGCTTTTGACTTCTCCCCCCCTCATTTCTT<br>CTTCTTCTCGCCTCATCTGGAGTCGAAGCAGGTGCCGGCACAGGATGGACTGTTTATCCCCCGC<br>TCGCAGGCAATCTTGCCCACGCTGGACCTTCTGTTGACTTAACCATCTTCTCCCTCCACTTGGC<br>CGGAGTGTCATCTATTTTAGGTGCAATTAATTTTATCACAACCATTATTAACATGAAACCCCCTGC<br>CATCTCCCAATATCAAACACCCCTATTTGTGTGATCCGTCCTAATTACCGCAGTACTACTCCTTCT<br>ATCCCTGCCCGTTCTTGCCGCCGGCATCACAATACTTCTAACAGACCGAAACCTAAACACAACC<br>TTCTTTGACCCTGCCGGAGGAGGAGACCCCATCCTATACCAACACTTA
KU565830	*O. niloticus*	TTGAGCCGGAATAGTAGGAACCGCGCTAAGCCTCCTAATTCGGGCAGAACTAAGCCAGCCCGG<br>CTCTCTCCTCGGAGACGACCAGATTTATAATGTAATTGTTACAGCACATGCTTTTGTAATAATTTT<br>CTTTATAGTAATGCCAATTATGATTGGAGGCTTTGGAAACTGACTAGTACCACTCATGATTGGTG<br>CCCCAGATATGGCCTTCCCTCGAATGAACAACATGAGTTTCTGACTCCTCCCTCCCTCATTCCTC<br>CTCCTCCTCGCCTCATCTGGAGTCGAAGCAGGTGCCGGCACAGGGTGAACTGTTTACCCCCCG<br>CTCGCAGGCAATCTTGCCCATGCTGGGCCTTCTGTCGACTTAACCATCTTCTCCCTCCACTTGGC<br>CGGGGTGTCATCTATTCTAGGCGCAATTAATTTCATTACAACAATCATTAACATGAAACCCCCCG<br>CCATCTCTCAATATCAAACACCCCTATTTGTATGGTCCGTTCTAATTACCGCAGTATTACTTCTTC<br>TATCCCTACCCGTTCTTGCCGCCGGCATCACAATACTTCTCACAGACCGAAACCTAAACACAAC<br>CTTCTTTGATCCTGCCGGAGGAGGAGACCCCATCCTTTACCAACACTTA
KU565831	*O. aureus*	TTGAGCCGGAATAGTAGGAACCGCGCTAAGCCTCCTAATTCGGGCAGAACTAAGCCAGCCCGG<br>CTCTCTCCTCGGAGACGACCAGATTTATAATGTAATTGTTACAGCACATGCTTTTGTAATAATTTT<br>CTTTATAGTAATGCCAATTATGATTGGAGGCTTTGGAAACTGACTAGTACCACTCATGATTGGTG<br>CTCCAGATATGGCCTTCCCTCGAATGAACAACATGAGTTTCTGACTCCTCCCTCCCTCATTCCTC<br>CTCCTCCTCGCCTCATCTGGAGTCGAAGCAGGTGCCGGCACAGGGTGAACTGTTTACCCCCCG<br>CTCGCAGGCAATCTTGCCCATGCTGGGCCTTCTGTCGACTTAACCATCTTCTCCCTCCACTTGGC<br>CGGGGTGTCATCTATTCTAGGCGCAATTAATTTCATTACAACAATCATTAACATGAAACCCCCCG<br>CCATCTCTCAATATCAAACACCTCTATTTGTATGGTCCGTTCTAATTACCGCAGTATTACTTCTTC<br>TATCCCTACCCGTTCTTGCCGCCGGCATCACAATACTTCTCACAGACCGAAACCTAAACACAAC<br>CTTCTTTGATCCTGCCGGAGGAGGAGACCCCATCCTTTACCAACACTTA
KU565832	*O. niloticus*	TTGAGCCGGAATAGTAGGAACTGCACTAAGCCTCCTAATTCGGGCAGAACTAAGCCAGCCCGG<br>CTCTCTTCTCGGAGACGACCAAATCTATAATGTAATTGTTACAGCACATGCTTTCGTAATAATTTT<br>CTTTATAGTAATACCAATTATGATTGGAGGCTTTGGAAACTGACTAGTACCCCTCATGATTGGTG<br>CACCAGACATGGCCTTCCCTCGAATAAATAACATGAGCTTTTGACTTCTCCCCCCCTCATTTCTT<br>CTTCTTCTCGCCTCATCTGGAGTCGAAGCAGGTGCCGGCACAGGATGGACTGTTTATCCCCCGC<br>TCGCAGGCAATCTTGCCCACGCTGGACCTTCTGTTGACTTAACCATCTTCTCCCTCCACTTGGC<br>CGGAGTGTCATCTATTTTAGGTGCAATTAATTTTATCACAACCATTATTAACATGAAACCCCCTGC<br>CATCTCCCAATATCAAACACCCCTATTTGTGTGATCCGTCCTAATTACCGCAGTACTACTCCTTCT<br>ATCCCTGCCCGTTCTTGCCGCCGGCATCACAATACTTCTAACAGACCGAAACCTAAACACAACC<br>TTCTTTGACCCTGCCGGAGGAGGAGACCCCATCCTATACCAACACTTA
KU565833	*O. niloticus*	TTGAGCCGGAATAGTAGGAACTGCACTAAGCCTCCTAATTCGGGCAGAACTAAGCCAGCCCGG<br>CTCTCTTCTCGGAGACGACCAAATCTATAATGTAATTGTTACAGCACATGCTTTCGTAATAATTTT<br>CTTTATAGTAATACCAATTATGATTGGAGGCTTTGGAAACTGACTAGTACCCCTCATGATTGGTG<br>CACCAGACATGGCCTTCCCTCGAATAAATAACATGAGCTTTTGACTTCTCCCCCCCTCATTTCTT<br>CTTCTTCTCGCCTCATCTGGAGTCGAAGCAGGTGCCGGCACAGGATGGACTGTTTATCCCCCGC<br>TCGCAGGCAATCTTGCCCACGCTGGACCTTCTGTTGACTTAACCATCTTCTCCCTCCACTTGGC<br>CGGAGTGTCATCTATTTTAGGTGCAATTAATTTTATCACAACCATTATTAACATGAAACCCCCTGC<br>CATCTCCCAATATCAAACACCCCTATTTGTGTGATCCGTCCTAATTACCGCAGTACTACTCCTTCT<br>ATCCCTGCCCGTTCTTGCCGCCGGCATCACAATACTTCTAACAGACCGAAACCTAAACACAACC<br>TTCTTTGACCCTGCCGGAGGAGGAGACCCCATCCTATACCAACACTTA
KU565834	*O. niloticus*	TTGAGCCGGAATAGTAGGAACTGCACTAAGCCTCCTAATTCGGGCAGAACTAAGCCAGCCCGG<br>CTCTCTTCTCGGAGACGACCAAATCTATAATGTAATTGTTACAGCACATGCTTTCGTAATAATTTT<br>CTTTATAGTAATACCAATTATGATTGGAGGCTTTGGAAACTGACTAGTACCCCTCATGATTGGTG<br>CACCAGACATGGCCTTCCCTCGAATAAATAACATGAGCTTTTGACTTCTCCCCCCCTCATTTCTT<br>CTTCTTCTCGCCTCATCTGGAGTCGAAGCAGGTGCCGGCACAGGATGGACTGTTTATCCCCCGC<br>TCGCAGGCAATCTTGCCCACGCTGGACCTTCTGTTGACTTAACCATCTTCTCCCTCCACTTGGC<br>CGGAGTGTCATCTATTTTAGGTGCAATTAATTTTATCACAACCATTATTAACATGAAACCCCCTGC<br>CATCTCCCAATATCAAACACCCCTATTTGTGTGATCCGTCCTAATTACCGCAGTACTACTCCTTCT<br>ATCCCTGCCCGTTCTTGCCGCCGGCATCACAATACTTCTAACAGACCGAAACCTAAACACAACC<br>TTCTTTGACCCTGCCGGAGGAGGAGACCCCATCCTATACCAACACTTA

KU565835	*O. niloticus*	TTGAGCCGGAATAGTAGGAACTGCACTAAGCCTCCTAATTCGGGCAGAACTAAGCCAGCCCGG<br>CTCTCTTCTCGGAGACGACCAAATCTATAATGTAATTGTTACAGCACATGCTTTCGTAATAATTTT<br>CTTTATAGTAATACCAATTATGATTGGAGGCTTTGGAAACTGACTAGTACCCCTCATGATTGGTG<br>CACCAGACATGGCCTTCCCTCGAATAAATAACATGAGCTTTTGACTTCTCCCCCCCTCATTTCTT<br>CTTCTTCTCGCCTCATCTGGAGTCGAAGCAGGTGCCGGCACAGGATGGACTGTTTATCCCCCGC<br>TCGCAGGCAATCTTGCCCACGCTGGACCTTCTGTTGACTTAACCATCTTCTCCCTCCACTTGGC<br>CGGAGTGTCATCTATTTTAGGTGCAATTAATTTTATCACAACCATTATTAACATGAAACCCCCTGC<br>CATCTCCCAATATCAAACACCCCTATTTGTGTGATCCGTCCTAATTACCGCAGTACTACTCCTTCT<br>ATCCCTGCCCGTTCTTGCCGCCGGCATCACAATACTTCTAACAGACCGAAACCTAAACACAACC<br>TTCTTTGACCCTGCCGGAGGAGGAGACCCCATCCTATACCAACACTTA
KU565836	*O. niloticus*	TTGAGCCGGAATAGTAGGAACTGCACTAAGCCTCCTAATTCGGGCAGAACTAAGCCAGCCCGG<br>CTCTCTTCTCGGAGACGACCAAATCTATAATGTAATTGTTACAGCACATGCTTTCGTAATAATTTT<br>CTTTATAGTAATACCAATTATGATTGGAGGCTTTGGAAACTGACTAGTACCCCTCATGATTGGTG<br>CACCAGACATGGCCTTCCCTCGAATAAATAACATGAGCTTTTGACTTCTCCCCCCCTCATTTCTT<br>CTTCTTCTCGCCTCATCTGGAGTCGAAGCAGGTGCCGGCACAGGATGGACTGTTTATCCCCCGC<br>TCGCAGGCAATCTTGCCCACGCTGGACCTTCTGTTGACTTAACCATCTTCTCCCTCCACTTGGC<br>CGGAGTGTCATCTATTTTAGGTGCAATTAATTTTATCACAACCATTATTAACATGAAACCCCCTGC<br>CATCTCCCAATATCAAACACCCCTATTTGTGTGATCCGTCCTAATTACCGCAGTACTACTCCTTCT<br>ATCCCTGCCCGTTCTTGCCGCCGGCATCACAATACTTCTAACAGACCGAAACCTAAACACAACC<br>TTCTTTGACCCTGCCGGAGGAGGAGACCCCATCCTATACCAACACTTA
KU565837	*O. niloticus*	TTGAGCCGGAATAGTAGGAACTGCACTAAGCCTCCTAATTCGGGCAGAACTAAGCCAGCCCGG<br>CTCTCTTCTCGGAGACGACCAAATCTATAATGTAATTGTTACAGCACATGCTTTCGTAATAATTTT<br>CTTTATAGTAATACCAATTATGATTGGAGGCTTTGGAAACTGACTAGTACCCCTCATGATTGGTG<br>CACCAGACATGGCCTTCCCTCGAATAAATAACATGAGCTTTTGACTTCTCCCCCCCTCATTTCTT<br>CTTCTTCTCGCCTCATCTGGAGTCGAAGCAGGTGCCGGCACAGGATGGACTGTTTATCCCCCGC<br>TCGCAGGCAATCTTGCCCACGCTGGACCTTCTGTTGACTTAACCATCTTCTCCCTCCACTTGGC<br>CGGAGTGTCATCTATTTTAGGTGCAATTAATTTTATCACAACCATTATTAACATGAAACCCCCTGC<br>CATCTCCCAATATCAAACACCCCTATTTGTGTGATCCGTCCTAATTACCGCAGTACTACTCCTTCT<br>ATCCCTGCCCGTTCTTGCCGCCGGCATCACAATACTTCTAACAGACCGAAACCTAAACACAACC<br>TTCTTTGACCCTGCCGGAGGAGGAGACCCCATCCTATACCAACACTTA
KU565838	*O. niloticus*	TCGAGCCCGAATAGTAGGAACTGCAGTAAGCCTCCTAATTCGGGCAGAACTAAGCCAGCCCGG<br>CTCTCTTCTCGGAGACGACCAAATCTATAATGTAATTGTTACAGCACATGCTTTCGTAATAATTTT<br>CTTTATAGTAATACCAATTATGATTGGAGGCTTTGGAAACTGACTAGTACCCCTCATGATTGGTG<br>CACCAGACATGGCCTTCCCTCGAATAAATAACATGAGCTTTTGACTTCTCCCCCCCTCATTTCTT<br>CTTCTTCTCGCCTCATCTGGAGTCGAAGCAGGTGCCGGCACAGGATGGACTGTTTATCCCCCGC<br>TCGCAGGCAATCTTGCCCACGCTGGACCTTCTGTTGACTTAACCATCTTCTCCCTCCACTTGGC<br>CGGAGTGTCATCTATTTTAGGTGCAATTAATTTTATCACAACCATTATTAACATGAAACCCCCTGC<br>CATCTCCCAATATCAAACACCCCTATTTGTGTGATCCGTCCTAATTACCGCAGTACTACTCCTTCT<br>ATCCCTGCCCGTTCTTGCCGCCGGCATCACAATACTTCTAACAGACCGAAACCTAAACACAACC<br>TTCTTTGACCCTGCCGGAGGAGGAGACCCCATCCTATACCAACACTTA
KU565840	*O. niloticus*	TTGAGCCGGAATAGTAGGAACTGCACTAAGCCTCCTAATTCGGGCAGAACTAAGCCAGCCCGG<br>CTCTCTTCTCGGAGACGACCAAATCTATAATGTAATTGTTACAGCACATGCTTTCGTAATAATTTT<br>CTTTATAGTAATACCAATTATGATTGGAGGCTTTGGAAACTGACTAGTACCCCTCATGATTGGTG<br>CACCAGACATGGCCTTCCCTCGAATAAATAACATGAGCTTTTGACTTCTCCCCCCCTCATTTCTT<br>CTTCTTCTCGCCTCATCTGGAGTCGAAGCAGGTGCCGGCACAGGATGGACTGTTTATCCCCCGC<br>TCGCAGGCAATCTTGCCCACGCTGGACCTTCTGTTGACTTAACCATCTTCTCCCTCCACTTGGC<br>CGGAGTGTCATCTATTTTAGGTGCAATTAATTTTATCACAACCATTATTAACATGAAACCCCCTGC<br>CATCTCCCAATATCAAACACCCCTATTTGTGTGATCCGTCCTAATTACCGCAGTACTACTCCTTCT<br>ATCCCTGCCCGTTCTTGCCGCCGGCATCACAATACTTCTAACAGACCGAAACCTAAACACAACC<br>TTCTTTGACCCTGCCGGAGGAGGAGACCCCATCCTATACCAACACTTA
KU565841	*O. niloticus*	TTGAGCTGGAATAGTAGGAACTGCACTAAGCCTCCTAATTCGGGCAGAACTAAGCCAGCCCGG<br>CTCTCTTCTCGGAGACGACCAAATCTATAATGTAATTGTTACAGCACATGCTTTCGTAATAATTTT<br>CTTTATAGTAATACCAATTATGATTGGAGGCTTTGGAAACTGACTAGTACCCCTCATGATTGGTG<br>CACCAGACATGGCCTTCCCTCGAATAAATAACATGAGCTTTTGACTTCTCCCCCCCTCATTTCTT<br>CTTCTTCTCGCCTCATCTGGAGTCGAAGCAGGTGCCGGCACAGGATGGACTGTTTATCCCCCGC<br>TCGCAGGCAATCTTGCCCACGCTGGACCTTCTGTTGACTTAACCATCTTCTCCCTCCACTTGGC<br>CGGAGTGTCATCTATTTTAGGTGCAATTAATTTTATCACAACCATTATTAACATGAAACCCCCTGC<br>CATCTCCCAATATCAAACACCCCTATTTGTGTGATCCGTCCTAATTACCGCAGTACTACTCCTTCT<br>ATCCCTGCCCGTTCTTGCCGCCGGCATCACAATACTTCTAACAGACCGAAACCTAAACACAACC<br>TTCTTTGACCCTGCCGGAGGAGGAGACCCCATCCTATACCAACACTTA
KU565842	*O. niloticus*	TTGAGCCGGAATAGTAGGAACTGCACTAAGCCTCCTAATTCGGGCAGAACTAAGCCAGCCCGG<br>CTCTCTTCTCGGAGACGACCAAATCTATAATGTAATTGTTACAGCACATGCTTTCGTAATAATTTT<br>CTTTATAGTAATACCAATTATGATTGGAGGCTTTGGAAACTGACTAGTACCCCTCATGATTGGTG<br>CACCAGACATGGCCTTCCCTCGAATAAATAACATGAGCTTTTGACTTCTCCCCCCCTCATTTCTT<br>CTTCTTCTCGCCTCATCTGGAGTCGAAGCAGGTGCCGGCACAGGATGGACTGTTTATCCCCCGC<br>TCGCAGGCAATCTTGCCCACGCTGGACCTTCTGTTGACTTAACCATCTTCTCCCTCCACTTGGC<br>CGGAGTGTCATCTATTTTAGGTGCAATTAATTTTATCACAACCATTATTAACATGAAACCCCCTGC<br>CATCTCCCAATATCAAACACCCCTATTTGTGTGATCCGTCCTAATTACCGCAGTACTACTCCTTCT<br>ATCCCTGCCCGTTCTTGCCGCCGGCATCACAATACTTCTAACAGACCGAAACCTAAACACAACC<br>TTCTTTGACCCTGCCGGAGGAGGAGACCCCATCCTATACCAACACTTA

KU565843	*O. niloticus*	TTGAGCCGGAATAGTAGGAACTGCACTAAGCCTCCTAATTCGGGCAGAACTAAGCCAGCCCGG<br>CTCTCTTCTCGGAGACGACCAAATCTATAATGTAATTGTTACAGCACATGCTTTCGTAATAATTTT<br>CTTTATAGTAATACCAATTATGATTGGAGGCTTTGGAAACTGACTAGTACCCCTCATGATTGGTG<br>CACCAGACATGGCCTTCCCTCGAATAAATAACATGAGCTTTTGACTTCTCCCCCCCTCATTTCTT<br>CTTCTTCTCGCCTCATCTGGAGTCGAAGCAGGTGCCGGCACAGGATGGACTGTTTATCCCCCGC<br>TCGCAGGCAATCTTGCCCACGCTGGACCTTCTGTTGACTTAACCATCTTCTCCCTCCACTTGGC<br>CGGAGTGTCATCTATTTTAGGTGCAATTAATTTTATCACAACCATTATTAACATGAAACCCCCTGC<br>CATCTCCCAATATCAAACACCCCTATTTGTGTGATCCGTCCTAATTACCGCAGTACTACTCCTTCT<br>ATCCCTGCCCGTTCTTGCCGCCGGCATCACAATACTTCTAACAGACCGAAACCTAAACACAACC<br>TTCTTTGACCCTGCCGGAGGAGGAGACCCCATCCTATACCAACACTTA
KU565843.1	*O. niloticus*	TTGAGCCGGAATAGTAGGAACTGCACTAAGCCTCCTAATTCGGGCAGAACTAAGCCAGCCCGG<br>CTCTCTTCTCGGAGACGACCAAATCTATAATGTAATTGTTACAGCACATGCTTTCGTAATAATTTT<br>CTTTATAGTAATACCAATTATGATTGGAGGCTTTGGAAACTGACTAGTACCCCTCATGATTGGTG<br>CACCAGACATGGCCTTCCCTCGAATAAATAACATGAGCTTTTGACTTCTCCCCCCCTCATTTCTT<br>CTTCTTCTCGCCTCATCTGGAGTCGAAGCAGGTGCCGGCACAGGATGGACTGTTTATCCCCCGC<br>TCGCAGGCAATCTTGCCCACGCTGGACCTTCTGTTGACTTAACCATCTTCTCCCTCCACTTGGC<br>CGGAGTGTCATCTATTTTAGGTGCAATTAATTTTATCACAACCATTATTAACATGAAACCCCCTGC<br>CATCTCCCAATATCAAACACCCCTATTTGTGTGATCCGTCCTAATTACCGCAGTACTACTCCTTCT<br>ATCCCTGCCCGTTCTTGCCGCCGGCATCACAATACTTCTAACAGACCGAAACCTAAACACAACC<br>TTCTTTGACCCTGCCGGAGGAGGAGACCCCATCCTATACCAACACTTA
KU565844	*O. aureus*	CTGAGCCGGAATAGTAGGAACCGCGGTAAGCTTCCTAATTCGGGTAGAACTAAGCCAGCCCGG<br>CTCTCTCCTCGGAGACGACCAGATTTATAATGTAATTGTTACAGCACATGCTTTTGTAATAATTTT<br>CTTTATAGTAATGCCAATTATGATTGGAGGCTTTGGAAACTGACTAGTACCACTCATGATTGGTG<br>CCCCAGATATGGCCTTCCCTCGAATGAACAACATGAGTTTCTGACTCCTCCCTCCCTCATTCCTC<br>CTCCTCCTCGCCTCATCTGGAGTCGAAGCAGGTGCCGGCACAGGGTGAACTGTTTACCCCCCG<br>CTCGCAGGCAATCTTGCCCATGCTGGGCCTTCTGTCGACTTAACCATCTTCTCCCTCCACTTGGC<br>CGGGGTGTCATCTATTCTAGGCGCAATTAATTTCATTACAACAATCATTAACATGAAACCCCCCG<br>CCATCTCTCAATATCAAACACCCCTATTTGTATGGTCCGTTCTAATTACCGCAGTATTACTTCTTC<br>TATCCCTACCCGTTCTTGCCGCCGGCATCACAATACTTCTCACAGACCGAAACCTAAACACAAC<br>CTTCTTTGATCCTGCCGGAGGAGGAGACCCCATCCTTTACCAACACTTA
KU565845	*O. niloticus*	TTGAGCCGGAATAGTAGGAACTGCACTAAGCCTCCTAATTCGGGCAGAACTAAGCCAGCCCGG<br>CTCTCTTCTCGGAGACGACCAAATCTATAATGTAATTGTTACAGCACATGCTTTCGTAATAATTTT<br>CTTTATAGTAATACCAATTATGATTGGAGGCTTTGGAAACTGACTAGTACCCCTCATGATTGGTG<br>CACCAGACATGGCCTTCCCTCGAATAAATAACATGAGCTTTTGACTTCTCCCCCCCTCATTTCTT<br>CTTCTTCTCGCCTCATCTGGAGTCGAAGCAGGTGCCGGCACAGGATGGACTGTTTATCCCCCGC<br>TCGCAGGCAATCTTGCCCACGCTGGACCTTCTGTTGACTTAACCATCTTCTCCCTCCACTTGGC<br>CGGAGTGTCATCTATTTTAGGTGCAATTAATTTTATCACAACCATTATTAACATGAAACCCCCTGC<br>CATCTCCCAATATCAAACACCCCTATTTGTGTGATCCGTCCTAATTACCGCAGTACTACTCCTTCT<br>ATCCCTGCCCGTTCTTGCCGCCGGCATCACAATACTTCTAACAGACCGAAACCTAAACACAACC<br>TTCTTTGACCCTGCCGGAGGAGGAGACCCCATCCTATACCAACACTTA
KU565846	*O. niloticus*	TTGAGCCGGAATAGTAGGAACTGCACTAAGCCTCCTAATTCGGGCAGAACTAAGCCAGCCCGG<br>CTCTCTTCTCGGAGACGACCAAATCTATAATGTAATTGTTACAGCACATGCTTTCGTAATAATTTT<br>CTTTATAGTAATACCAATTATGATTGGAGGCTTTGGAAACTGACTAGTACCCCTCATGATTGGTG<br>CACCAGACATGGCCTTCCCTCGAATAAATAACATGAGCTTTTGACTTCTCCCCCCCTCATTTCTT<br>CTTCTTCTCGCCTCATCTGGAGTCGAAGCAGGTGCCGGCACAGGATGGACTGTTTATCCCCCGC<br>TCGCAGGCAATCTTGCCCACGCTGGACCTTCTGTTGACTTAACCATCTTCTCCCTCCACTTGGC<br>CGGAGTGTCATCTATTTTAGGTGCAATTAATTTTATCACAACCATTATTAACATGAAACCCCCTGC<br>CATCTCCCAATATCAAACACCCCTATTTGTGTGATCCGTCCTAATTACCGCAGTACTACTCCTTCT<br>ATCCCTGCCCGTTCTTGCCGCCGGCATCACAATACTTCTAACAGACCGAAACCTAAACACAACC<br>TTCTTTGACCCTGCCGGAGGAGGAGACCCCATCCTATACCAACACTTA
KU565848	*O. niloticus*	TTGAGCCGGAATAGTAGGAACTGCACTAAGCCTCCTAATTCGGGCAGAACTAAGCCAGCCCGG<br>CTCTCTTCTCGGAGACGACCAAATCTATAATGTAATTGTTACAGCACATGCTTTCGTAATAATTTT<br>CTTTATAGTAATACCAATTATGATTGGAGGCTTTGGAAACTGACTAGTACCCCTCATGATTGGTG<br>CACCAGACATGGCCTTCCCTCGAATAAATAACATGAGCTTTTGACTTCTCCCCCCCTCATTTCTT<br>CTTCTTCTCGCCTCATCTGGAGTCGAAGCAGGTGCCGGCACAGGATGGACTGTTTATCCCCCGC<br>TCGCAGGCAATCTTGCCCACGCTGGACCTTCTGTTGACTTAACCATCTTCTCCCTCCACTTGGC<br>CGGAGTGTCATCTATTTTAGGTGCAATTAATTTTATCACAACCATTATTAACATGAAACCCCCTGC<br>CATCTCCCAATATCAAACACCCCTATTTGTGTGATCCGTCCTAATTACCGCAGTACTACTCCTTCT<br>ATCCCTGCCCGTTCTTGCCGCCGGCATCACAATACTTCTAACAGACCGAAACCTAAACACAACC<br>TTCTTTGACCCTGCCGGAGGAGGAGACCCCATCCTATACCAACACTTA
KU565849	*O. niloticus*	TTGAGCCGGAATAGTAGGAACTGCACTAAGCCTCCTAATTCGGGCAGAACTAAGCCAGCCCGG<br>CTCTCTTCTCGGAGACGACCAAATCTATAATGTAATTGTTACAGCACATGCTTTCGTAATAATTTT<br>CTTTATAGTAATACCAATTATGATTGGAGGCTTTGGAAACTGACTAGTACCCCTCATGATTGGTG<br>CACCAGACATGGCCTTCCCTCGAATAAATAACATGAGCTTTTGACTTCTCCCCCCCTCATTTCTT<br>CTTCTTCTCGCCTCATCTGGAGTCGAAGCAGGTGCCGGCACAGGATGGACTGTTTATCCCCCGC<br>TCGCAGGCAATCTTGCCCACGCTGGACCTTCTGTTGACTTAACCATCTTCTCCCTCCACTTGGC<br>CGGAGTGTCATCTATTTTAGGTGCAATTAATTTTATCACAACCATTATTAACATGAAACCCCCTGC<br>CATCTCCCAATATCAAACACCCCTATTTGTGTGATCCGTCCTAATTACCGCAGTACTACTCCTTCT<br>ATCCCTGCCCGTTCTTGCCGCCGGCATCACAATACTTCTAACAGACCGAAACCTAAACACAACC<br>TTCTTTGACCCTGCCGGAGGAGGAGACCCCATCCTATACCAACACTTA

KU565851	*O. aureus*	TTGAGCCGGAATAGTAGGAACCGCGCTAAGCCTCCTAATTCGGGCAGAACTAAGCCAGCCCGG<br>CTCTCTCCTCGGAGACGACCAGATTTATAATGTAATTGTTACAGCACATGCTTTTGTAATAATTTT<br>CTTTATAGTAATGCCAATTATGATTGGAGGCTTTGGAAACTGACTAGTACCACTCATGATTGGTG<br>CCCCAGATATGGCCTTCCCTCGAATGAACAACATGAGTTTCTGACTCCTCCCTCCCTCATTCCTC<br>CTCCTCCTCGCCTCATCTGGAGTCGAAGCAGGTGCCGGCACAGGGTGAACTGTTTACCCCCCG<br>CTCGCAGGCAATCTTGCCCATGCTGGGCCTTCTGTCGACTTAACCATCTTCTCCCTCCACTTGGC<br>CGGGGTGTCATCTATTCTAGGCGCAATTAATTTCATTACAACAATCATTAACATGAAACCCCCCG<br>CCATCTCTCAATATCAAACACCCCTATTTGTATGGTCCGTTCTAATTACCGCAGTATTACTTCTTC<br>TATCCCTACCCGTTCTTGCCGCCGGCATCACAATACTTCTCACAGACCGAAACCTAAACACAAC<br>CTTCTTTGATCCTGCCGGAGGAGGAGACCCCATCCTTTACCAACACTTA
KU565852	*O. aureus*	TTGAGCCGGAATCGTAGGAACCGCGCTAAGCCTCCTAATTCGGGCAGAACTAAGCCAGCCCGG<br>CTCTCTCCTCGGAGACGACCAGATTTATAATGTAATTGTTACAGCACATGCTTTTGTAATAATTTT<br>CTTTATAGTAATGCCAATTATGATTGGAGGCTTTGGAAACTGACTAGTACCACTCATGATTGGTG<br>CCCCAGATATGGCCTTCCCTCGAATGAACAACATGAGTTTCTGACTCCTCCCTCCCTCATTCCTC<br>CTCCTCCTCGCCTCATCTGGAGTCGAAGCAGGTGCCGGCACAGGGTGAACTGTTTACCCCCCG<br>CTCGCAGGCAATCTTGCCCATGCTGGGCCTTCTGTCGACTTAACCATCTTCTCCCTCCACTTGGC<br>CGGGGTGTCATCTATTCTAGGCGCAATTAATTTCATTACAACAATCATTAACATGAAACCCCCCG<br>CCATCTCTCAATATCAAACACCCCTATTTGTATGGTCCGTTCTAATTACCGCAGTATTACTTCTTC<br>TATCCCTACCCGTTCTTGCCGCCGGCATCACAATACTTCTCACAGACCGAAACCTAAACACAAC<br>CTTCTTTGATCCTGCCGGAGGAGGAGACCCCATCCTTTACCAACACTTA
KU565857	*O. niloticus*	TTGAGCCGGAATAGTAGGAACTGCACTAAGCCTCCTAATTCGGGCAGAACTAAGCCAGCCCGG<br>CTCTCTTCTCGGAGACGACCAAATCTATAATGTAATTGTTACAGCACATGCTTTCGTAATAATTTT<br>CTTTATAGTAATACCAATTATGATTGGAGGCTTTGGAAACTGACTAGTACCCCTCATGATTGGTG<br>CACCAGACATGGCCTTCCCTCGAATAAATAACATGAGCTTTTGACTTCTCCCCCCCTCATTTCTT<br>CTTCTTCTCGCCTCATCTGGAGTCGAAGCAGGTGCCGGCACAGGATGGACTGTTTATCCCCCGC<br>TCGCAGGCAATCTTGCCCACGCTGGACCTTCTGTTGACTTAACCATCTTCTCCCTCCACTTGGC<br>CGGAGTGTCATCTATTTTAGGTGCAATTAATTTTATCACAACCATTATTAACATGAAACCCCCTGC<br>CATCTCCCAATATCAAACACCCCTATTTGTGTGATCCGTCCTAATTACCGCAGTACTACTCCTTCT<br>ATCCCTGCCCGTTCTTGCCGCCGGCATCACAATACTTCTAACAGACCGAAACCTAAACACAACC<br>TTCTTTGACCCTGCCGGAGGAGGAGACCCCATCCTATACCAACACTTA
KU565858	*O. niloticus*	TTGAGCTGGAATAGTAGGAACTGCACTAAGCCTCCTAATTCGGGCAGAACTAAGCCAGCCCGG<br>CTCTCTTCTCGGAGACGACCAAATCTATAATGTAATTGTTACAGCACATGCTTTCGTAATAATTTT<br>CTTTATAGTAATACCAATTATGATTGGAGGCTTTGGAAACTGACTAGTACCCCTCATGATTGGTG<br>CACCAGACATGGCCTTCCCTCGAATAAATAACATGAGCTTTTGACTTCTCCCCCCCTCATTTCTT<br>CTTCTTCTCGCCTCATCTGGAGTCGAAGCAGGTGCCGGCACAGGATGGACTGTTTATCCCCCGC<br>TCGCAGGCAATCTTGCCCACGCTGGACCTTCTGTTGACTTAACCATCTTCTCCCTCCACTTGGC<br>CGGAGTGTCATCTATTTTAGGTGCAATTAATTTTATCACAACCATTATTAACATGAAACCCCCTGC<br>CATCTCCCAATATCAAACACCCCTATTTGTGTGATCCGTCCTAATTACCGCAGTACTACTCCTTCT<br>ATCCCTGCCCGTTCTTGCCGCCGGCATCACAATACTTCTAACAGACCGAAACCTAAACACAACC<br>TTCTTTGACCCTGCCGGAGGAGGAGACCCCATCCTATACCAACACTTA
KU565859	*O. niloticus*	TTGAGCCGGAATAGTAGGAACTGCACTAAGCCTCCTAATTCGGGCAGAACTAAGCCAGCCCGG<br>CTCTCTTCTCGGAGACGACCAAATCTATAATGTAATTGTTACAGCACATGCTTTCGTAATAATTTT<br>CTTTATAGTAATACCAATTATGATTGGAGGCTTTGGAAACTGACTAGTACCCCTCATGATTGGTG<br>CACCAGACATGGCCTTCCCTCGAATAAATAACATGAGCTTTTGACTTCTCCCCCCCTCATTTCTT<br>CTTCTTCTCGCCTCATCTGGAGTCGAAGCAGGTGCCGGCACAGGATGGACTGTTTATCCCCCGC<br>TCGCAGGCAATCTTGCCCACGCTGGACCTTCTGTTGACTTAACCATCTTCTCCCTCCACTTGGC<br>CGGAGTGTCATCTATTTTAGGTGCAATTAATTTTATCACAACCATTATTAACATGAAACCCCCTGC<br>CATCTCCCAATATCAAACACCCCTATTTGTGTGATCCGTCCTAATTACCGCAGTACTACTCCTTCT<br>ATCCCTGCCCGTTCTTGCCGCCGGCATCACAATACTTCTAACAGACCGAAACCTAAACACAACC<br>TTCTTTGACCCTGCCGGAGGAGGAGACCCCATCCTATACCAACACTTA
KU565860	*O. niloticus*	TTGAGCCGGAATAGTAGGAACTGCACTAAGCCTCCTAATTCGGGCAGAACTAAGCCAGCCCGG<br>CTCTCTTCTCGGAGACGACCAAATCTATAATGTAATTGTTACAGCACATGCTTTCGTAATAATTTT<br>CTTTATAGTAATACCAATTATGATTGGAGGCTTTGGAAACTGACTAGTACCCCTCATGATTGGTG<br>CACCAGACATGGCCTTCCCTCGAATAAATAACATGAGCTTTTGACTTCTCCCCCCCTCATTTCTT<br>CTTCTTCTCGCCTCATCTGGAGTCGAAGCAGGTGCCGGCACAGGATGGACTGTTTATCCCCCGC<br>TCGCAGGCAATCTTGCCCACGCTGGACCTTCTGTTGACTTAACCATCTTCTCCCTCCACTTGGC<br>CGGAGTGTCATCTATTTTAGGTGCAATTAATTTTATCACAACCATTATTAACATGAAACCCCCTGC<br>CATCTCCCAATATCAAACACCCCTATTTGTGTGATCCGTCCTAATTACCGCAGTACTACTCCTTCT<br>ATCCCTGCCCGTTCTTGCCGCCGGCATCACAATACTTCTAACAGACCGAAACCTAAACACAACC<br>TTCTTTGACCCTGCCGGAGGAGGAGACCCCATCCTATACCAACACTTA
KU565863	*O. niloticus*	TTGAGCCGGAATAGTAGGAACTGCACTAAGCCTCCTAATTCGGGCAGAACTAAGCCAGCCCGG<br>CTCTCTTCTCGGAGACGACCAAATCTATAATGTAATTGTTACAGCACATGCTTTCGTAATAATTTT<br>CTTTATAGTAATACCAATTATGATTGGAGGCTTTGGAAACTGACTAGTACCCCTCATGATTGGTG<br>CACCAGACATGGCCTTCCCTCGAATAAATAACATGAGCTTTTGACTTCTCCCCCCCTCATTTCTT<br>CTTCTTCTCGCCTCATCTGGAGTCGAAGCAGGTGCCGGCACAGGATGGACTGTTTATCCCCCGC<br>TCGCAGGCAATCTTGCCCACGCTGGACCTTCTGTTGACTTAACCATCTTCTCCCTCCACTTGGC<br>CGGAGTGTCATCTATTTTAGGTGCAATTAATTTTATCACAACCATTATTAACATGAAACCCCCTGC<br>CATCTCCCAATATCAAACACCCCTATTTGTGTGATCCGTCCTAATTACCGCAGTACTACTCCTTCT<br>ATCCCTGCCCGTTCTTGCCGCCGGCATCACAATACTTCTAACAGACCGAAACCTAAACACAACC<br>TTCTTTGACCCTGCCGGAGGAGGAGACCCCATCCTATACCAACACTTA

KU565864	*O. niloticus*	TTGAGCCGGAATAGTAGGAACTGCACTAAGCCTCCTAATTCGGGCAGAACTAAGCCAGCCCGG CTCTCTTCTCGGAGACGACCAAATCTATAATGTAATTGTTACAGCACATGCTTTCGTAATAATTTT CTTTATAGTAATACCAATTATGATTGGAGGCTTTGGAAACTGACTAGTACCCCTCATGATTGGTG CACCAGACATGGCCTTCCCTCGAATAAATAACATGAGCTTTTGACTTCTCCCCCCCTCATTTCTT CTTCTTCTCGCCTCATCTGGAGTCGAAGCAGGTGCCGGCACAGGATGGACTGTTTATCCCCCGC TCGCAGGCAATCTTGCCCACGCTGGACCTTCTGTTGACTTAACCATCTTCTCCCTCCACTTGGC CGGAGTGTCATCTATTTTAGGTGCAATTAATTTTATCACAACCATTATTAACATGAAACCCCCTGC CATCTCCCAATATCAAACACCCCTATTTGTGTGATCCGTCCTAATTACCGCAGTACTACTCCTTCT ATCCCTGCCCGTTCTTGCCGCCGGCATCACAATACTTCTAACAGACCGAAACCTAAACACAACC TTCTTTGACCCTGCCGGAGGAGGAGACCCCATCCTATACCAACACTTA
KU565864.1	*O. niloticus*	TTGAGCCGGAATAGTAGGAACTGCACTAAGCCTCCTAATTCGGGCAGAACTAAGCCAGCCCGG CTCTCTTCTCGGAGACGACCAAATCTATAATGTAATTGTTACAGCACATGCTTTCGTAATAATTTT CTTTATAGTAATACCAATTATGATTGGAGGCTTTGGAAACTGACTAGTACCCCTCATGATTGGTG CACCAGACATGGCCTTCCCTCGAATAAATAACATGAGCTTTTGACTTCTCCCCCCCTCATTTCTT CTTCTTCTCGCCTCATCTGGAGTCGAAGCAGGTGCCGGCACAGGATGGACTGTTTATCCCCCGC TCGCAGGCAATCTTGCCCACGCTGGACCTTCTGTTGACTTAACCATCTTCTCCCTCCACTTGGC CGGAGTGTCATCTATTTTAGGTGCAATTAATTTTATCACAACCATTATTAACATGAAACCCCCTGC CATCTCCCAATATCAAACACCCCTATTTGTGTGATCCGTCCTAATTACCGCAGTACTACTCCTTCT ATCCCTGCCCGTTCTTGCCGCCGGCATCACAATACTTCTAACAGACCGAAACCTAAACACAACC TTCTTTGACCCTGCCGGAGGAGGAGACCCCATCCTATACCAACACTTA
KU565865	*O. niloticus*	TTGAGCCGGAATAGTAGGAACTGCATTAAGCCTCCTAATTCGGGCAGAACTAAGCCAGCCCGG CTCTCTCCTCGGAGACGACCAGATTTATAATGTAATTGTTACAGCACATGCTTTCGTAATAATTTT CTTTATAGTAATGCCAATTATAATTGGAGGTTTTGGAAACTGACTAGTGCCACTAATGATTGGTG CACCAGACATGGCCTTCCCTCGAATAAATAACATGAGTTTTTGACTCCTCCCCCCCTCATTTCTC CTTCTCCTCGCCTCATCCGGGGTCGAAGCAGGGGCCGGTACAGGATGGACTGTTTATCCCCCAC TCGCAGGCAATCTCGCCCATGCTGGGCCTTCCGTTGACTTAACCATCTTCTCCCTCCACTTGGCC GGGGTGTCATCTATTTTAGGTGCAATTAATTTTATTACAACCATTATTAACATAAAACCCCCTGCC ATCTCCCAATATCAAACACCCCTCTTTGTATGATCCGTTCTAATTACCGCAGTACTACTCCTACTA TCCCTACCCGTTCTTGCCGCCGGCATCACAATACTTCTAACAGACCGAAACCTAAACACAACCT TCTTTGACCCTGCCGGAGGAGGAGACCCCATCCTTTACCAACACTTA
KU565867	*O. niloticus*	TTGAGCCGGAATAGTAGGAACTGCACTAAGCCTCCTAATTCGGGCAGAACTAAGCCAGCCCGG CTCTCTTCTCGGAGACGACCAAATCTATAATGTAATTGTTACAGCACATGCTTTCGTAATAATTTT CTTTATAGTAATACCAATTATGATTGGAGGCTTTGGAAACTGACTAGTACCCCTCATGATTGGTG CACCAGACATGGCCTTCCCTCGAATAAATAACATGAGCTTTTGACTTCTCCCCCCCTCATTTCTT CTTCTTCTCGCCTCATCTGGAGTCGAAGCAGGTGCCGGCACAGGATGGACTGTTTATCCCCCGC TCGCAGGCAATCTTGCCCACGCTGGACCTTCTGTTGACTTAACCATCTTCTCCCTCCACTTGGC CGGAGTGTCATCTATTTTAGGTGCAATTAATTTTATCACAACCATTATTAACATGAAACCCCCTGC CATCTCCCAATATCAAACACCCCTATTTGTGTGATCCGTCCTAATTACCGCAGTACTACTCCTTCT ATCCCTGCCCGTTCTTGCCGCCGGCATCACAATACTTCTAACAGACCGAAACCTAAACACAACC TTCTTTGACCCTGCCGGAGGAGGAGACCCCATCCTATACCAACACTTA
MF509596	*O. niloticus*	TTGAGCCGGAATAGTAGGAACTGCACTAAGCCTCCTAATTCGGGCAGAACTAAGCCAGCCCGG CTCTCTTCTCGGAGACGACCAAATCTATAATGTAATTGTTACAGCACATGCTTTCGTAATAATTTT CTTTATAGTAATACCAATTATGATTGGAGGCTTTGGAAACTGACTAGTACCCCTCATGATTGGTG CACCAGACATGGCCTTCCCTCGAATAAATAACATGAGCTTTTGACTTCTCCCCCCCTCATTTCTT CTTCTTCTCGCCTCATCTGGAGTCGAAGCAGGTGCCGGCACAGGATGGACTGTTTATCCCCCGC TCGCAGGCAATCTTGCCCACGCTGGACCTTCTGTTGACTTAACCATCTTCTCCCTCCACTTGGC CGGAGTGTCATCTATTTTAGGTGCAATTAATTTTATCACAACCATTATTAACATGAAACCCCCTGC CATCTCCCAATATCAAACACCCCTATTTGTGTGATCCGTCCTAATTACCGCAGTACTACTCCTTCT ATCCCTGCCCGTTCTTGCCGCCGGCATCACAATACTTCTAACAGACCGAAACCTAAACACAACC TTCTTTGACCCTGCCGGAGGAGGAGACCCCATCCTATACCAACACTTA
MF509597	*O. niloticus*	TTGAGCCGGAATAGTAGGAACTGCACTAAGCCTCCTAATTCGGGCAGAACTAAGCCAGCCCGG CTCTCTTCTCGGAGACGACCAAATCTATAATGTAATTGTTACAGCACATGCTTTCGTAATAATTTT CTTTATAGTAATACCAATTATGATTGGAGGCTTTGGAAACTGACTAGTACCCCTCATGATTGGTG CACCAGACATGGCCTTCCCTCGAATAAATAACATGAGCTTTTGACTTCTCCCCCCCTCATTTCTT CTTCTTCTCGCCTCATCTGGAGTCGAAGCAGGTGCCGGCACAGGATGGACTGTTTATCCCCCGC TCGCAGGCAATCTTGCCCACGCTGGACCTTCTGTTGACTTAACCATCTTCTCCCTCCACTTGGC CGGAGTGTCATCTATTTTAGGTGCAATTAATTTTATCACAACCATTATTAACATGAAACCCCCTGC CATCTCCCAATATCAAACACCCCTATTTGTGTGATCCGTCCTAATTACCGCAGTACTACTCCTTCT ATCCCTGCCCGTTCTTGCCGCCGGCATCACAATACTTCTAACAGACCGAAACCTAAACACAACC TTCTTTGACCCTGCCGGAGGAGGAGACCCCATCCTATACCAACACTTA
MG407409	*O. niloticus*	TTGAGCCGGAATAGTAGGAACTGCACTAAGCCTCCTAATTCGGGCAGAACTAAGCCAGCCCGG CTCTCTTCTCGGAGACGACCAAATCTATAATGTAATTGTTACAGCACATGCTTTCGTAATAATTTT CTTTATAGTAATACCAATTATGATTGGAGGCTTTGGAAACTGACTAGTACCCCTCATGATTGGTG CACCAGACATGGCCTTCCCTCGAATAAATAACATGAGCTTTTGACTTCTCCCCCCCTCATTTCTT CTTCTTCTCGCCTCATCTGGAGTCGAAGCAGGTGCCGGCACAGGATGGACTGTTTATCCCCCGC TCGCAGGCAATCTTGCCCACGCTGGACCTTCTGTTGACTTAACCATCTTCTCCCTCCACTTGGC CGGAGTGTCATCTATTTTAGGTGCAATTAATTTTATCACAACCATTATTAACATGAAACCCCCTGC CATCTCCCAATATCAAACACCCCTATTTGTGTGATCCGTCCTAATTACCGCAGTACTACTCCTTCT ATCCCTGCCCGTTCTTGCCGCCGGCATCACAATACTTCTAACAGACCGAAACCTAAACACAACC TTCTTTGACCCTGCCGGAGGAGGAGACCCCATCCTATACCAACACTTA

MG407410	*O. niloticus*	TTGAGCCGGAATAGTAGGAACTGCATTAAGCCTCCTAATTCGGGCAGAACTAAGCCAGCCCGG<br>CTCTCTCCTCGGAGACGACCAGATTTATAATGTAATTGTTACAGCACATGCTTTCGTAATAATTTT<br>CTTTATAGTAATGCCAATTATAATTGGAGGTTTTGGAAACTGACTAGTGCCACTAATGATTGGTG<br>CACCAGACATGGCCTTCCCTCGAATAAATAACATGAGTTTTTGACTCCTCCCCCCCTCATTTCTC<br>CTTCTCCTCGCCTCATCCGGGGTCGAAGCAGGGGCCGGTACAGGATGGACTGTTTATCCCCCAC<br>TCGCAGGCAATCTCGCCCATGCTGGGCCTTCCGTTGACTTAACCATCTTCTCCCTCCACTTGGCC<br>GGGGTGTCATCTATTTTAGGTGCAATTAATTTTATTACAACCATTATTAACATAAAACCCCCTGCC<br>ATCTCCCAATATCAAACACCCCTCTTTGTATGATCCGTTCTAATTACCGCAGTACTACTCCTACTA<br>TCCCTACCCGTTCTTGCCGCCGGCATCACAATACTTCTAACAGACCGAAACCTAAACACAACCT<br>TCTTTGACCCTGCCGGAGGAGGAGACCCCATCCTTTACCAACACTTA
MG407411	*O. niloticus*	TTGAGCCGGAATAGTAGGAACTGCACTAAGCCTCCTAATTCGGGCAGAACTAAGCCAGCCCGG<br>CTCTCTTCTCGGAGACGACCAAATCTATAATGTAATTGTTACAGCACATGCTTTCGTAATAATTTT<br>CTTTATAGTAATACCAATTATGATTGGAGGCTTTGGAAACTGACTAGTACCCCTCATGATTGGTG<br>CACCAGACATGGCCTTCCCTCGAATAAATAACATGAGCTTTTGACTTCTCCCCCCCTCATTTCTT<br>CTTCTTCTCGCCTCATCTGGAGTCGAAGCAGGTGCCGGCACAGGATGGACTGTTTATCCCCCGC<br>TCGCAGGCAATCTTGCCCACGCTGGACCTTCTGTTGACTTAACCATCTTCTCCCTCCACTTGGC<br>CGGAGTGTCATCTATTTTAGGTGCAATTAATTTTATCACAACCATTATTAACATGAAACCCCCTGC<br>CATCTCCCAATATCAAACACCCCTATTTGTGTGATCCGTCCTAATTACCGCAGTACTACTCCTTCT<br>ATCCCTGCCCGTTCTTGCCGCCGGCATCACAATACTTCTAACAGACCGAAACCTAAACACAACC<br>TTCTTTGACCCTGCCGGAGGAGGAGACCCCATCCTATACCAACACTTA
MG407412	*O. niloticus*	TTGAGCCGGAATAGTAGGAACCGCGCTAAGCCTCCTAATTCGGGCAGAACTAAGCCAGCCCGG<br>CTCTCTCCTCGGAGACGACCAGATTTATAATGTAATTGTTACAGCACATGCTTTTGTAATAATTTT<br>CTTTATAGTAATGCCAATTATGATTGGAGGCTTTGGAAACTGACTAGTACCACTCATGATTGGTG<br>CTCCAGATATGGCCTTCCCTCGAATGAACAACATGAGTTTCTGACTCCTCCCTCCCTCATTCCTC<br>CTCCTCCTCGCCTCATCTGGAGTCGAAGCAGGTGCCGGCACAGGGTGAACTGTTTACCCCCCG<br>CTCGCAGGCAATCTTGCCCATGCTGGGCCTTCTGTCGACTTAACCATCTTCTCCCTCCACTTGGC<br>CGGGGTGTCATCTATTCTAGGCGCAATTAATTTCATTACAACAATCATTAACATGAAACCCCCCG<br>CCATCTCTCAATATCAAACACCTCTATTTGTATGGTCCGTTCTAATTACCGCAGTATTACTTCTTC<br>TATCCCTACCCGTTCTTGCCGCCGGCATCACAATACTTCTCACAGACCGAAACCTAAACACAAC<br>CTTCTTTGATCCTGCCGGAGGAGGAGACCCCATCCTTTACCAACACTTA
MG407413	*O. niloticus*	TTGAGCCGGAATAGTAGGAACTGCATTAAGCCTCCTAATTCGGGCAGAACTAAGCCAGCCCGG<br>CTCTCTCCTCGGAGACGACCAGATTTATAATGTAATTGTTACAGCACATGCTTTCGTAATAATTTT<br>CTTTATAGTAATGCCAATTATAATTGGAGGTTTTGGAAACTGACTAGTGCCACTAATGATTGGTG<br>CACCAGACATGGCCTTCCCTCGAATAAATAACATGAGTTTTTGACTCCTCCCCCCCTCATTTCTC<br>CTTCTCCTCGCCTCATCCGGGGTCGAAGCAGGGGCCGGTACAGGATGGACTGTTTATCCCCCAC<br>TCGCAGGCAATCTCGCCCATGCTGGGCCTTCCGTTGACTTAACCATCTTCTCCCTCCACTTGGCC<br>GGGGTGTCATCTATTTTAGGTGCAATTAATTTTATTACAACCATTATTAACATAAAACCCCCTGCC<br>ATCTCCCAATATCAAACACCCCTCTTTGTATGATCCGTTCTAATTACCGCAGTACTACTCCTACTA<br>TCCCTACCCGTTCTTGCCGCCGGCATCACAATACTTCTAACAGACCGAAACCTAAACACAACCT<br>TCTTTGACCCTGCCGGAGGAGGAGACCCCATCCTTTACCAACACTTA
MG407414	*O. niloticus*	TTGAGCCGGAATAGTAGGAACTGCACTAAGCCTCCTAATTCGGGCAGAACTAAGCCAGCCCGG<br>CTCTCTTCTCGGAGACGACCAAATCTATAATGTAATTGTTACAGCACATGCTTTCGTAATAATTTT<br>CTTTATAGTAATACCAATTATGATTGGAGGCTTTGGAAACTGACTAGTACCCCTCATGATTGGTG<br>CACCAGACATGGCCTTCCCTCGAATAAATAACATGAGCTTTTGACTTCTCCCCCCCTCATTTCTT<br>CTTCTTCTCGCCTCATCTGGAGTCGAAGCAGGTGCCGGCACAGGATGGACTGTTTATCCCCCGC<br>TCGCAGGCAATCTTGCCCACGCTGGACCTTCTGTTGACTTAACCATCTTCTCCCTCCACTTGGC<br>CGGAGTGTCATCTATTTTAGGTGCAATTAATTTTATCACAACCATTATTAACATGAAACCCCCTGC<br>CATCTCCCAATATCAAACACCCCTATTTGTGTGATCCGTCCTAATTACCGCAGTACTACTCCTTCT<br>ATCCCTGCCCGTTCTTGCCGCCGGCATCACAATACTTCTAACAGACCGAAACCTAAACACAACC<br>TTCTTTGACCCTGCCGGAGGAGGAGACCCCATCCTATACCAACACTTA
MG407415	*O. niloticus*	TTGAGCCGGAATAGTAGGAACTGCACTAAGCCTCCTAATTCGGGCAGAACTAAGCCAGCCCGG<br>CTCTCTTCTCGGAGACGACCAAATCTATAATGTAATTGTTACAGCACATGCTTTCGTAATAATTTT<br>CTTTATAGTAATACCAATTATGATTGGAGGCTTTGGAAACTGACTAGTACCCCTCATGATTGGTG<br>CACCAGACATGGCCTTCCCTCGAATAAATAACATGAGCTTTTGACTTCTCCCCCCCTCATTTCTT<br>CTTCTTCTCGCCTCATCTGGAGTCGAAGCAGGTGCCGGCACAGGATGGACTGTTTATCCCCCGC<br>TCGCAGGCAATCTTGCCCACGCTGGACCTTCTGTTGACTTAACCATCTTCTCCCTCCACTTGGC<br>CGGAGTGTCATCTATTTTAGGTGCAATTAATTTTATCACAACCATTATTAACATGAAACCCCCTGC<br>CATCTCCCAATATCAAACACCCCTATTTGTGTGATCCGTCCTAATTACCGCAGTACTACTCCTTCT<br>ATCCCTGCCCGTTCTTGCCGCCGGCATCACAATACTTCTAACAGACCGAAACCTAAACACAACC<br>TTCTTTGACCCTGCCGGAGGAGGAGACCCCATCCTATACCAACACTTA
MG407416	*O. niloticus*	TTGAGCCGGAATAGTAGGAACTGCATTAAGCCTCCTAATTCGGGCAGAACTAAGCCAGCCCGG<br>CTCTCTCCTCGGAGACGACCAGATTTATAATGTAATTGTTACAGCACATGCTTTCGTAATAATTTT<br>CTTTATAGTAATGCCAATTATAATTGGAGGTTTTGGAAACTGACTAGTGCCACTAATGATTGGTG<br>CACCAGACATGGCCTTCCCTCGAATAAATAACATGAGTTTTTGACTCCTCCCCCCCTCATTTCTC<br>CTTCTCCTCGCCTCATCCGGGGTCGAAGCAGGGGCCGGTACAGGATGGACTGTTTATCCCCCAC<br>TCGCAGGCAATCTCGCCCATGCTGGGCCTTCCGTTGACTTAACCATCTTCTCCCTCCACTTGGCC<br>GGGGTGTCATCTATTTTAGGTGCAATTAATTTTATTACAACCATTATTAACATAAAACCCCCTGCC<br>ATCTCCCAATATCAAACACCCCTCTTTGTATGATCCGTTCTAATTACCGCAGTACTACTCCTACTA<br>TCCCTACCCGTTCTTGCCGCCGGCATCACAATACTTCTAACAGACCGAAACCTAAACACAACCT<br>TCTTTGACCCTGCCGGAGGAGGAGACCCCATCCTTTACCAACACTTA

MG407417	*O. niloticus*	TTGAGCCGGAATAGTAGGAACTGCACTAAGCCTCCTAATTCGGGCAGAACTAAGCCAGCCCGG<br>CTCTCTTCTCGGAGACGACCAAATCTATAATGTAATTGTTACAGCACATGCTTTCGTAATAATTTT<br>CTTTATAGTAATACCAATTATGATTGGAGGCTTTGGAAACTGACTAGTACCCCTCATGATTGGTG<br>CACCAGACATGGCCTTCCCTCGAATAAATAACATGAGCTTTTGACTTCTCCCCCCCTCATTTCTT<br>CTTCTTCTCGCCTCATCTGGAGTCGAAGCAGGTGCCGGCACAGGATGGACTGTTTATCCCCCGC<br>TCGCAGGCAATCTTGCCCACGCTGGACCTTCTGTTGACTTAACCATCTTCTCCCTCCACTTGGC<br>CGGAGTGTCATCTATTTTAGGTGCAATTAATTTTATCACAACCATTATTAACATGAAACCCCCTGC<br>CATCTCCCAATATCAAACACCCCTATTTGTGTGATCCGTCCTAATTACCGCAGTACTACTCCTTCT<br>ATCCCTGCCCGTTCTTGCCGCCGGCATCACAATACTTCTAACAGACCGAAACCTAAACACAACC<br>TTCTTTGACCCTGCCGGAGGAGGAGACCCCATCCTATACCAACACTTA
MG407418	*O. niloticus*	TTGAGCCGGAATAGTAGGAACTGCATTAAGCCTCCTAATTCGGGCAGAACTAAGCCAGCCCGG<br>CTCTCTCCTCGGAGACGACCAGATTTATAATGTAATTGTTACAGCACATGCTTTCGTAATAATTTT<br>CTTTATAGTAATGCCAATTATAATTGGAGGTTTTGGAAACTGACTAGTGCCACTAATGATTGGTG<br>CACCAGACATGGCCTTCCCTCGAATAAATAACATGAGTTTTTGACTCCTCCCCCCCTCATTTCTC<br>CTTCTCCTCGCCTCATCCGGGGTCGAAGCAGGGGCCGGTACAGGATGGACTGTTTATCCCCCAC<br>TCGCAGGCAATCTCGCCCATGCTGGGCCTTCCGTTGACTTAACCATCTTCTCCCTCCACTTGGCC<br>GGGGTGTCATCTATTTTAGGTGCAATTAATTTTATTACAACCATTATTAACATAAAACCCCCTGCC<br>ATCTCCCAATATCAAACACCCCTCTTTGTATGATCCGTTCTAATTACCGCAGTACTACTCCTACTA<br>TCCCTACCCGTTCTTGCCGCCGGCATCACAATACTTCTAACAGACCGAAACCTAAACACAACCT<br>TCTTTGACCCTGCCGGAGGAGGAGACCCCATCCTTTACCAACACTTA
MG438454	*O. niloticus*	TTGAGCCGGAATAGTAGGAACTGCACTAAGCCTCCTAATTCGGGCAGAACTAAGCCAGCCCGG<br>CTCTCTTCTCGGAGACGACCAAATCTATAATGTAATTGTTACAGCACATGCTTTCGTAATAATTTT<br>CTTTATAGTAATACCAATTATGATTGGAGGCTTTGGAAACTGACTAGTACCCCTCATGATTGGTG<br>CACCAGACATGGCCTTCCCTCGAATAAATAACATGAGCTTTTGACTTCTCCCCCCCTCATTTCTT<br>CTTCTTCTCGCCTCATCTGGAGTCGAAGCAGGTGCCGGCACAGGATGGACTGTTTATCCCCCGC<br>TCGCAGGCAATCTTGCCCACGCTGGACCTTCTGTTGACTTAACCATCTTCTCCCTCCACTTGGC<br>CGGAGTGTCATCTATTTTAGGTGCAATTAATTTTATCACAACCATTATTAACATGAAACCCCCTGC<br>CATCTCCCAATATCAAACACCCCTATTTGTGTGATCCGTCCTAATTACCGCAGTACTACTCCTTCT<br>ATCCCTGCCCGTTCTTGCCGCCGGCATCACAATACTTCTAACAGACCGAAACCTAAACACAACC<br>TTCTTTGACCCTGCCGGAGGAGGAGACCCCATCCTATACCAACACTTA
MG438455	*O. niloticus*	TTGAGCCGGAATAGTAGGAACTGCACTAAGCCTCCTAATTCGGGCAGAACTAAGCCAGCCCGG<br>CTCTCTTCTCGGAGACGACCAAATCTATAATGTAATTGTTACAGCACATGCTTTCGTAATAATTTT<br>CTTTATAGTAATACCAATTATGATTGGAGGCTTTGGAAACTGACTAGTACCCCTCATGATTGGTG<br>CACCAGACATGGCCTTCCCTCGAATAAATAACATGAGCTTTTGACTTCTCCCCCCCTCATTTCTT<br>CTTCTTCTCGCCTCATCTGGAGTCGAAGCAGGTGCCGGCACAGGATGGACTGTTTATCCCCCGC<br>TCGCAGGCAATCTTGCCCACGCTGGACCTTCTGTTGACTTAACCATCTTCTCCCTCCACTTGGC<br>CGGAGTGTCATCTATTTTAGGTGCAATTAATTTTATCACAACCATTATTAACATGAAACCCCCTGC<br>CATCTCCCAATATCAAACACCCCTATTTGTGTGATCCGTCCTAATTACCGCAGTACTACTCCTTCT<br>ATCCCTGCCCGTTCTTGCCGCCGGCATCACAATACTTCTAACAGACCGAAACCTAAACACAACC<br>TTCTTTGACCCTGCCGGAGGAGGAGACCCCATCCTATACCAACACTTA
MG438456	*O. niloticus*	TTGAGCCGGAATAGTAGGAACTGCACTAAGCCTCCTAATTCGGGCAGAACTAAGCCAGCCCGG<br>CTCTCTTCTCGGAGACGACCAAATCTATAATGTAATTGTTACAGCACATGCTTTCGTAATAATTTT<br>CTTTATAGTAATACCAATTATGATTGGAGGCTTTGGAAACTGACTAGTACCCCTCATGATTGGTG<br>CACCAGACATGGCCTTCCCTCGAATAAATAACATGAGCTTTTGACTTCTCCCCCCCTCATTTCTT<br>CTTCTTCTCGCCTCATCTGGAGTCGAAGCAGGTGCCGGCACAGGATGGACTGTTTATCCCCCGC<br>TCGCAGGCAATCTTGCCCACGCTGGACCTTCTGTTGACTTAACCATCTTCTCCCTCCACTTGGC<br>CGGAGTGTCATCTATTTTAGGTGCAATTAATTTTATCACAACCATTATTAACATGAAACCCCCTGC<br>CATCTCCCAATATCAAACACCCCTATTTGTGTGATCCGTCCTAATTACCGCAGTACTACTCCTTCT<br>ATCCCTGCCCGTTCTTGCCGCCGGCATCACAATACTTCTAACAGACCGAAACCTAAACACAACC<br>TTCTTTGACCCTGCCGGAGGAGGAGACCCCATCCTATACCAACACTTA
MG438457	*O. niloticus*	TTGAGCCGGAATAGTAGGAACTGCACTAAGCCTCCTAATTCGGGCAGAACTAAGCCAGCCCGG<br>CTCTCTTCTCGGAGACGACCAAATCTATAATGTAATTGTTACAGCACATGCTTTCGTAATAATTTT<br>CTTTATAGTAATACCAATTATGATTGGAGGCTTTGGAAACTGACTAGTACCCCTCATGATTGGTG<br>CACCAGACATGGCCTTCCCTCGAATAAATAACATGAGCTTTTGACTTCTCCCCCCCTCATTTCTT<br>CTTCTTCTCGCCTCATCTGGAGTCGAAGCAGGTGCCGGCACAGGATGGACTGTTTATCCCCCGC<br>TCGCAGGCAATCTTGCCCACGCTGGACCTTCTGTTGACTTAACCATCTTCTCCCTCCACTTGGC<br>CGGAGTGTCATCTATTTTAGGTGCAATTAATTTTATCACAACCATTATTAACATGAAACCCCCTGC<br>CATCTCCCAATATCAAACACCCCTATTTGTGTGATCCGTCCTAATTACCGCAGTACTACTCCTTCT<br>ATCCCTGCCCGTTCTTGCCGCCGGCATCACAATACTTCTAACAGACCGAAACCTAAACACAACC<br>TTCTTTGACCCTGCCGGAGGAGGAGACCCCATCCTATACCAACACTTA
MH515186	*O. niloticus*	TTGAGCCGGAATAGTAGGAACCGCGCTAAGCCTCCTAATTCGGGCAGAACTAAGCCAACCCGG<br>CTCTCTCCTCGGAGACGACCAGATTTATAATGTAATTGTTACAGCACATGCTTTTGTAATAATTTT<br>CTTTATAGTAATGCCAATTATGATTGGAGGCTTTGGAAACTGACTAGTACCACTCATGATTGGTG<br>CCCCAGATATGGCCTTCCCTCGAATGAACAACATGAGTTTCTGACTCCTCCCTCCCTCATTCCTC<br>CTCCTCCTCGCCTCATCTGGAGTCGAAGCAGGTGCCGGCACAGGGTGAACTGTTTACCCCCCG<br>CTCGCAGGCAATCTTGCCCATGCTGGGCCTTCTGTTGACTTAACCATCTTCTCCCTCCACTTGGC<br>CGGAGTGTCATCTATTTTAGGTGCAATTAATTTTATCACAACCATTATTAACATGAAACCCCCTGC<br>CATCTCCCAATATCAAACACCCCTATTTGTGTGATCCGTCCTAATTACCGCAGTACTACTCCTTCT<br>ATCCCTGCCCGTTCTTGCCGCCGGCATCACAATACTTCTAACAGACCGAAACCTAAACACAACC<br>TTCTTTGACCCTGCCGGAGGAGGAGACCCCATCCTATACCAACACTTA

MH515227	*O. aureus*	TTGAGCCGGAATAGTAGGAACTGCACTAAGCCTCCTAATTCGGGCAGAACTAAGCCAGCCCGG<br>CTCTCTCCTCGGAGACGACCAGATTTATAATGTAATTGTTACAGCACATGCTTTCGTAATAATTTT<br>CTTTATAGTAATGCCAATTATAATTGGAGGTTTTGGAAACTGACTAGTGCCACTAATGATTGGTG<br>CACCAGACATGGCCTTCCCTCGAATAAATAACATGAGTTTTTGACTCCTCCCCCCCTCATTTCTC<br>CTTCTCCTCGCCTCATCCGGGGTCGAAGCAGGGGCCGGTACAGGATGGACTGTTTATCCCCCAC<br>TCGCAGGCAATCTCGCCCATGCTGGGCCTTCCGTTGACTTAACCATCTTCTCCCTCCACTTGGCC<br>GGGGTGTCATCTATTTTAGGTGCAATTAATTTTATTACAACCATTATTAACATAAAACCCCCTGCC<br>ATCTCCCAATATCAAACACCCCTCTTTGTATGATCCGTTCTAATTACCGCAGTACTACTCCTACTA<br>TCCCTACCCGTTCTTGCCGCCGGCATCACAATACTTCTAACAGACCGAAACCTAAACACAACCT<br>TCTTTGACCCTGCCGGAGGAGGAGACCCCATCCTTTACCAACACTTA
MH515228	*O. aureus*	TTGAGCCGGAATAGTAGGAACTGCACTAAGCCTCCTAATTCGGGCAGAACTAAGCCAGCCCGG<br>CTCTCTTCTCGGAGACGACCAAATCTATAATGTAATTGTTACAGCACATGCTTTCGTAATAATTTT<br>CTTTATAGTAATACCAATTATGATTGGAGGCTTTGGAAACTGACTAGTACCCCTCATGATTGGTG<br>CACCAGACATGGCCTTCCCTCGAATAAATAACATGAGCTTTTGACTTCTCCCCCCCTCATTTCTT<br>CTTCTTCTCGCCTCATCTGGAGTCGAAGCAGGTGCCGGCACAGGATGGACTGTTTATCCCCCGC<br>TCGCAGGCAATCTTGCCCACGCTGGACCTTCTGTTGACTTAACCATCTTCTCCCTCCACTTGGC<br>CGGAGTGTCATCTATTTTAGGTGCAATTAATTTTATCACAACCATTATTAACATGAAACCCCCTGC<br>CATCTCCCAATATCAAACACCCCTATTTGTGTGATCCGTCCTAATTACCGCAGTACTACTCCTTCT<br>ATCCCTGCCCGTTCTTGCCGCCGGCATCACAATACTTCTAACAGACCGAAACCTAAACACAACC<br>TTCTTTGACCCTGCCGGAGGAGGAGACCCCATCCTATACCAACACTTA
MH515229	*O. aureus*	TTGAGCCGGAATAGTAGGAACCGCGCTAAGCCTCCTAATTCGGGCAGAACTAAGCCAGCCCGG<br>CTCTCTCCTCGGAGACGACCAGATTTATAATGTAATTGTTACAGCACATGCTTTTGTAATAATTTT<br>CTTTATAGTAATGCCAATTATGATTGGAGGCTTTGGAAACTGACTAGTACCACTCATGATTGGTG<br>CCCCAGATATGGCCTTCCCTCGAATGAACAACATGAGTTTCTGACTCCTCCCTCCCTCATTCCTC<br>CTCCTCCTCGCCTCATCTGGAGTCGAAGCAGGTGCCGGCACAGGGTGAACTGTTTACCCCCCG<br>CTCGCAGGCAATCTTGCCCATGCTGGGCCTTCTGTCGACTTAACCATCTTCTCCCTCCACTTGGC<br>CGGGGTGTCATCTATTCTAGGCGCAATTAATTTCATTACAACAATCATTAACATGAAACCCCCCG<br>CCATCTCTCAATATCAAACACCCCTATTTGTATGGTCCGTTCTAATTACCGCAGTATTACTTCTTC<br>TATCCCTACCCGTTCTTGCCGCCGGCATCACAATACTTCTCACAGACCGAAACCTAAACACAAC<br>CTTCTTTGATCCTGCCGGAGGAGGAGACCCCATCCTTTACCAACACTTA
MH515230	*O. aureus*	TTGAGCCGGAATAGTAGGAACCGCGCTAAGCCTCCTAATTCGGGCAGAACTAAGCCAGCCCGG<br>CTCTCTCCTCGGAGACGACCAGATTTATAATGTAATTGTTACAGCACATGCTTTTGTAATAATTTT<br>CTTTATAGTAATGCCAATTATGATTGGAGGCTTTGGAAACTGACTAGTACCACTCATGATTGGTG<br>CCCCAGATATGGCCTTCCCTCGAATGAACAACATGAGTTTCTGACTCCTCCCTCCCTCATTCCTC<br>CTCCTCCTCGCCTCATCTGGAGTCGAAGCAGGTGCCGGCACAGGGTGAACTGTTTACCCCCCG<br>CTCGCAGGCAATCTTGCCCATGCTGGGCCTTCTGTCGACTTAACCATCTTCTCCCTCCACTTGGC<br>CGGGGTGTCATCTATTCTAGGCGCAATTAATTTCATTACAACAATCATTAACATGAAACCCCCCG<br>CCATCTCTCAATATCAAACACCCCTATTTGTATGGTCCGTTCTAATTACCGCAGTATTACTTCTTC<br>TATCCCTACCCGTTCTTGCCGCCGGCATCACAATACTTCTCACAGACCGAAACCTAAACACAAC<br>CTTCTTTGATCCTGCCGGAGGAGGAGACCCCATCCTTTACCAACACTTA
MH515231	*O. aureus*	TTGAGCCGGAATAGTAGGAACCGCGCTAAGCCTCCTAATTCGGGCAGAACTAAGCCAGCCCGG<br>CTCTCTCCTCGGAGACGACCAGATTTATAATGTAATTGTTACAGCACATGCTTTTGTAATAATTTT<br>CTTTATAGTAATGCCAATTATGATTGGAGGCTTTGGAAACTGACTAGTACCACTCATGATTGGTG<br>CCCCAGATATGGCCTTCCCTCGAATGAACAACATGAGTTTCTGACTCCTCCCTCCCTCATTCCTC<br>CTCCTCCTCGCCTCATCTGGAGTCGAAGCAGGTGCCGGCACAGGGTGAACTGTTTACCCCCCG<br>CTCGCAGGCAATCTTGCCCATGCTGGGCCTTCTGTCGACTTAACCATCTTCTCCCTCCACTTGGC<br>CGGGGTGTCATCTATTCTAGGCGCAATTAATTTCATTACAACAATCATTAACATGAAACCCCCCG<br>CCATCTCTCAATATCAAACACCCCTATTTGTATGGTCCGTTCTAATTACCGCAGTATTACTTCTTC<br>TATCCCTACCCGTTCTTGCCGCCGGCATCACAATACTTCTCACAGACCGAAACCTATACACAAC<br>CTTCTTTGATCCTGCCGGAGGAGGAGACCCCATCCTTTACCAACACTTA
MK074525	*O. niloticus*	TTGAGCCGGAATAGTAGGAACCGCGCTAAGCCTCCTAATTCGGGCAGAACTAAGCCAGCCCGG<br>CTCTCTCCTCGGAGACGACCAGATTTATAATGTAATTGTTACAGCACATGCTTTTGTAATAATTTT<br>CTTTATAGTAATGCCAATTATGATTGGAGGCTTTGGAAACTGACTAGTACCACTCATGATTGGTG<br>CCCCAGATATGGCCTTCCCTCGAATGAACAACATGAGTTTCTGACTCCTCCCTCCCTCATTCCTC<br>CTCCTCCTCGCCTCATCTGGAGTCGAAGCAGGTGCCGGCACAGGGTGAACTGTTTACCCCCCG<br>CTCGCAGGCAATCTTGCCCATGCTGGGCCTTCTGTCGACTTAACCATCTTCTCCCTCCACTTGGC<br>CGGGGTGTCATCTATTCTAGGCGCAATTAATTTCATTACAACAATCATTAACATGAAACCCCCCG<br>CCATCTCTCAATATCAAACACCCCTATTTGTATGGTCCGTTCTAATTACCGCAGTATTACTTCTTCT<br>ATCCCTACCCGTTCTTGCCGCCGGCATCACAATACTTCTCACAGACCGAAACCTAAACACAACC<br>TTCTTTGATCCTGCCGGAGGAGGAGACCCCATCCTTTACCAACACTTA
MK074526	*O. niloticus*	TTGAGCCGGAATAGTAGGAACCGCGCTAAGCCTCCTAATTCGGGCAGAACTAAGCCAGCCCGG<br>CTCTCTCCTCGGAGACGACCAGATTTATAATGTAATTGTTACAGCACATGCTTTTGTAATAATTTT<br>CTTTATAGTAATGCCAATTATGATTGGAGGCTTTGGAAACTGACTAGTACCACTCATGATTGGTG<br>CCCCAGATATGGCCTTCCCTCGAATGAACAACATGAGTTTCTGACTCCTCCCTCCCTCATTCCTC<br>CTCCTCCTCGCCTCATCTGGAGTCGAAGCAGGTGCCGGCACAGGGTGAACTGTTTACCCCCCG<br>CTCGCAGGCAATCTTGCCCATGCTGGGCCTTCTGTCGACTTAACCATCTTCTCCCTCCACTTGGC<br>CGGGGTGTCATCTATTCTAGGCGCAATTAATTTCATTACAACAATCATTAACATGAAACCCCCCG<br>CCATCTCTCAATATCAAACACCCCTATTTGTATGGTCCGTTCTAATTACCGCAGTATTACTTCTTC<br>TATCCCTACCCGTTCTTGCCGCCGGCATCACAATACTTCTCACAGACCGAAACCTAAACACAAC<br>CTTCTTTGATCCTGCCGGAGGAGGAGACCCCATCCTTTACCAACACTTA

MK074527	*O. niloticus*	TTGAGCCGGAATAGTAGGAACCGCGCTAAGCCTCCTAATTCGGGCAGAACTAAGCCAGCCCGG<br>CTCTCTCCTCGGAGACGACCAGATTTATAATGTAATTGTTACAGCACATGCTTTTGTAATAATTTT<br>CTTTATAGTAATGCCAATTATGATTGGAGGCTTTGGAAACTGACTAGTACCACTCATGATTGGTG<br>CCCCAGATATGGCCTTCCCTCGAATGAACAACATGAGTTTCTGACTCCTCCCTCCCTCATTCCTC<br>CTCCTCCTCGCCTCATCTGGAGTCGAAGCAGGTGCCGGCACAGGGTGAACTGTTTACCCCCCG<br>CTCGCAGGCAATCTTGCCCATGCTGGGCCTTCTGTCGACTTAACCATCTTCTCCCTCCACTTGGC<br>CGGGGTGTCATCTATTCTAGGCGCAATTAATTTCATTACAACAATCATTAACATGAAACCCCCCG<br>CCATCTCTCAATATCAAACACCCCTATTTGTATGGTCCGTTCTAATTACCGCAGTATTACTTCTTC<br>TATCCCTACCCGTTCTTGCCGCCGGCATCACAATACTTCTCACAGACCGAAACCTAAACACAAC<br>CTTCTTTGATCCTGCCGGAGGAGGAGACCCCATCCTTTACCAACACTTA
MK074528	*O. niloticus*	TTGAGCCGGAATAGTAGGAACCGCGCTAAGCCTCCTAATTCGGGCAGAACTAAGCCAGCCCGG<br>CTCTCTCCTCGGAGACGACCAGATTTATAATGTAATTGTTACAGCACATGCTTTTGTAATAATTTT<br>CTTTATAGTAATGCCAATTATGATTGGAGGCTTTGGAAACTGACTAGTACCACTCATGATTGGTG<br>CCCCAGATATGGCCTTCCCTCGAATGAACAACATGAGTTTCTGACTCCTCCCTCCCTCATTCCTC<br>CTCCTCCTCGCCTCATCTGGAGTCGAAGCAGGTGCCGGCACAGGGTGAACTGTTTACCCCCCG<br>CTCGCAGGCAATCTTGCCCATGCTGGGCCTTCTGTCGACTTAACCATCTTCTCCCTCCACTTGGC<br>CGGGGTGTCATCTATTCTAGGCGCAATTAATTTCATTACAACAATCATTAACATGAAACCCCCCG<br>CCATCTCTCAATATCAAACACCCCTATTTGTATGGTCCGTTCTAATTACCGCAGTATTACTTCTTC<br>TATCCCTACCCGTTCTTGCCGCCGGCATCACAATACTTCTCACAGACCGAAACCTAAACACAAC<br>CTTCTTTGATCCTGCCGGAGGAGGAGACCCCATCCTTTACCAACACTTA
MK074529	*O. niloticus*	TTGAGCCGGAATAGTAGGAACCGCGCTAAGCCTCCTAATTCGGGCAGAACTAAGCCAGCCCGG<br>CTCTCTCCTCGGAGACGACCAGATTTATAATGTAATTGTTACAGCACATGCTTTTGTAATAATTTT<br>CTTTATAGTAATGCCAATTATGATTGGAGGCTTTGGAAACTGACTAGTACCACTCATGATTGGTG<br>CCCCAGATATGGCCTTCCCTCGAATGAACAACATGAGTTTCTGACTCCTCCCTCCCTCATTCCTC<br>CTCCTCCTCGCCTCATCTGGAGTCGAAGCAGGTGCCGGCACAGGGTGAACTGTTTACCCCCCG<br>CTCGCAGGCAATCTTGCCCATGCTGGGCCTTCTGTCGACTTAACCATCTTCTCCCTCCACTTGGC<br>CGGGGTGTCATCTATTCTAGGCGCAATTAATTTCATTACAACAATCATTAACATGAAACCCCCCG<br>CCATCTCTCAATATCAAACACCCCTATTTGTATGGTCCGTTCTAATTACCGCAGTATTACTTCTTC<br>TATCCCTACCCGTTCTTGCCGCCGGCATCACAATACTTCTCACAGACCGAAACCTAAACACAAC<br>CTTCTTTGATCCTGCCGGAGGAGGAGACCCCATCCTTTACCAACACTTA
MK074530	*O. niloticus*	TTGAGCCGGAATAGTAGGAACCGCGCTAAGCCTCCTAATTCGGGCAGAACTAAGCCAGCCCGG<br>CTCTCTCCTCGGAGACGACCAGATTTATAATGTAATTGTTACAGCACATGCTTTTGTAATAATTTT<br>CTTTATAGTAATGCCAATTATGATTGGAGGCTTTGGAAACTGACTAGTACCACTCATGATTGGTG<br>CCCCAGATATGGCCTTCCCTCGAATGAACAACATGAGTTTCTGACTCCTCCCTCCCTCATTCCTC<br>CTCCTCCTCGCCTCATCTGGAGTCGAAGCAGGTGCCGGCACAGGGTGAACTGTTTACCCCCCG<br>CTCGCAGGCAATCTTGCCCATGCTGGGCCTTCTGTCGACTTAACCATCTTCTCCCTCCACTTGGC<br>CGGGGTGTCATCTATTCTAGGCGCAATTAATTTCATTACAACAATCATTAACATGAAACCCCCCG<br>CCATCTCTCAATATCAAACACCCCTATTTGTATGGTCCGTTCTAATTACCGCAGTATTACTTCTTC<br>TATCCCTACCCGTTCTTGCCGCCGGCATCACAATACTTCTCACAGACCGAAACCTAAACACAAC<br>CTTCTTTGATCCTGCCGGAGGAGGAGACCCCATCCTTTACCAACACTTA
MK074531	*O. niloticus*	TTGAGCCGGAATAGTAGGAACCGCGCTAAGCCTCCTAATTCGGGCAGAACTAAGCCAGCCCGG<br>CTCTCTCCTCGGAGACGACCAGATTTATAATGTAATTGTTACAGCACATGCTTTTGTAATAATTTT<br>CTTTATAGTAATGCCAATTATGATTGGAGGCTTTGGAAACTGACTAGTACCACTCATGATTGGTG<br>CCCCAGATATGGCCTTCCCTCGAATGAACAACATGAGTTTCTGACTCCTCCCTCCCTCATTCCTC<br>CTCCTCCTCGCCTCATCTGGAGTCGAAGCAGGTGCCGGCACAGGGTGAACTGTTTACCCCCCG<br>CTCGCAGGCAATCTTGCCCATGCTGGGCCTTCTGTCGACTTAACCATCTTCTCCCTCCACTTGGC<br>CGGGGTGTCATCTATTCTAGGCGCAATTAATTTCATTACAACAATCATTAACATGAAACCCCCCG<br>CCATCTCTCAATATCAAACACCCCTATTTGTATGGTCCGTTCTAATTACCGCAGTATTACTTCTTC<br>TATCCCTACCCGTTCTTGCCGCCGGCATCACAATACTTCTCACAGACCGAAACCTAAACACAAC<br>CTTCTTTGATCCTGCCGGAGGAGGAGACCCCATCCTTTACCAACACTTA
MK074532	*O. niloticus*	TTGAGCCGGAATAGTAGGAACCGCGCTAAGCCTCCTAATTCGGGCAGAACTAAGCCAGCCCGG<br>CTCTCTCCTCGGAGACGACCAGATTTATAATGTAATTGTTACAGCACATGCTTTTGTAATAATTTT<br>CTTTATAGTAATGCCAATTATGATTGGAGGCTTTGGAAACTGACTAGTACCACTCATGATTGGTG<br>CCCCAGATATGGCCTTCCCTCGAATGAACAACATGAGTTTCTGACTCCTCCCTCCCTCATTCCTC<br>CTCCTCCTCGCCTCATCTGGAGTCGAAGCAGGTGCCGGCACAGGGTGAACTGTTTACCCCCCG<br>CTCGCAGGCAATCTTGCCCATGCTGGGCCTTCTGTCGACTTAACCATCTTCTCCCTCCACTTGGC<br>CGGGGTGTCATCTATTCTAGGCGCAATTAATTTCATTACAACAATCATTAACATGAAACCCCCCG<br>CCATCTCTCAATATCAAACACCCCTATTTGTATGGTCCGTTCTAATTACCGCAGTATTACTTCTTCT<br>ATCCCTACCCGTTCTTGCCGCCGGCATCACAATACTTCTCACAGACCGAAACCTAAACACAACC<br>TTCTTTGATCCTGCCGGAGGAGGAGACCCCATCCTTTACCAACACTTA
MK074533	*O. niloticus*	TTGAGCCGGAATAGTAGGAACCGCGCTAAGCCTCCTAATTCGGGCAGAACTAAGCCAGCCCGG<br>CTCTCTCCTCGGAGACGACCAGATTTATAATGTAATTGTTACAGCACATGCTTTTGTAATAATTTT<br>CTTTATAGTAATGCCAATTATGATTGGAGGCTTTGGAAACTGACTAGTACCACTCATGATTGGTG<br>CCCCAGATATGGCCTTCCCTCGAATGAACAACATGAGTTTCTGACTCCTCCCTCCCTCATTCCTC<br>CTCCTCCTCGCCTCATCTGGAGTCGAAGCAGGTGCCGGCACAGGGTGAACTGTTTACCCCCCG<br>CTCGCAGGCAATCTTGCCCATGCTGGGCCTTCTGTCGACTTAACCATCTTCTCCCTCCACTTGGC<br>CGGGGTGTCATCTATTCTAGGCGCAATTAATTTCATTACAACAATCATTAACATGAAACCCCCCG<br>CCATCTCTCAATATCAAACACCCCTATTTGTATGGTCCGTTCTAATTACCGCAGTATTACTTCTTC<br>TATCCCTACCCGTTCTTGCCGCCGGCATCACAATACTTCTCACAGACCGAAACCTAAACACAAC<br>CTTCTTTGATCCTGCCGGAGGAGGAGACCCCATCCTTTACCAACACTTA

MK130700	*O. niloticus*	TTGAGCCGGAATAGTAGGAACTGCACTAAGCCTCCTAATTCGGGCAGAACTAAGCCAGCCCGG<br>CTCTCTTCTCGGAGACGACCAAATCTATAATGTAATTGTTACAGCACATGCTTTCGTAATAATTTT<br>CTTTATAGTAATACCAATTATGATTGGAGGCTTTGGAAACTGACTAGTACCCCTCATGATTGGTG<br>CACCAGACATGGCCTTCCCTCGAATAAATAACATGAGCTTTTGACTTCTCCCCCCCTCATTTCTT<br>CTTCTTCTCGCCTCATCTGGAGTCGAAGCAGGTGCCGGCACAGGATGGACTGTTTATCCCCCGC<br>TCGCAGGCAATCTTGCCCACGCTGGACCTTCTGTTGACTTAACCATCTTCTCCCTCCACTTGGC<br>CGGAGTGTCATCTATTTTAGGTGCAATTAATTTTATCACAACCATTATTAACATGAAACCCCCTGC<br>CATCTCCCAATATCAAACACCCCTATTTGTGTGATCCGTCCTAATTACCGCAGTACTACTCCTTCT<br>ATCCCTGCCCGTTCTTGCCGCCGGCATCACAATACTTCTAACAGACCGAAACCTAAACACAACC<br>TTCTTTGACCCTGCCGGAGGAGGAGACCCCATCCTATACCAACACTTA
MK130701	*O. aureus*	TTGAGCCGGAATAGTAGGAACCGCGCTAAGCCTCCTAATTCGGGCAGAACTAAGCCAGCCCGG<br>CTCTCTCCTCGGAGACGACCAGATTTATAATGTAATTGTTACAGCACATGCTTTTGTAATAATTTT<br>CTTTATAGTAATGCCAATTATGATTGGAGGCTTTGGAAACTGACTAGTACCACTCATGATTGGTG<br>CCCCAGATATGGCCTTCCCTCGAATGAACAACATGAGTTTCTGACTCCTCCCTCCCTCATTCCTC<br>CTCCTCCTCGCCTCATCTGGAGTCGAAGCAGGTGCCGGCACAGGGTGAACTGTTTACCCCCCG<br>CTCGCAGGCAATCTTGCCCATGCTGGGCCTTCTGTCGACTTAACCATCTTCTCCCTCCACTTGGC<br>CGGGGTGTCATCTATTCTAGGCGCAATTAATTTCATTACAACAATCATTAACATGAAACCCCCCG<br>CCATCTCTCAATATCAAACACCCCTATTTGTATGGTCCGTTCTAATTACCGCAGTATTACTTCTTC<br>TATCCCTACCCGTTCTTGCCGCCGGCATCACAATACTTCTCACAGACCGAAACCTAAACACAAC<br>CTTCTTTGATCCTGCCGGAGGAGGAGACCCCATCCTTTACCAACACTTA
MK130702	*O. niloticus*	TTGAGCCGGAATAGTAGGAACTGCACTAAGCCTCCTAATTCGGGCAGAACTAAGCCAGCCCGG<br>CTCTCTTCTCGGAGACGACCAAATCTATAATGTAATTGTTACAGCACATGCTTTCGTAATAATTTT<br>CTTTATAGTAATACCAATTATGATTGGAGGCTTTGGAAACTGACTAGTACCCCTCATGATTGGTG<br>CACCAGACATGGCCTTCCCTCGAATAAATAACATGAGCTTTTGACTTCTCCCCCCCTCATTTCTT<br>CTTCTTCTCGCCTCATCTGGAGTCGAAGCAGGTGCCGGCACAGGATGGACTGTTTATCCCCCGC<br>TCGCAGGCAATCTTGCCCACGCTGGACCTTCTGTTGACTTAACCATCTTCTCCCTCCACTTGGC<br>CGGAGTGTCATCTATTTTAGGTGCAATTAATTTTATCACAACCATTATTAACATGAAACCCCCTGC<br>CATCTCCCAATATCAAACACCCCTATTTGTGTGATCCGTCCTAATTACCGCAGTACTACTCCTTCT<br>ATCCCTGCCCGTTCTTGCCGCCGGCATCACAATACTTCTAACAGACCGAAACCTAAACACAACC<br>TTCTTTGACCCTGCCGGAGGAGGAGACCCCATCCTATACCAACACTTA
MK130703	*O. aureus*	TTGAGCCGGAATAGTAGGAACCGCGCTAAGCCTCCTAATTCGGGCAGAACTAAGCCAGCCCGG<br>CTCTCTCCTCGGAGACGACCAGATTTATAATGTAATTGTTACAGCACATGCTTTTGTAATAATTTT<br>CTTTATAGTAATGCCAATTATGATTGGAGGCTTTGGAAACTGACTAGTACCACTCATGATTGGTG<br>CCCCAGATATGGCCTTCCCTCGAATGAACAACATGAGTTTCTGACTCCTCCCTCCCTCATTCCTC<br>CTCCTCCTCGCCTCATCTGGAGTCGAAGCAGGTGCCGGCACAGGGTGAACTGTTTACCCCCCG<br>CTCGCAGGCAATCTTGCCCATGCTGGGCCTTCTGTCGACTTAACCATCTTCTCCCTCCACTTGGC<br>CGGGGTGTCATCTATTCTAGGCGCAATTAATTTCATTACAACAATCATTAACATGAAACCCCCCG<br>CCATCTCTCAATATCAAACACCCCTATTTGTATGGTCCGTTCTAATTACCGCAGTATTACTTCTTC<br>TATCCCTACCCGTTCTTGCCGCCGGCATCACAATACTTCTCACAGACCGAAACCTAAACACAAC<br>CTTCTTTGATCCTGCCGGAGGAGGAGACCCCATCCTTTACCAACACTTA
MK130704	*O. aureus*	TTGAGCCGGAATAGTAGGAACCGCGCTAAGCCTCCTAATTCGGGCAGAACTAAGCCAGCCCGG<br>CTCTCTCCTCGGAGACGACCAGATTTATAATGTAATTGTTACAGCACATGCTTTTGTAATAATTTT<br>CTTTATAGTAATGCCAATTATGATTGGAGGCTTTGGAAACTGACTAGTACCACTCATGATTGGTG<br>CCCCAGATATGGCCTTCCCTCGAATGAACAACATGAGTTTCTGACTCCTCCCTCCCTCATTCCTC<br>CTCCTCCTCGCCTCATCTGGAGTCGAAGCAGGTGCCGGCACAGGGTGAACTGTTTACCCCCCG<br>CTCGCAGGCAATCTTGCCCATGCTGGGCCTTCTGTCGACTTAACCATCTTCTCCCTCCACTTGGC<br>CGGGGTGTCATCTATTCTAGGCGCAATTAATTTCATTACAACAATCATTAACATGAAACCCCCCG<br>CCATCTCTCAATATCAAACACCCCTATTTGTATGGTCCGTTCTAATTACCGCAGTATTACTTCTTC<br>TATCCCTACCCGTTCTTGCCGCCGGCATCACAATACTTCTCACAGACCGAAACCTAAACACAAC<br>CTTCTTTGATCCTGCCGGAGGAGGAGACCCCATCCTTTACCAACACTTA
MK497069	*O. niloticus*	TTGAGCCGGAATAGTAGGAACCGCGCTAAGCCTCCTAATTCGGGCAGAACTAAGCCAGCCCGG<br>CTCTCTCCTCGGAGACGACCAGATTTATAATGTAATTGTTACAGCACATGCTTTTGTAATAATTTT<br>CTTTATAGTAATGCCAATTATGATTGGAGGCTTTGGAAACTGACTAGTACCACTCATGATTGGTG<br>CCCCAGATATGGCCTTCCCTCGAATGAACAACATGAGTTTCTGACTCCTCCCTCCCTCATTCCTC<br>CTCCTCCTCGCCTCATCTGGAGTCGAAGCAGGTGCCGGCACAGGGTGAACTGTTTACCCCCCG<br>CTCGCAGGCAATCTTGCCCATGCTGGGCCTTCTGTCGACTTAACCATCTTCTCCCTCCACTTGGC<br>CGGGGTGTCATCTATTCTAGGCGCAATTAATTTCATTACAACAATCATTAACATGAAACCCCCCG<br>CCATCTCTCAATATCAAACACCCCTATTTGTATGGTCCGTTCTAATTACCGCAGTATTACTTCTTCT<br>ATCCCTACCCGTTCTTGCCGCCGGCATCACAATACTTCTCACAGACCGAAACCTAAACACAACC<br>TTCTTTGATCCTGCCGGAGGAGGAGACCCCATCCTTTACCAACACTTA
MK497076	*O. niloticus*	TTGAGCCGGAATAGTAGGAACCGCGCTAAGCCTCCTAATTCGGGCAGAACTACTCCAGCCCGG<br>CTCTCTCCTCGGAGACGACCAGATTTATAATGTAATTGTTACAGCACATGCTTTCGTAATAATTTT<br>CTTTATAGTAATGCCAATTATAATTGGAGGTTTTGGAAACTGACTAGTGCCACTAATGATTGGTG<br>CACCAGACATGGCCTTCCCTCGAATAAATAACATGAGTTTTTGACTCCTCCCCCCCTCATTTCTC<br>CTTCTCCTCGCCTCATCCGGGGTCGAAGCAGGGGCCGGTACAGGATGAACTGTTTATCCCCCAC<br>TCGCAGGCAATCTCGCCCATGCTGGGCCTTCCGTTGACTTAACCATCTTCTCCCTCCACTTGGCC<br>GGGGTGTCATCTATTTTAGGTGCAATTAATTTTATTACAACCATTATTAACATAAAACCCCCTGCC<br>ATCTCCCAATATCAAACACCCCTCTTTGTATGATCCGTTCTAATTACCGCAGTACTACTCCTACTA<br>TCCCTACCCGTTCTTGCCGCCGGCATCACAATACTTCTAACAGACCGAAACCTAAACACAACCT<br>TCTTTGATCCTGCCGGAGGAGGAGACCCCATCCTTTACCAACACTTA

MK497092	*O. niloticus*	TTGAGCCGGAATAGTAGGAACCGCGCTAAGCCTCCTAATTCGGGCAGAACTAAGCCAGCCCGG<br>CTCTCTCCTCGGAGACGACCAGATTTATAATGTAATTGTTACAGCACATGCTTTTGTAATAATTTT<br>CTTTATAGTAATGCCAATTATGATTGGAGGCTTTGGAAACTGACTAGTACCACTCATGATTGGTG<br>CCCCAGATATGGCCTTCCCTCGAATGAACAACATGAGTTTCTGACTCCTCCCTCCCTCATTCCTC<br>CTCCTCCTCGCCTCATCTGGAGTCGAAGCAGGTGCCGGCACAGGGTGAACTGTTTACCCCCCG<br>CTCGCAGGCAATCTTGCCCATGCTGGGCCTTCTGTCGACTTAACCATCTTCTCCCTCCACTTGGC<br>CGGGGTGTCATCTATTCTAGGCGCAATTAATTTCATTACAACAATCATTAACATGAAACCCCCCG<br>CCATCTCTCAATATCAAACACCCCTATTTGTATGGTCCGTTCTAATTACCGCAGTATTACTTCTTC<br>TATCCCTACCCGTTCTTGCCGCCGGCATCACAATACTTCTCACAGACCGAAACCTAAACACAAC<br>CTTCTTTGATCCTGCCGGAGGAGGAGACCCCATCCTTTACCAACACTTA
MK497097	*O. niloticus*	TTGAGCCGGAATAGTAGGAACTGCACTAAGCCTCCTAATTCGGGCAGAACTAAGCCAGCCCGG<br>CTCTCTCCTCGGAGACGACCAAATTTATAATGTAATTGTTACAGCACATGCTTTCGTAATAATTTT<br>CTTTATAGTAATACCAATTATGATTGGAGGCTTTGGAAACTGACTAGTACCACTCATGATTGGTG<br>CACCAGACATGGCCTTCCCTCGAATAAATAACATGAGCTTTTGACTTCTCCCCCCCTCATTTCTT<br>CTTCTTCTCGCCTCATCTGGAGTCGAAGCAGGTGCCGGCACAGGATGGACTGTTTATCCCCCGC<br>TCGCAGGCAATCTTGCCCACGCTGGACCTTCTGTTGACTTAACCATCTTCTCCCTCCACTTGGC<br>CGGGGTGTCATCTATTTTAGGTGCAATTAATTTTATCACAACCATTATTAACATGAAACCCCCTGC<br>CATCTCCCAATATCAAACACCCCTATTTGTGTGATCCGTCCTAATTACCGCAGTACTACTCCTTCT<br>ATCCCTGCCCGTTCTTGCCGCCGGCATCACAATACTTCTAACAGACCGAAACCTAAACACAACC<br>TTCTTTGACCCTGCCGGAGGAGGAGACCCCATCCTATACCAACACTTA
MK497135	*O. niloticus*	TTGAGCCGGAATAGAAGGAACTGCACTAAGCCTCCTAATTCGGGCAGAACTAAGCCAGCCCGG<br>CTCTCTTCTCGGAGACGACCAAATCTATAATGTAATTGTTACAGCACATGCTTTCGTAATAATTTT<br>CTTTATAGTAATACCAATTATGATTGGAGGCTTTGGAAACTGACTAGTACCCCTCATGATTGGTG<br>CACCAGACATGGCCTTCCCTCGAATAAATAACATGAGCTTTTGACTTCTCCCCCCCTCATTTCTT<br>CTTCTTCTCGCCTCATCTGGAGTCGAAGCAGGTGCCGGCACAGGATGGACTGTTTATCCCCCGC<br>TCGCAGGCAATCTTGCCCACGCTGGACCTTCTGTTGACTTAACCATCTTCTCCCTCCACTTGGC<br>CGGAGTGTCATCTATTTTAGGTGCAATTAATTTTATCACAACCATTATTAACATGAAACCCCCTGC<br>CATCTCCCAATATCAAACACCCCTATTTGTGTGATCCGTCCTAATTACCGCAGTACTACTCCTTCT<br>ATCCCTGCCCGTTCTTGCCGCCGGCATCACAATACTTCTAACAGACCGAAACCTAAACACAACC<br>TTCTTTGACCCTGCCGGAGGAGGAGACCCCATCCTATACCAACACTTA
MK497136	*O. niloticus*	TTGAGCCGGAATAGTAGGAACTGCACTAAGCCTCCTAATTCGGGCAGAACTAAGCCAGCCCGG<br>CTCTCTTCTCGGAGACGACCAAATCTATAATGTAATTGTTACAGCACATGCTTTCGTAATAATTTT<br>CTTTATAGTAATACCAATTATGATTGGAGGCTTTGGAAACTGACTAGTACCCCTCATGATTGGTG<br>CACCAGACATGGCCTTCCCTCGAATAAATAACATGAGCTTTTGACTTCTCCCCCCCTCATTTCTT<br>CTTCTTCTCGCCTCATCTGGAGTCGAAGCAGGTGCCGGCACAGGATGGACTGTTTATCCCCCGC<br>TCGCAGGCAATCTTGCCCACGCTGGACCTTCTGTTGACTTAACCATCTTCTCCCTCCACTTGGC<br>CGGAGTGTCATCTATTTTAGGTGCAATTAATTTTATCACAACCATTATTAACATGAAACCCCCTGC<br>CATCTCCCAATATCAAACACCCCTATTTGTGTGATCCGTCCTAATTACCGCAGTACTACTCCTTCT<br>ATCCCTGCCCGTTCTTGCCGCCGGCATCACAATACTTCTAACAGACCGAAACCTAAACACAACC<br>TTCTTTGACCCTGCCGGAGGAGGAGACCCCATCCTATACCAACACTTA
MK497137	*O. niloticus*	TTGAGCCGGAATAGTAGGAACTGCACTAAGCCTCCTAATTCGGGCAGAACTAAGCCAGCCCGG<br>CTCTCTTCTCGGAGACGACCAAATCTATAATGTAATTGTTACAGCACATGCTTTCGTAATAATTTT<br>CTTTATAGTAATACCAATTATGATTGGAGGCTTTGGAAACTGACTAGTACCCCTCATGATTGGTG<br>CACCAGACATGGCCTTCCCTCGAATAAATAACATGAGCTTTTGACTTCTCCCCCCCTCATTTCTT<br>CTTCTTCTCGCCTCATCTGGAGTCGAAGCAGGTGCCGGCACAGGATGGACTGTTTATCCCCCGC<br>TCGCAGGCAATCTTGCCCACGCTGGACCTTCTGTTGACTTAACCATCTTCTCCCTCCACTTGGC<br>CGGAGTGTCATCTATTTTAGGTGCAATTAATTTTATCACAACCATTATTAACATGAAACCCCCTGC<br>CATCTCCCAATATCAAACACCCCTATTTGTGTGATCCGTCCTAATTACCGCAGTACTACTCCTTCT<br>ATCCCTGCCCGTTCTTGCCGCCGGCATCACAATACTTCTAACAGACCGAAACCTAAACACAACC<br>TTCTTTGACCCTGCCGGAGGAGGAGACCCCATCCTATACCAACACTTA
MK497138	*O. niloticus*	TTGAGCCGGAATAGAAGGAACTGCACTAAGCCTCCTAATTCGGGCAGAACTAAGCCAGCCCGG<br>CTCTCTTCTCGGAGACGACCAAATCTATAATGTAATTGTTACAGCACATGCTTTCGTAATAATTTT<br>CTTTATAGTAATACCAATTATGATTGGAGGCTTTGGAAACTGACTAGTACCCCTCATGATTGGTG<br>CACCAGACATGGCCTTCCCTCGAATAAATAACATGAGCTTTTGACTTCTCCCCCCCTCATTTCTT<br>CTTCTTCTCGCCTCATCTGGAGTCGAAGCAGGTGCCGGCACAGGATGGACTGTTTATCCCCCGC<br>TCGCAGGCAATCTTGCCCACGCTGGACCTTCTGTTGACTTAACCATCTTCTCCCTCCACTTGGC<br>CGGAGTGTCATCTATTTTAGGTGCAATTAATTTTATCACAACCATTATTAACATGAAACCCCCTGC<br>CATCTCCCAATATCAAACACCCCTATTTGTGTGATCCGTCCTAATTACCGCAGTACTACTCCTTCT<br>ATCCCTGCCCGTTCTTGCCGCCGGCATCACAATACTTCTAACAGACCGAAACCTAAACACAACC<br>TTCTTTGACCCTGCCGGAGGAGGAGACCCCATCCTATACCAACACTTA
MK497139	*O. niloticus*	TTGAGCCGGAATAGAAGGAACTGCACTAAGCCTCCTAATTCGGGCAGAACTAAGCCAGCCCGG<br>CTCTCTTCTCGGAGACGACCAAATCTATAATGTAATTGTTACAGCACATGCTTTCGTAATAATTTT<br>CTTTATAGTAATACCAATTATGATTGGAGGCTTTGGAAACTGACTAGTACCCCTCATGATTGGTG<br>CACCAGACATGGCCTTCCCTCGAATAAATAACATGAGCTTTTGACTTCTCCCCCCCTCATTTCTT<br>CTTCTTCTCGCCTCATCTGGAGTCGAAGCAGGTGCCGGCACAGGATGGACTGTTTATCCCCCGC<br>TCGCAGGCAATCTTGCCCACGCTGGACCTTCTGTTGACTTAACCATCTTCTCCCTCCACTTGGC<br>CGGAGTGTCATCTATTTTAGGTGCAATTAATTTTATCACAACCATTATTAACATGAAACCCCCTGC<br>CATCTCCCAATATCAAACACCCCTATTTGTGTGATCCGTCCTAATTACCGCAGTACTACTCCTTCT<br>ATCCCTGCCCGTTCTTGCCGCCGGCATCACAATACTTCTAACAGACCGAAACCTAAACACAACC<br>TTCTTTGACCCTGCCGGAGGAGGAGACCCCATCCTATACCAACACTTA

MK497145	*O. niloticus*	TTGAGCCGGAATAGAAGGAACCGCGCTAAGCCTCCTAATTCGGGCAGAACTAACCCAGCCCGG<br>CTCTCTCCTCGGAGACGACCAGATTTATAATGTAATTGTTACAGCACATGCTTTTGTAATAATTTT<br>CTTTATAGTAATGCCAATTATGATTGGAGGCTTTGGAAACTGACTAGTACCACTCATGATTGGTG<br>CTCCAGATATGGCCTTCCCTCGAATGAACAACATGAGTTTCTGACTCCTCCCTCCCTCATTCCTC<br>CTCCTCCTCGCCTCATCTGGAGTCGAAGCAGGTGCCGGCACAGGGTGAACTGTTTACCCCCCG<br>CTCGCAGGCAATCTTGCCCATGCTGGGCCTTCTGTCGACTTAACCATCTTCTCCCTCCACTTGGC<br>CGGGGTGTCATCTATTCTAGGCGCAATTAATTTCATTACAACAATCATTAACATGAAACCCCCCG<br>CCATCTCTCAATATCAAACACCTCTATTTGTATGGTCCGTTCTAATTACCGCAGTATTACTTCTTC<br>TATCCCTACCCGTTCTTGCCGCCGGCATCACAATACTTCTCACAGACCGAAACCTAAACACAAC<br>CTTCTTTGATCCTGCCGGAGGAGGAGACCCCATCCTTTACCAACACTTA
MK497146	*O. niloticus*	TTGAGCCGGAATAGTAGGAACCGCGCTAAGCCTCCTAATTCGGGCAGAACTAAGCCAGCCCGG<br>CTCTCTCCTCGGAGACGACCAGATTTATAATGTAATTGTTACAGCACATGCTTTTGTAATAATTTT<br>CTTTATAGTAATGCCAATTATGATTGGAGGCTTTGGAAACTGACTAGTACCACTCATGATTGGTG<br>CCCCAGATATGGCCTTCCCTCGAATGAACAACATGAGTTTCTGACTCCTCCCTCCCTCATTCCTC<br>CTCCTCCTCGCCTCATCTGGAGTCGAAGCAGGTGCCGGCACAGGGTGAACTGTTTACCCCCCG<br>CTCGCAGGCAATCTTGCCCATGCTGGGCCTTCTGTCGACTTAACCATCTTCTCCCTCCACTTGGC<br>CGGGGTGTCATCTATTCTAGGCGCAATTAATTTCATTACAACAATCATTAACATGAAACCCCCCG<br>CCATCTCTCAATATCAAACACCCCTATTTGTATGGTCCGTTCTAATTACCGCAGTATTACTTCTTC<br>TATCCCTACCCGTTCTTGCCGCCGGCATCACAATACTTCTCACAGACCGAAACCTAAACACAAC<br>CTTCTTTGATCCTGCCGGAGGAGGAGACCCCATCCTTTACCAACACTTA
MK497147	*O. niloticus*	TTGAGCCGGAATAGTAGGAACCGCGCTAAGCCTCCTAATTCGGGCAGAACTAAGCCAGCCCGG<br>CTCTCTCCTCGGAGACGACCAGATTTATAATGTAATTGTTACAGCACATGCTTTTGTAATAATTTT<br>CTTTATAGTAATGCCAATTATGATTGGAGGCTTTGGAAACTGACTAGTACCACTCATGATTGGTG<br>CCCCAGATATGGCCTTCCCTCGAATGAACAACATGAGTTTCTGACTCCTCCCTCCCTCATTCCTC<br>CTCCTCCTCGCCTCATCTGGAGTCGAAGCAGGTGCCGGCACAGGGTGAACTGTTTACCCCCCG<br>CTCGCAGGCAATCTTGCCCATGCTGGGCCTTCTGTCGACTTAACCATCTTCTCCCTCCACTTGGC<br>CGGGGTGTCATCTATTCTAGGCGCAATTAATTTCATTACAACAATCATTAACATGAAACCCCCCG<br>CCATCTCTCAATATCAAACACCCCTATTTGTATGGTCCGTTCTAATTACCGCAGTATTACTTCTTC<br>TATCCCTACCCGTTCTTGCCGCCGGCATCACAATACTTCTCACAGACCGAAACCTAAACACAAC<br>CTTCTTTGATCCTGCCGGAGGAGGAGACCCCATCCTTTACCAACACTTA
MK497148	*O. niloticus*	TTGAGCCGGAATAGTAGGAACCGCGCTAAGCCTCCTAATTCGGGCAGAACTAAGCCAGCCCGG<br>CTCTCTCCTCGGAGACGACCAGATTTATAATGTAATTGTTACAGCACATGCTTTTGTAATAATTTT<br>CTTTATAGTAATGCCAATTATGATTGGAGGCTTTGGAAACTGACTAGTACCACTCATGATTGGTG<br>CCCCAGATATGGCCTTCCCTCGAATGAACAACATGAGTTTCTGACTCCTCCCTCCCTCATTCCTC<br>CTCCTCCTCGCCTCATCTGGAGTCGAAGCAGGTGCCGGCACAGGGTGAACTGTTTACCCCCCG<br>CTCGCAGGCAATCTTGCCCATGCTGGGCCTTCTGTCGACTTAACCATCTTCTCCCTCCACTTGGC<br>CGGGGTGTCATCTATTCTAGGCGCAATTAATTTCATTACAACAATCATTAACATGAAACCCCCCG<br>CCATCTCTCAATATCAAACACCCCTATTTGTATGGTCCGTTCTAATTACCGCAGTATTACTTCTTC<br>TATCCCTACCCGTTCTTGCCGCCGGCATCACAATACTTCTCACAGACCGAAACCTAAACACAAC<br>CTTCTTTGATCCTGCCGGAGGAGGAGACCCCATCCTTTACCAACACTTA
MK497149	*O. niloticus*	TTGAGCCGGAATAGTAGGAACCGCGCTAAGCCTCCTAATTCGGGCAGAACTAAGCCAGCCCGG<br>CTCTCTCCTCGGAGACGACCAGATTTATAATGTAATTGTTACAGCACATGCTTTTGTAATAATTTT<br>CTTTATAGTAATGCCAATTATGATTGGAGGCTTTGGAAACTGACTAGTACCACTCATGATTGGTG<br>CCCCAGATATGGCCTTCCCTCGAATGAACAACATGAGTTTCTGACTCCTCCCTCCCTCATTCCTC<br>CTCCTCCTCGCCTCATCTGGAGTCGAAGCAGGTGCCGGCACAGGGTGAACTGTTTACCCCCCG<br>CTCGCAGGCAATCTTGCCCATGCTGGGCCTTCTGTCGACTTAACCATCTTCTCCCTCCACTTGGC<br>CGGGGTGTCATCTATTCTAGGCGCAATTAATTTCATTACAACAATCATTAACATGAAACCCCCCG<br>CCATCTCTCAATATCAGACACCCCTATTTGTATGGTCCGTTCTAATTACCGCAGTATTACTTCTTC<br>TATCCCTACCCGTTCTTGCCGCCGGCATCACAATACTTCTCACAGACCGAAACCTAAACACAAC<br>CTTCTTTGATCCTGCCGGAGGAGGAGACCCCATCCTTTACCAACACTTA
MK497150	*O. niloticus*	TTGAGCCGGAATAGTAGGAACCGCGCTAAGCCTCCTAATTCGGGCAGAACTAAGCCAGCCCGG<br>CTCTCTCCTCGGAGACGACCAGATTTATAATGTAATTGTTACAGCACATGCTTTTGTAATAATTTT<br>CTTTATAGTAATGCCAATTATGATTGGAGGCTTTGGAAACTGACTAGTACCACTCATGATTGGTG<br>CCCCAGATATGGCCTTCCCTCGAATGAACAACATGAGTTTCTGACTCCTCCCTCCCTCATTCCTC<br>CTCCTCCTCGCCTCATCTGGAGTCGAAGCAGGTGCCGGCACAGGGTGAACTGTTTACCCCCCG<br>CTCGCAGGCAATCTTGCCCATGCTGGGCCTTCTGTCGACTTAACCATCTTCTCCCTCCACTTGGC<br>CGGGGTGTCATCTATTCTAGGCGCAATTAATTTCATTACAACAATCATTAACATGAAACCCCCCG<br>CCATCTCTCAATATCAAACACCCCTATTTGTATGGTCCGTTCTAATTACCGCAGTATTACTTCTTCT<br>ATCCCTACCCGTTCTTGCCGCCGGCATCACAATACTTCTCACAGACCGAAACCTAAACACAACC<br>TTCTTTGATCCTGCCGGAGGAGGAGACCCCATCCTTTACCAACACTTA
MK497151	*O. niloticus*	TTGAGCCGGAATAGTAGGAACCGCGCTAAGCCTCCTAATTCGGGCAGAACTAAGCCAGCCCGG<br>CTCTCTCCTCGGAGACGACCAGATTTATAATGTAATTGTTACAGCACATGCTTTTGTAATAATTTT<br>CTTTATAGTAATGCCAATTATGATTGGAGGCTTTGGAAACTGACTAGTACCACTCATGATTGGTG<br>CCCCAGATATGGCCTTCCCTCGAATGAACAACATGAGTTTCTGACTCCTCCCTCCCTCATTCCTC<br>CTCCTCCTCGCCTCATCTGGAGTCGAAGCAGGTGCCGGCACAGGGTGAACTGTTTACCCCCCG<br>CTCGCAGGCAATCTTGCCCATGCTGGGCCTTCTGTCGACTTAACCATCTTCTCCCTCCACTTGGC<br>CGGGGTGTCATCTATTCTAGGCGCAATTAATTTCATTACAACAATCATTAACATGAAACCCCCCG<br>CCATCTCTCAATATCAAACACCCCTATTTGTATGGTCCGTTCTAATTACCGCAGTATTACTTCTTC<br>TATCCCTACCCGTTCTTGCCGCCGGCATCACAATACTTCTCACAGACCGAAACCTAAACACAAC<br>CTTCTTTGATCCTGCCGGAGGAGGAGACCCCATCCTTTACCAACACTTA

MK497152	*O. niloticus*	TTGAGCCGGAATAGTAGGAACCGCGCTAAGCCTCCTAATTCGGGCAGAACTAAGCCAGCCCGG<br>CTCTCTCCTCGGAGACGACCAGATTTATAATGTAATTGTTACAGCACATGCTTTTGTAATAATTTT<br>CTTTATAGTAATGCCAATTATGATTGGAGGCTTTGGAAACTGACTAGTACCACTCATGATTGGTG<br>CCCCAGATATGGCCTTCCCTCGAATGAACAACATGAGTTTCTGACTCCTCCCTCCCTCATTCCTC<br>CTCCTCCTCGCCTCATCTGGAGTCGAAGCAGGTGCCGGCACAGGGTGAACTGTTTACCCCCCG<br>CTCGCAGGCAATCTTGCCCATGCTGGGCCTTCTGTCGACTTAACCATCTTCTCCCTCCACTTGGC<br>CGGGGTGTCATCTATTCTAGGCGCAATTAATTTCATTACAACAATCATTAACATGAAACCCCCCG<br>CCATCTCTCAATATCAAACACCCCTATTTGTATGGTCCGTTCTAATTACCGCAGTATTACTTCTTC<br>TATCCCTACCCGTTCTTGCCGCCGGCATCACAATACTTCTCACAGACCGAAACCTAAACACAAC<br>CTTCTTTGATCCTGCCGGAGGAGGAGACCCCATCCTTTACCAACACTTA
MK497153	*O. niloticus*	TTGAGCCGGAATAGTAGGAACCGCGCTAAGCCTCCTAATTCGGGCAGAACTAAGCCAGCCCGG<br>CTCTCTCCTCGGAGACGACCAGATTTATAATGTAATTGTTACAGCACATGCTTTTGTAATAATTTT<br>CTTTATAGTAATGCCAATTATGATTGGAGGCTTTGGAAACTGACTAGTACCACTCATGATTGGTG<br>CCCCAGATATGGCCTTCCCTCGAATGAACAACATGAGTTTCTGACTCCTCCCTCCCTCATTCCTC<br>CTCCTCCTCGCCTCATCTGGAGTCGAAGCAGGTGCCGGCACAGGGTGAACTGTTTACCCCCCG<br>CTCGCAGGCAATCTTGCCCATGCTGGGCCTTCTGTCGACTTAACCATCTTCTCCCTCCACTTGGC<br>CGGGGTGTCATCTATTCTAGGCGCAATTAATTTCATTACAACAATCATTAACATGAAACCCCCCG<br>CCATCTCTCAATATCAAACACCCCTATTTGTATGGTCCGTTCTAATTACCGCAGTATTACTTCTTC<br>TATCCCTACCCGTTCTTGCCGCCGGCATCACAATACTTCTCACAGACCGAAACCTAAACACAAC<br>CTTCTTTGATCCTGCCGGAGGAGGAGACCCCATCCTTTACCAACACTTA
MK497155	*O. niloticus*	TTGAGCCGGAATAGTAGGAACCGCGCTAAGCCTCCTAATTCGGGCAGAACTAAGCCAGCCCGG<br>CTCTCTCCTCGGAGACGACCAGATTTATAATGTAATTGTTACAGCACATGCTTTTGTAATAATTTT<br>CTTTATAGTAATGCCAATTATGATTGGAGGCTTTGGAAACTGACTAGTACCACTCATGATTGGTG<br>CCCCAGATATGGCCTTCCCTCGAATGAACAACATGAGTTTCTGACTCCTCCCTCCCTCATTCCTC<br>CTCCTCCTCGCCTCATCTGGAGTCGAAGCAGGTGCCGGCACAGGGTGAACTGTTTACCCCCCG<br>CTCGCAGGCAATCTTGCCCATGCTGGACCTTCTGTCGACTTAACCATCTTCTCCCTCCACTTGGC<br>CGGGGTATCATCTATTTTAGGCGCAATTAATTTCATTACAACAATCATTAACATGAAACCCCCCG<br>CCATCTCTCAATATCAGACACCCCTATTTGTATGGTCCGTTCTAATTACAGCAGTATTACTTCTTC<br>TATCCCTACCCGTTCTTGCCGCCGGCATCACAATACTTCTCACAGACCGAAACCTAAACACAAC<br>CTTCTTTGATCCTGCCGGAGGAGGAGACCCCATCCTTTACCAACACTTA
MK572406	*O. niloticus*	TTGAGCCGGAATAGTAGGAACTGCACTAAGCCTCCTAATTCGGGCAGAACTAAGCCAGCCCGG<br>CTCTCTTCTCGGAGACGACCAAATCTATAATGTAATTGTTACAGCACATGCTTTCGTAATAATTTT<br>CTTTATAGTAATACCAATTATGATTGGAGGCTTTGGAAACTGACTAGTACCCCTCATGATTGGTG<br>CACCAGACATGGCCTTCCCTCGAATAAATAACATGAGCTTTTGACTTCTCCCCCCCTCATTTCTT<br>CTTCTTCTCGCCTCATCTGGAGTCGAAGCAGGTGCCGGCACAGGATGGACTGTTTATCCCCCGC<br>TCGCAGGCAATCTTGCCCACGCTGGACCTTCTGTTGACTTAACCATCTTCTCCCTCCACTTGGC<br>CGGAGTGTCATCTATTTTAGGTGCAATTAATTTTATCACAACCATTATTAACATGAAACCCCCTGC<br>CATCTCCCAATATCAAACACCCCTATTTGTGTGATCCGTCCTAATTACCGCAGTACTACTCCTTCT<br>ATCCCTGCCCGTTCTTGCCGCCGGCATCACAATACTTCTAACAGACCGAAACCTAAACACAACC<br>TTCTTTGACCCTGCCGGAGGAGGAGACCCCATCCTATACCAACACTTA
MN605507	*O. aureus*	TTGAGCCGGAATAGTAGGAACCGCGCTAAGCCTCCTAATTCGGGCAGAACTAAGCCAGCCCGG<br>CTCTCTCCTCGGAGACGACCAGATTTATAATGTAATTGTTACAGCACATGCTTTTGTAATAATTTT<br>CTTTATAGTAATGCCAATTATGATTGGAGGCTTTGGAAACTGACTAGTACCACTCATGATTGGTG<br>CCCCAGATATGGCCTTCCCTCGAATGAACAACATGAGTTTCTGACTCCTCCCTCCCTCATTCCTC<br>CTCCTCCTCGCCTCATCTGGAGTCGAAGCAGGTGCCGGCACAGGGTGAACTGTTTACCCCCCG<br>CTCGCAGGCAATCTTGCCCATGCTGGGCCTTCTGTCGACTTAACCATCTTCTCCCTCCACTTGGC<br>CGGGGTGTCATCTATTCTAGGCGCAATTAATTTCATTACAACAATCATTAACATGAAACCCCCCG<br>CCATCTCTCAATATCAAACACCCCTATTTGTATGGTCCGTTCTAATTACCGCAGTATTACTTCTTC<br>TATCCCTACCCGTTCTTGCCGCCGGCATCACAATACTTCTCACAGACCGAAACCTAAACACAAC<br>CTTCTTTGATCCTGCCGGAGGAGGAGACCCCATCCTTTACCAACACTTA
MT525052	*O. niloticus*	TTGAGCGGGAATAGTAGGAACTGCACTAAGCCTCCTAATTCGGGCAGAACTAAGCCAGCCCGG<br>CTCTCTTCTCGGAGACGACCAAATCTATAATGTAATTGTTACAGCACATGCTTTCGAAATAATTT<br>TCTTTATAGGAATACCAATTATGATTGGAGGCTTTGGAAACTGACTAGTACCCCTCATGATTGGT<br>GCACCAGACATGGCCTTCCCTCGAATAAATAACATGAGCTTTTGACTTCTCCCCCCCTCATTTCT<br>TCTTCTTCTCGCCTCATCTGGAGTCGAAGCAGGTGCCGGCACAGGATGGACTGTTTATCCCCCG<br>CTCGCAGGCAATCTTGCCCACGCTGGACCTTCTGTTGACTTAACCATCTTCTCCCTCCACTTGG<br>CCGGAGTGTCATCTATTTTAGGTGCAATTAATTTTATCACAACCATTATTAACATGAAACCCCCTG<br>CCATCTCCCAATATCAAACACCCCTATTTGTGTGATCCGTCCTAATTACCGCAGTACTACTCCTTC<br>TATCCCTGCCCGTTCTTGCCGCCGGCATCACAATACTTCTAACAGACCGAAACCTAAACACAAC<br>CTTCTTTGACCCTGCCGGAGGAGGAGACCCCATCCTATACCAACACTTA
MT525055	*O. niloticus*	TTGAGGCGGAATAGTAGGAACTGCACTAAGCCTCCTAATTCGGGCAGAACTAAGCCAGCCCGG<br>CTCTCTTCTCGGAGACGACCAAATCTATAATGTAATTGTTACAGCACATGCTTTCGTAATAATTTT<br>CTTTATAGTAATACCAATTATGATTGGAGGCTTTGGAAACTGACTAGTACCCCTCATGATTGGTG<br>CACCAGACATGGCCTTCCCTCGAATAAATAACATGAGCTTTTGACTTCTCCCCCCCTCATTTCTT<br>CTTCTTCTCGCCTCATCTGGAGTCGAAGCAGGTGCCGGCACAGGATGGACTGTTTATCCCCCGC<br>TCGCAGGCAATCTTGCCCACGCTGGACCTTCTGTTGACTTAACCATCTTCTCCCTCCACTTGGC<br>CGGAGTGTCATCTATTTTAGGTGCAATTAATTTTATCACAACCATTATTAACATGAAACCCCCTGC<br>CATCTCCCAATATCAAACACCCCTATTTGTGTGATCCGTCCTAATTACCGCAGTACTACTCCTTCT<br>ATCCCTGCCCGTTCTTGCCGCCGGCATCACAATACTTCTAACAGACCGAAACCTAAACACAACC<br>TTCTTTGACCCTGCCGGAGGAGGAGACCCCATCCTATACCAACACTTA

MW205900	*O. niloticus*	TTGAGCCGGAATAGTAGGAACTGCACTAAGCCTCCTAATTCGGGCAGAACTAAGCCAGCCCGG<br>CTCTCTTCTCGGAGACGACCAAATCTATAATGTAATTGTTACAGCACATGCTTTCGTAATAATTTT<br>CTTTATAGTAATACCAATTATGATTGGAGGCTTTGGAAACTGACTAGTACCCCTCATGATTGGTG<br>CACCAGACATGGCCTTCCCTCGAATAAATAACATGAGCTTTTGACTTCTCCCCCCCTCATTTCTT<br>CTTCTTCTCGCCTCATCTGGAGTCGAAGCAGGTGCCGGCACAGGATGGACTGTTTATCCCCCGC<br>TCGCAGGCAATCTTGCCCACGCTGGACCTTCTGTTGACTTAACCATCTTCTCCCTCCACTTGGC<br>CGGAGTGTCATCTATTTTAGGTGCAATTAATTTTATCACAACCATTATTAACATGAAACCCCCTGC<br>CATCTCCCAATATCAAACACCCCTATTTGTGTGATCCGTCCTAATTACCGCAGTACTACTCCTTCT<br>ATCCCTGCCCGTTCTTGCCGCCGGCATCACAATACTTCTAACAGACCGAAACCTAAACACAACC<br>TTCTTTGACCCTGCCGGAGGAGGAGACCCCATCCTATACCAACACTTA
MW205901	*O. niloticus*	TTGAGCCGGAATAGTAGGAACTGCACTAAGCCTCCTAATTCGGGCAGAACTAAGCCAGCCCGG<br>CTCTCTTCTCGGAGACGACCAAATCTATAATGTAATTGTTACAGCACATGCTTTCGTAATAATTTT<br>CTTTATAGTAATACCAATTATGATTGGAGGCTTTGGAAACTGACTAGTACCCCTCATGATTGGTG<br>CACCAGACATGGCCTTCCCTCGAATAAATAACATGAGCTTTTGACTTCTCCCCCCCTCATTTCTT<br>CTTCTTCTCGCCTCATCTGGAGTCGAAGCAGGTGCCGGCACAGGATGGACTGTTTATCCCCCGC<br>TCGCAGGCAATCTTGCCCACGCTGGACCTTCTGTTGACTTAACCATCTTCTCCCTCCACTTGGC<br>CGGAGTGTCATCTATTTTAGGTGCAATTAATTTTATCACAACCATTATTAACATGAAACCCCCTGC<br>CATCTCCCAATATCAAACACCCCTATTTGTGTGATCCGTCCTAATTACCGCAGTACTACTCCTTCT<br>ATCCCTGCCCGTTCTTGCCGCCGGCATCACAATACTTCTAACAGACCGAAACCTAAACACAACC<br>TTCTTTGACCCTGCCGGAGGAGGAGACCCCATCCTATACCAACACTTA
MW205902	*O. niloticus*	TTGAGCCGGAATAGTAGGAACTGCACTAAGCCTCCTAATTCGGGCAGAACTAAGCCAGCCCGG<br>CTCTCTTCTCGGAGACGACCAAATCTATAATGTAATTGTTACAGCACATGCTTTCGTAATAATTTT<br>CTTTATAGTAATACCAATTATGATTGGAGGCTTTGGAAACTGACTAGTACCCCTCATGATTGGTG<br>CACCAGACATGGCCTTCCCTCGAATAAATAACATGAGCTTTTGACTTCTCCCCCCCTCATTTCTT<br>CTTCTTCTCGCCTCATCTGGAGTCGAAGCAGGTGCCGGCACAGGATGGACTGTTTATCCCCCGC<br>TCGCAGGCAATCTTGCCCACGCTGGACCTTCTGTTGACTTAACCATCTTCTCCCTCCACTTGGC<br>CGGAGTGTCATCTATTTTAGGTGCAATTAATTTTATCACAACCATTATTAACATGAAACCCCCTGC<br>CATCTCCCAATATCAAACACCCCTATTTGTGTGATCCGTCCTAATTACCGCAGTACTACTCCTTCT<br>ATCCCTGCCCGTTCTTGCCGCCGGCATCACAATACTTCTAACAGACCGAAACCTAAACACAACC<br>TTCTTTGACCCTGCCGGAGGAGGAGACCCCATCCTATACCAACACTTA
MW205903	*O. niloticus*	TTGAGCCGGAATAGTAGGAACTGCACTAAGCCTCCTAATTCGGGCAGAACTAAGCCAGCCCGG<br>CTCTCTTCTCGGAGACGACCAAATCTATAATGTAATTGTTACAGCACATGCTTTCGTAATAATTTT<br>CTTTATAGTAATACCAATTATGATTGGAGGCTTTGGAAACTGACTAGTACCCCTCATGATTGGTG<br>CACCAGACATGGCCTTCCCTCGAATAAATAACATGAGCTTTTGACTTCTCCCCCCCTCATTTCTT<br>CTTCTTCTCGCCTCATCTGGAGTCGAAGCAGGTGCCGGCACAGGATGGACTGTTTATCCCCCGC<br>TCGCAGGCAATCTTGCCCACGCTGGACCTTCTGTTGACTTAACCATCTTCTCCCTCCACTTGGC<br>CGGAGTGTCATCTATTTTAGGTGCAATTAATTTTATCACAACCATTATTAACATGAAACCCCCTGC<br>CATCTCCCAATATCAAACACCCCTATTTGTGTGATCCGTCCTAATTACCGCAGTACTACTCCTTCT<br>ATCCCTGCCCGTTCTTGCCGCCGGCATCACAATACTTCTAACAGACCGAAACCTAAACACAACC<br>TTCTTTGACCCTGCCGGAGGAGGAGACCCCATCCTATACCAACACTTA
MW205904	*O. niloticus*	TTGAGCCGGAATAGTAGGAACTGCACTAAGCCTCCTAATTCGGGCAGAACTAAGCCAGCCCGG<br>CTCTCTTCTCGGAGACGACCAAATCTATAATGTAATTGTTACAGCACATGCTTTCGTAATAATTTT<br>CTTTATAGTAATACCAATTATGATTGGAGGCTTTGGAAACTGACTAGTACCCCTCATGATTGGTG<br>CACCAGACATGGCCTTCCCTCGAATAAATAACATGAGCTTTTGACTTCTCCCCCCCTCATTTCTT<br>CTTCTTCTCGCCTCATCTGGAGTCGAAGCAGGTGCCGGCACAGGATGGACTGTTTATCCCCCGC<br>TCGCAGGCAATCTTGCCCACGCTGGACCTTCTGTTGACTTAACCATCTTCTCCCTCCACTTGGC<br>CGGAGTGTCATCTATTTTAGGTGCAATTAATTTTATCACAACCATTATTAACATGAAACCCCCTGC<br>CATCTCCCAATATCAAACACCCCTATTTGTGTGATCCGTCCTAATTACCGCAGTACTACTCCTTCT<br>ATCCCTGCCCGTTCTTGCCGCCGGCATCACAATACTTCTAACAGACCGAAACCTAAACACAACC<br>TTCTTTGACCCTGCCGGAGGAGGAGACCCCATCCTATACCAACACTTA
MW206001.1	*O. niloticus*	TTGAGCCGGAATAGTAGGAACTGCACTAAGCCTCCTAATTCGGGCAGAACTAAGCCAGCCCGG<br>CTCTCTTCTCGGAGACGACCAAATCTATAATGTAATTGTTACAGCACATGCTTTCGTAATAATTTT<br>CTTTATAGTAATACCAATTATGATTGGAGGCTTTGGAAACTGACTAGTACCCCTCATGATTGGTG<br>CACCAGACATGGCCTTCCCTCGAATAAATAACATGAGCTTTTGACTTCTCCCCCCCTCATTTCTT<br>CTTCTTCTCGCCTCATCTGGAGTCGAAGCAGGTGCCGGCACAGGATGGACTGTTTATCCCCCGC<br>TCGCAGGCAATCTTGCCCACGCTGGACCTTCTGTTGACTTAACCATCTTCTCCCTCCACTTGGC<br>CGGAGTGTCATCTATTTTAGGTGCAATTAATTTTATCACAACCATTATTAACATGAAACCCCCTGC<br>CATCTCCCAATATCAAACACCCCTATTTGTGTGATCCGTCCTAATTACCGCAGTACTACTCCTTCT<br>ATCCCTGCCCGTTCTTGCCGCCGGCATCACAATACTTCTAACAGACCGAAACCTAAACACAACC<br>TTCTTTGACCCTGCCGGAGGAGGAGACCCCATCCTATACCAACACTTA
JF510525	*Tilapia zillii*	TTGGGCCGGAATAGTAGGAACTGCACTCAGCCTCCTGATCCGAGCAGAACTAAGCCAGCCCGG<br>CTCCCTCCTCGGGGACGACCAGATTTATAATGTAATTGTTACAGCACATGCCTTTGTAATAATTTT<br>CTTTATAGTAATGCCAATTATGATTGGAGGCTTTGGGAACTGATTAGTCCCACTTATGATCGGCG<br>CACCAGACATGGCCTTCCCTCGAATAAACAACATAAGCTTCTGACTTCTCCCCCCATCATTCCTC<br>CTTCTCCTCGCTTCATCAGGGGTCGAAGCGGGCGCCGGTACAGGGTGAACTGTCTACCCCCCA<br>CTCGCAGGCAATCTCGCCCACGCTGGGCCCTCTGTTGACCTAACCATCTTCTCACTCCACCTAG<br>CCGGGGTATCATCTATTCTAGGGGCAATTAATTTCATTACAACAATTATTAACATGAAGCCCCCTG<br>CTATCTCTCAGTACCAAACGCCCCTGTTCGTGTGGTCCGTCCTAATTACGGCGGTGCTGCTTCTT<br>CTGTCACTGCCTGTTCTTGCTGCCGGCATTACAATGCTTCTCACAGACCGAAATCTAAACACGA<br>CCTTCTTTGATCCTGCCGGGGGAGGGGATCCCATCCTCTACCAACATTTA

Haplotype 1	*O. niloticus*	TTGAGCCGG-ATAGTAGGAACTGCACTAAGTCTCCTTATTCGGGCAGAACTAAGCCAGCCCGGC<br>TCTCTCCTCGGAGACGACCAGATTTATAATGTAATTGTTACAGCACATGCTTTCGTAATAATTTTC<br>TTTATAGTAATACCAATTATAATTGGAGGGTTTGGAAACTGACTGGTGCCACTTATGATTGGAGC<br>ACCAGACATGGCCTTCCCTCGAATAAATAACATGAGTTTTTGACTCCTTCCCCCCTCATTTCTCC<br>TTCTCCTCGCCTCATCCGGAGTCGAAGCAGGAGCCGGTACAGGATGAACTGTTTATCCTCCCCT<br>CGCAGGCAATCTCGCCCACGCTGGACCTTCTGTTGACTTGACCATCTTTTCCCTCCACTTGGCC<br>GGGGTGTCATCTATTTTAGGCGCAATTAATTTTATCACAACCATTATTAACATAAAACCCCCTGCC<br>ATCTCTCAATATCAAACACCCCTCTTTGTGTGATCCGTTCTAATTACCGCAGTATTACTCCTACTA<br>TCCCTGCCCGTTCTTGCCGCCGGCATCACAATACTTCTAACAGACCGAAACCTAAACACAACCT<br>TCTTTGACCCTGCCGGAGGAGGAGACCCCATCCTTTACCAACACTTA
Haplotype 2	*O. aureus*	TTGAGCCGG-ATAGTAGGAACCGCGCTAAGCCTCCTAATTCGGGCAGAACTAAGCCAGCCCGGC<br>TCTCTCCTCGGAGACGACCAGATTTATAATGTAATTGTTACAGCACATGCTTTTGTAATAATTTTC<br>TTTATAGTAATGCCAATTATGATTGGAGGCTTTGGAAACTGACTAGTACCACTCATGATTGGTGC<br>CCCAGATATGGCCTTCCCTCGAATGAACAACATGAGTTTCTGACTCCTCCCTCCCTCATTCCTCC<br>TCCTCCTCGCCTCATCTGGAGTCGAAGCAGGTGCCGGCACAGGGTGAACTGTTTACCCCCCGC<br>TCGCAGGCAATCTTGCCCATGCTGGGCCTTCTGTCGACTTAACCATCTTCTCCCTCCACTTGGCC<br>GGGGTGTCATCTATTCTAGGCGCAATTAATTTCATTACAACAATCATTAACATGAAACCCCCCGC<br>CATCTCTCAATATCAAACACCCCTATTTGTATGGTCCGTTCTAATTACCGCAGTATTACTTCTTCTA<br>TCCCTACCCGTTCTTGCCGCCGGCATCACAATACTTCTCACAGACCGAAACCTAAACACAACCT<br>TCTTTGATCCTGCCGGAGGAGGAGACCCCATCCTTTACCAACACTTA
